# Comparative transcriptional profiling of tildipirosin-resistant and sensitive *Haemophilus parasuis*

**DOI:** 10.1038/s41598-017-07972-5

**Published:** 2017-08-08

**Authors:** Zhixin Lei, Shulin Fu, Bing Yang, Qianying Liu, Saeed Ahmed, Lei Xu, Jincheng Xiong, Jiyue Cao, Yinsheng Qiu

**Affiliations:** 1 Veterinary Pharmacology Laboratory, College of Veterinary Medicine, Huazhong Agricultural University, Wuhan, 430070 P.R. China; 2 National Reference Laboratory of Veterinary Drug Residues and MAO Key Laboratory for Detection of Veterinary Drug Residues, Huazhong Agriculture University, Wuhan, 430070 P.R. China; 3 School of Animal Science and Nutritional Engineering, Wuhan Polytechnic University, Wuhan, 430023 P.R. China

## Abstract

Numerous studies have been conducted to examine the molecular mechanism of *Haemophilus parasuis* resistance to antibiotic, but rarely to tildipirosin. In the current study, transcriptional profiling was applied to analyse the variation in gene expression of *JS0135* and tildipirosin-resistant *JS32*. The growth curves showed that *JS32* had a higher growth rate but fewer bacteria than *JS0135*. The cell membranes of *JS32* and a resistant clinical isolate (*HB32*) were observed to be smoother than those of *JS0135*. From the comparative gene expression profile 349 up- and 113 downregulated genes were observed, covering 37 GO and 63 KEGG pathways which are involved in biological processes (11), cellular components (17), molecular function (9), cellular processes (1), environmental information processing (4), genetic information processing (9) and metabolism (49) affected in *JS32*. In addition, the relative overexpression of genes of the metabolism pathway (*HAPS_RS09315*, *HAPS_RS09320*), ribosomes (*HAPS_RS07815*) and ABC transporters (*HAPS_RS10945*) was detected, particularly the metabolism pathway, and verified with RT-qPCR. Collectively, the gene expression profile in connection with tildipirosin resistance factors revealed unique and highly resistant determinants of *H. parasuis* to macrolides that warrant further attention due to the significant threat of bacterial resistance.

## Introduction

The pathogen *Haemophilus parasuis* (*HPS*) is among the most commonly identified Gram-negative bacteria mainly causing serofibrinous polyserositis and arthritis which leads to major economic losses in the swine industry worldwide^1–3^. Of the 15 serovars, serovars 4 and 5 are widely associated with epidemics and serovar 5 is particularly highly virulent in China^4^. Various antimicrobial agents, including macrolides, β-lactams, phenicols, potentiated sulfonamides and tetracyclines, have been administered for the treatment and prevention of respiratory infections caused by *HPS* ^5–7^. Antimicrobials were thought to be the most powerful and typical way to combat *HPS* invasion^8^. However, the prolonged exposure of pathogens to drugs can induce resistance^9, 10^. In recent years, clinical isolates resistant to antimicrobials have been reported in Switzerland, the United Kingdom and Spain. It was found that clinical *HPS* exhibited high and extensive resistance to enrofloxacin, trimethoprim, sulfamethoxazole, tilmicosin and tulathromycin^7, 11^.

Tildipirosin, a new 16-membered ring macrolide, is a semisynthetic tylosin developed to treat respiratory pathogens. However, the resistance of *Pasteurella multocida* (*PM*) to macrolides including tildipirosin, tilmicosin and gamithromycin has previously been reported. Several resistant genes have been identified, such as *msr(E)*, *mph(E)* and *erm(42)* ^12, 13^. *HPS*, belongs to the order Pasteurellales of family Pasteurellaceae which is made up of at least 15 genera and over 70 species^14^, has also been isolated from diseased swine and identified with different levels of sensitivity (MIC, minimal inhibitory concentration) to tildipirosin^15^. The resistance characteristics of *HPS* to different antibiotics including fluoroquinolone, marcolides, tetracycline and beta-lactam has been investigated in previously described reports and some classical resistant genes such as acrAB, Tet B, Tet A, ErmB, etc^16–19^. The resistance mechanism of *HPS* to macrolides has been associated with pathways of the amino acid ATP-binding cassette (ABC) transport system (*HAPS_2069*) and the metabolite transporter superfamily (*HAPS_2067*, *HAPS_2068*). However, no studies have been conducted on the mechanisms of tildipirosin resistance in *HPS*. In the current study, several resistance *HPS* strains were isolated in diseased swine and induced in lab, and a transcriptomic approach was applied to achieve a genetically tildipirosin-resistant characteristic and revealed promising therapeutic targets to combat resistance^20^.

Transcriptional profiling is a useful tool for rapidly and simultaneously identifying large numbers of genetic determinants. Transcriptional profiling analysis provides distinct and detailed genomic-level information related to specific pathogenic mechanisms involving virulence factors and resistance genes^8, 21^. The extent of bacterial mechanistic response to antibiotic invasion has been revealed to be time- or dose-dependent in previous reports^22, 23^. Thus, a systematic approach of transcriptional profiling may aid the discovery of the resistance mechanisms of *HPS* to tildipirosin.

The objective of this study was therefore to use an RNA sequence method to systematically analyse the altered response of the tildipirosin-resistant strain’s (*JS32*) transcriptome and morphological characteristics compared to *JS0135*. These findings will help us to better understand the tildipirosin resistance mechanism in *HPS* which could then contribute to reasonable administration of tildipirosin and the development of methods used to prevent or reduce resistance in *HPS*.

## Results

### Minimal inhibitory concentration (MIC) determination, growth comparison and transmission electron microscope (TEM) analysis

*JS32* is a tildipirosin-resistant strain which was obtained after exposure to progressive concentrations of tildipirosin as described in detail in the experimental procedures. *HB32* was obtained from clinical isolation. The MICs of *JS0135*, *JS32* and *HB32* were 0.125, 32 and 32 μg/ml respectively, determined with broth microdilution assays. When *JS0135* was exposed to tildipirosin, it exhibited increased resistance (MIC ≥ 128 μg/ml). However, the high level of resistance was not maintained after a single passage of cells in growth medium without tildipirosin. *JS32* kept stable resistance (MIC = 32 μg/ml). The serovars of *JS0135*, *JS32* and *HB32* were amplified by PCR with the appropriate primers listed in Table 1 and were identified as serovars 4, 4 (320 bp) and 13 (840 bp), respectively (Supplementary Figure S1).

**Table 1:** Primers of RT-qPCR and serotype.

Gene		Nucleotide sequence (5′–3′)	Tm (°C)	Length (bp)
HAPS_RS09315	Forward	CAGCTCCAGCAAGAACTACA	54.6	177
	Reverse	AAGTCTCACTGGAGCCTGGT	57.4	
HAPS_RS09320	Forward	ATTGCATCTCCCCCTTGTCA	56.0	285
	Reverse	TTGTGGCGTCCCATAGTCTG	56.8	
glmM	Forward	TGGCTAAAGCTGTGCCACT	56.7	203
	Reverse	TAAAGCCCCATCTTCGCACT	56.4	
HAPS_RS04930	Forward	CCAGTTGCAAGCCCTCAT	55.0	171
	Reverse	CCAGCTTCTTGGGCTAGTTG	55.6	
HAPS_RS03600	Forward	GGGCAGGTACAGACACAATC	55.3	207
	Reverse	TCACGTCCACTTGCATTCCT	56.6	
HAPS_RS07815	Forward	AAGGCCGTAACCGTGGTATC	56.9	109
	Reverse	CGAGCTGCTTCGATTTGACG	57.2	
HAPS_RS10945	Forward	TATGCAAATTCAGCTTTCTTTA	49.2	123
	Reverse	TTTACTCGGCTCCTGACA	52.5	
HAPS_RS03625	Forward	CGATCCGCTACGTCGTGTTA	57.1	268
	Reverse	GGTCGGTAGGGCATCATAGC	57.1	
HAPS_RS11130	Forward	TAGCTGGTTTAGGGGTTGCG	57.1	163
	Reverse	ATCTCGTCCCAAACGATCCG	57.0	
HAPS_RS06145	Forward	ACGCATTCTTTCGGCAATCG	57.1	127
	Reverse	AAACTGAGCCCATTCCCACA	56.5	
16 s rRNA	Forward	GAGCGCAACCCTTATCCTTTGTT	56.8	176
	Reverse	TCACTCTACCTCGCGGCTTCGTC	56.4	
wciP (serovar 4)	Forward	GGTTAAGAGGTAGAGCTAAGAATAGAGG	53.6	320
	Reverse	CTTTCCACAACAGCTCTAGAAACC	57.3	
wcwK (serovar 5)	Forward	CCACTGGATAGAGAGTGGCAGG	55.8	450
	Reverse	CCATACATCTGAATTCCTAAGC	59.2	
gltP (serovar 13)	Forward	GCTGGAGGAGTTGAAAGAGTTGTTAC	57.8	840
	Reverse	CAATCAAATGAAACAACAGGAAGC	53.2	

**Figure 1:**
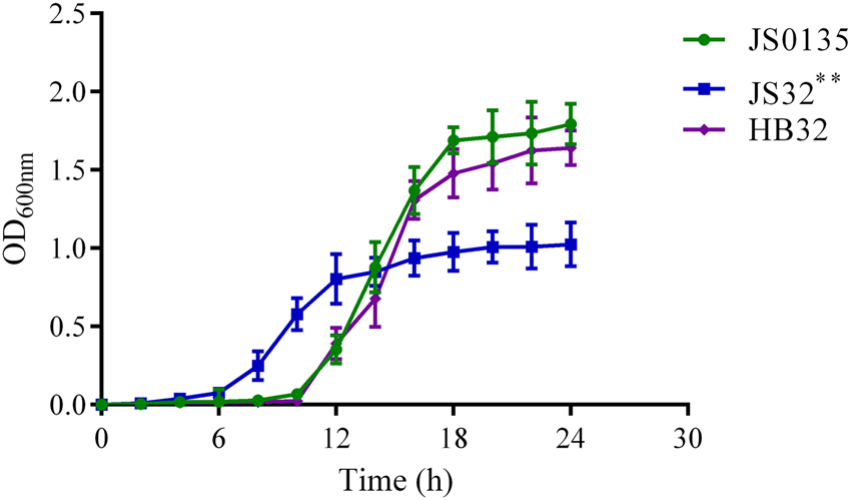
Growth curves of *JS0135*, *JS32* and *HB32*. *Presents statistically significant *p* ≤ 0.05, **presents extremely significant *p* ≤ 0.01.

The growth characteristics of *JS0135*, *JS32* and *HB32* were compared by measuring OD_600nm_ at different time points. No differences were observed between *JS0135* and *HB32*, but the growth rate of *JS32* was the fastest (Fig. 1). *JS32*, the induced tildipirosin-resistant strain, achieved logarithmic phase growth at 8 h, while *JS0135* and *HB32* did so at 12 h. Although the three strains entered into stationary phase at 18 h, the total bacteria count of *JS32* was significantly less than *JS0135* and *HB32* which was similar to previous research in response to tilmicosin^20^.

**Figure 2:**
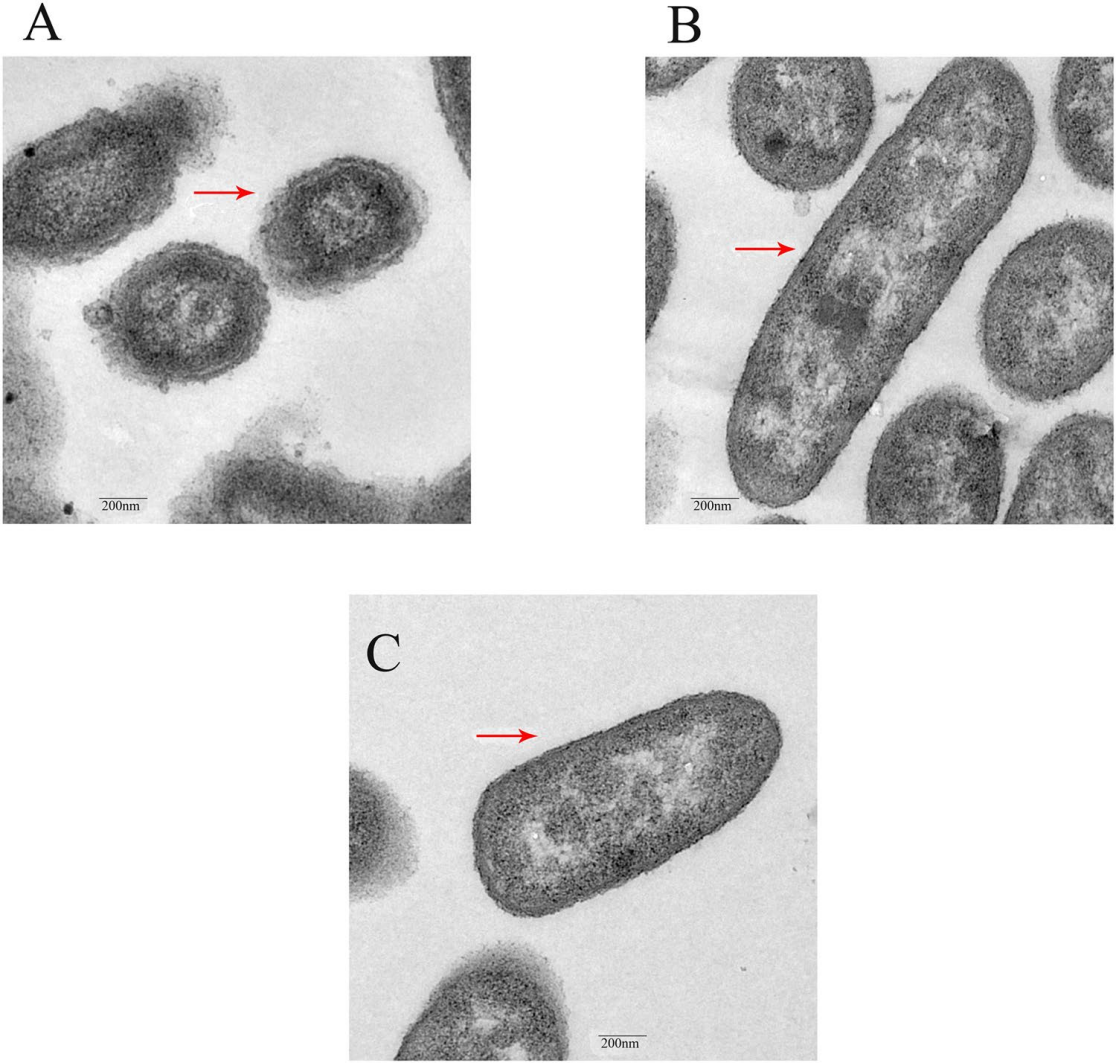
Comparison of transmission electron microscope: (**A**) presents *JS0135*, (**B**) presents *JS32*, (**C**) presents *HB32*. Red arrow pointed to the membrane of strains.

TEM was used to investigate morphologic diversity between sensitive cells (*JS0135*) and resistant cells (*JS32* and *HB32*). Three samples were collected at 12 h (exponential phase of growth), based on the growth curves. The TEM results showed that the membranes of induced (*JS32*) and wild-type (*HB32*) resistant bacteria had smoother margins than the control sensitive bacteria (*JS0135*), and the membrane of *JS32* was the smoothest among the three bacteria (Fig. 2). Similar changes between resistant and sensitive *HPS* were reported in previous research^8, 24^.

### Transcriptome sequencing annotation

A total of 18,620,015 ± 158,693 raw reads and 32,093,782 ± 791,754 reads with Q20 values of 93.46% ± 0.004 and 94.90% ± 0.011 in control (*JS0135*) and treatment groups (*JS32*), respectively; 15,966,164 ± 201,137 and 27,829,816 ± 1065685 (means ± SD) high-quality mapped reads were obtained in the control and treatment groups, respectively, and mapping ratios of 95.21% ± 0.001 and 96.14% ± 0.005 were obtained after filtering adapters and trimming ambiguous results (Table 2). Compared to the control group, the treatment group (*JS32*) had a significantly different increase (*p* ≤ 0.01) in raw reads, clean reads, all reads and mapped reads, but no differences in Q20 value and mapping ratio.

**Table 2:** Statistical summary of RNA–seq datasets in *JS32* and *JS0135*.

Samples	Means and SD of Raw			Means and SD of Mapping		
	Raw reads	Clean reads	Q20 Value^a^	All reads(rRNA trimed)	Mapped reads	Mapping ratio^b^
JS0135	18,620,015 ± 158,693	17,402,802 ± 229,554	93.46% ± 0.004	16,770,268 ± 228,692	15,966,164 ± 201,137	95.21% ± 0.001
JS32	32,093,782 ± 791,754**	30,446,935 ± 401,131**	94.90% ± 0.011	28,943,195 ± 945,527**	27,829,816 ± 1065685**	96.14% ± 0.005

^a^The sequencing quality values correspond to 0.01 of error chance, ^b^Mapping ratio = Mapped reads/All reads, *represents statistically significant (*p* ≤ 0.05), **represents extremely significant (*p* ≤ 0.01).

### Differential expression and functional analysis of genes

**Figure 3:**
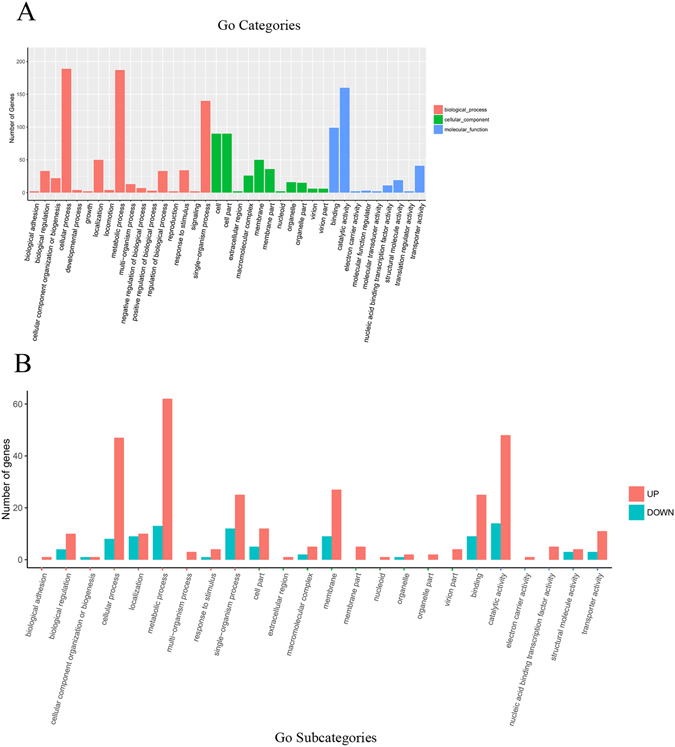
GO functional categories analysis (**A**), and up, down regulation of DE genes in subcategories statistics (**B**). A, the top groups in the three main categories: biological process (17), cellular component (11), molecular function (9) are summarized. The x-axis presents the categories, and the y-axis presents the number of genes in the categories. B, the number of up and down regulation genes are summarized in the subcategories belonging to the categories of A. Pink in X axis label represented biological process; green in the X axis label represented cellular component; blue in the X axis label represented molecular function.

Differential analysis of the transcript expression profiles revealed that 349 genes, including 41 novel genes, were upregulated (FC ≥ 2); 113 genes, including 10 novel genes, were dwonregulated (FC ≤ 0.5); and as a whole the treatment group (JS32) were more responsive than the control group (JS0135) (Supplementary Figure S2). The full list of DE transcripts can be seen in Supplementary File 1. GO classification and Kyoto Encyclopaedia of Genes and Genomes (KEGG) pathway analysis were performed as bioinformatics tools to explore the potential roles of DE genes in the resistance mechanism. Of 462 DE genes, 321 (69.7%) were assigned GO categories, and were further classified into three types: cellular component, biological process and molecular function (Fig. 3a). Within the biological process group, the most abundant categories were cellular process, metabolic process and single-organism process; other appealing categories included biological regulation, locomotion and signalling. In the cellular component group, cell part, cell membrane and membrane part were the most highly described subcategories. From those three groups, 17 subcategories were in the biological process group, 11 subcategories were in the cellular component group, and 9 subcategories were in the molecular function group, and up- and downregulation were shown in the subcategories (Fig. 3b and Supplementary File 2).

**Figure 4:**
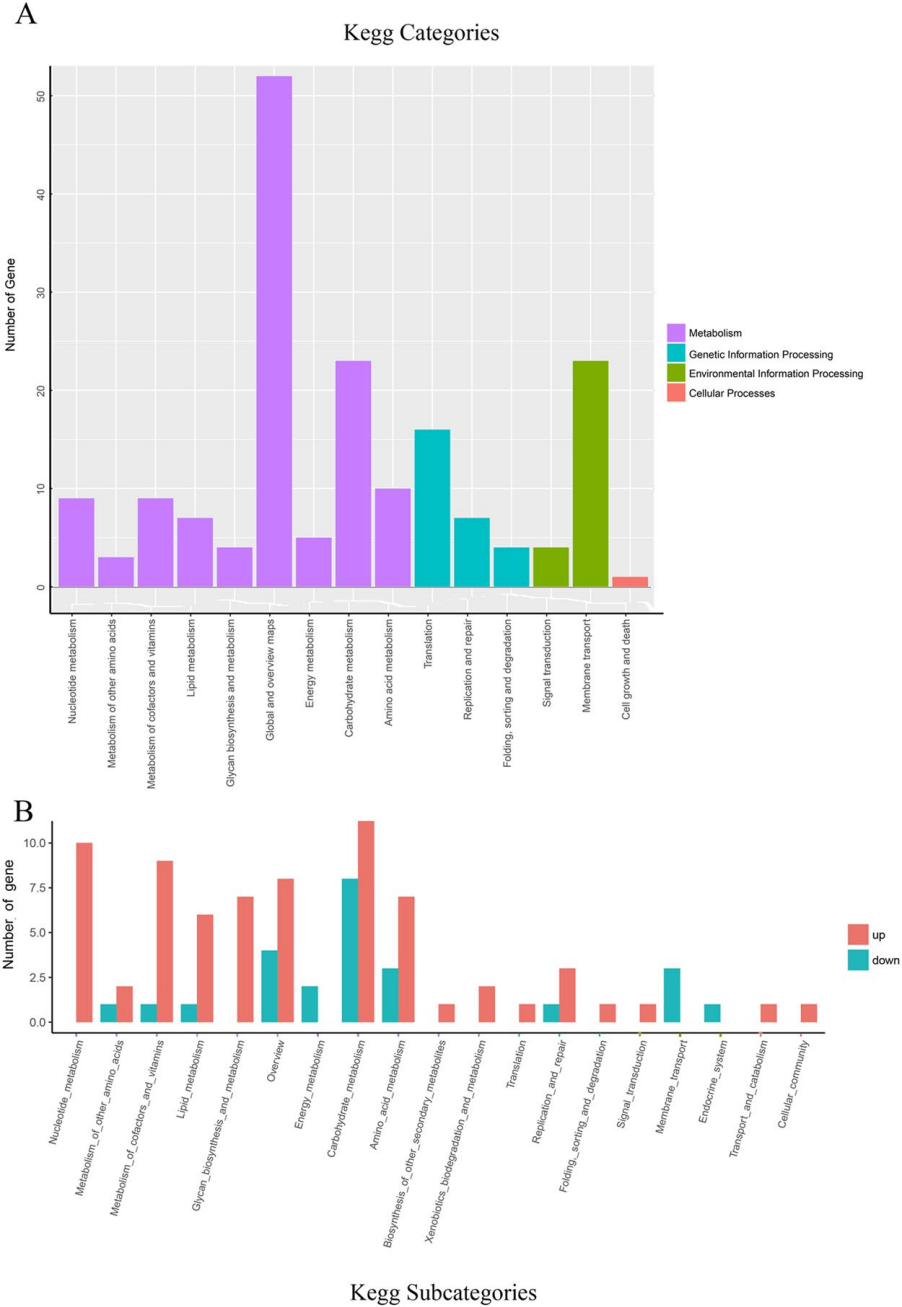
KEGG pathway classification analysis (**A**), and up, down regulation of DE genes in subcategories statistics (**B**). (**A**) The DE genes in the four pathways processes: metabolism (64), genetic information processes (27), environment information processes (27), cellular processes (1), are summarized. The x-axis presents categories pathways, and the y-axis presents the number genes in categories pathway. (**B**) the numbers of up and down regulation genes are summarized in the subcategories pathways belonging to the categories of A. Purple in the X axis label represented metabolism; blue in the X axis label represented genetic information processing; yellow in the X axis label represented environmental information processing; pink in the X axis label represented cellular processes.

According to the KEGG analysis, 116 DE genes were found to be classified into four parts and involved in 64 different pathways. From those four groups, one categories was in the cellular processes group, four categories were in the environmental information processing group, nine categories were in the genetic information processing group, 49 categories were in the metabolism group (Fig. 4a **)**, and up- and downregulation were in the subcategories (Fig. 4b and Supplementary File 3). The most abundant pathways in the KEGG analysis were metabolic pathways, biosynthesis of secondary metabolites, ribosomes, ABC transporters, biosynthesis of antibiotics, purine metabolism, microbial metabolism in diverse environments, quorum sensing and glycerophospholipid metabolism; other appealing pathways included aminoacyl-tRNA biosynthesis and cell cycle. Furthermore, the DE transcripts related to the GO and KEGG pathway results on resistance were involved in metabolism, ribosome, ABC transporters, metabolic pathways, the phosphotransferase system (PTS) and cationic antimicrobial peptide (CAMP) resistance. RNA-seq was displayed in Supplement File 1. In the total gene expression comparison of JS32 and JS0135, we selected resistance related genes with the value FC ≥ 2 or ≤ 0.5 (Tables 3 and 4).

**Table 3:** The important up regulation genes of JS32 compared to JS0135 grouped by GO and KEGG pathways of interest.

Gene	Product description	P-value	Fold change
**Metabolic pathways**			
HAPS_RS09315	restriction endonuclease subunit M	7.70E-301	Inf
HAPS_RS09320	DNA cytosine methyltransferase	0.00E + 00	Inf
HAPS_RS06285	phosphate acyltransferase	4.48E-19	3.40
HAPS_RS11130	phosphatidylglycerophosphatase A	8.85E-04	4.62
HAPS_RS06145	phosphatidate cytidylyltransferase	1.50E-08	2.24
HAPS_RS06125	phosphatidylglycerophosphatase	7.41E-05	2.04
glmM	phosphoglucosamine mutase	2.77E-11	2.60
HAPS_RS04930	beta-hexosaminidase	7.43E-17	4.02
HAPS_RS03600	UDP-N-acetylglucosamine 1-carboxyvinyltransferase	7.78E-06	2.09
HAPS_RS08950	thiamine phosphate synthase	1.46E-11	Inf
HAPS_RS08955	hydroxymethylpyrimidine/phosphomethylpyrimidine kinase	3.06E-12	Inf
HAPS_RS07450	myo-inosose-2 dehydratase	2.38E-37	6.39
HAPS_RS07445	3D-(3,5/4)-trihydroxycyclohexane-1,2-dione acylhydrolase (decyclizing)	1.83E-56	19.75
HAPS_RS04065	phosphogluconate dehydrogenase	2.69E-06	2.03
ilvH	acetolactate synthase small subunit	5.98E-03	2.35
HAPS_RS00040	fumarate reductase	6.68E-05	2.83
apaH	bis(5\-nucleosyl)-tetraphosphatase (symmetrical)	1.77E-04	2.01
HAPS_RS05675	anaerobic ribonucleoside-triphosphate reductase	1.20E-07	2.19
HAPS_RS09780	guanylate kinase	6.10E-04	2.21
dnaE	DNA polymerase III subunit alpha	1.15E-06	2.21
HAPS_RS01460	IMP dehydrogenase	9.93E-08	2.18
HAPS_RS09615	phosphoribosylformylglycinamidine synthase	1.18E-13	2.85
HAPS_RS07125	xanthine phosphoribosyltransferase	1.44E-07	6.35
HAPS_RS05080	PLP-dependent threonine dehydratase	1.64E-11	2.56
HAPS_RS08960	hydroxyethylthiazole kinase	1.22E-06	Inf
HAPS_RS09895	dihydroorotate dehydrogenase 2	1.14E-06	2.53
HAPS_RS06125	phosphatidylglycerophosphatase	7.41E-05	2.04
HAPS_RS04950	lipooligosaccharide D-glycero-D-manno-heptosyltransferase	3.14E-09	2.50
upp	uracil phosphoribosyltransferase	6.72E-16	3.23
HAPS_RS07455	inositol 2-dehydrogenase	3.73E-08	2.38
HAPS_RS07450	myo-inosose-2 dehydratase	2.38E-37	6.39
**Ribosome**			
HAPS_RS07815	MULTISPECIES: 50S ribosomal protein L16	4.98E-31	4.46
HAPS_RS07810	30S ribosomal protein S3	3.93E-23	3.80
HAPS_RS07825	30S ribosomal protein S17	2.24E-11	2.64
HAPS_RS07790	MULTISPECIES: 50S ribosomal protein L23	1.46E-21	3.44
rpsJ	MULTISPECIES: 30S ribosomal protein S10	7.68E-16	2.99
HAPS_RS07805	MULTISPECIES: 50S ribosomal protein L22	2.57E-23	3.71
HAPS_RS07795	50S ribosomal protein L2	6.51E-16	3.31
HAPS_RS07780	50S ribosomal protein L3	6.11E-17	3.20
rplD	50S ribosomal protein L4	5.49E-21	3.67
HAPS_RS07800	MULTISPECIES: 30S ribosomal protein S19	1.91E-18	3.43
HAPS_RS07820	MULTISPECIES: 50S ribosomal protein L29	8.16E-16	3.19
**ABC transporters**			
HAPS_RS10945	phosphonate ABC transporter permease	1.30E-02	2.51
HAPS_RS03625	ABC transporter permease	1.43E-05	2.35
HAPS_RS05335	membrane protein	0.02	3.03
HAPS_RS05330	manganese transporter	0.02	2.20
HAPS_RS03630	ABC transporter ATP-binding protein	6.61E-05	2.30
HAPS_RS00315	hypothetical protein	6.26E-03	3.41
HAPS_RS01125	cysteine/glutathione ABC transporter ATP-binding protein/permease CydC	1.43E-13	2.88
HAPS_RS04845	ABC transporter substrate-binding protein	1.50E-07	2.13
HAPS_RS00310	ABC transporter family protein	3.07E-03	2.62
HAPS_RS05165	arginine transporter permease subunit ArtQ	1.18E-02	2.05
HAPS_RS04855	peptide ABC transporter permease	3.44E-05	2.03
**CAMP resistance**			
HAPS_RS07240	hypothetical protein	4.61E-03	2.69
HAPS_RS11325	calcium-binding domain-containing protein	2.25E-04	2.06
HAPS_RS06175	acyl	4.96E-07	2.07

Note: Inf, represented infinity.

**Table 4:** The important down regulation genes of JS32 compared to JS0135 grouped by GO and KEGG pathways of interest.

Gene/Pathway	Product description	P-value	Fold change
**Phosphotransferase system (PTS)**			
HAPS_RS00970	PTS mannose transporter subunit IIAB	1.11E-65	0.16
HAPS_RS04655	PTS glucose transporter subunit IIA	6.63E-15	0.39
HAPS_RS04905	PTS sucrose transporter subunit IIBC	8.57E-38	0.17
HAPS_RS06060	hypothetical protein	5.60E-18	0.34
HAPS_RS00960	PTS fructose transporter subunit IID	7.69E-51	0.16
HAPS_RS00965	PTS fructose transporter subunit IIC	6.71E-50	0.20
**Metabolic pathways**			
HAPS_RS07375	glycerol-3-phosphate acyltransferase	4.07E-19	0.40
HAPS_RS09985	galactose-1-phosphate uridylyltransferase	3.50E-16	0.43
HAPS_RS00955	beta-galactosidase	1.84E-28	0.38
HAPS_RS05910	N-acetylmannosamine-6-phosphate 2-epimerase	3.35E-17	0.43
HAPS_RS06560	5-methyltetrahydropteroyltriglutamate–homocysteine S-methyltransferase	1.02E-20	0.35
HAPS_RS02470	S-adenosylmethionine synthase	1.84E-21	0.42
HAPS_RS10200	glutamate synthase subunit beta	2.29E-14	0.49
metF	5,10-methylenetetrahydrofolate reductase	1.57E-23	0.41
HAPS_RS07890	F0F1 ATP synthase subunit epsilon	4.18E-29	0.13
**Ribosome**			
rpmE	50S ribosomal protein L31	6.11E-17	0.48
HAPS_RS05815	MULTISPECIES: 30S ribosomal protein S21	1.11E-06	0.34
rpmH	MULTISPECIES: 50S ribosomal protein L34	2.28E-07	0.47
rpmG	MULTISPECIES: 50S ribosomal protein L33	1.52E-11	0.46
**ABC transporters**			
metN	D-methionine ABC transporter, ATP-binding protein	2.19E-32	0.35
HAPS_RS08310	hypothetical protein	6.04E-14	0.31
HAPS_RS02205	methionine ABC transporter permease	2.49E-15	0.46
metQ	membrane protein	5.79E-15	0.46
HAPS_RS07265	biotin transporter BioY	1.06E-30	0.31

Note: Inf, represented infinity.

### Search Tool for the Retrieval of Interacting Genes/Proteins (STRING) analysis of the relationships between DE genes of the main pathways

**Figure 5:**
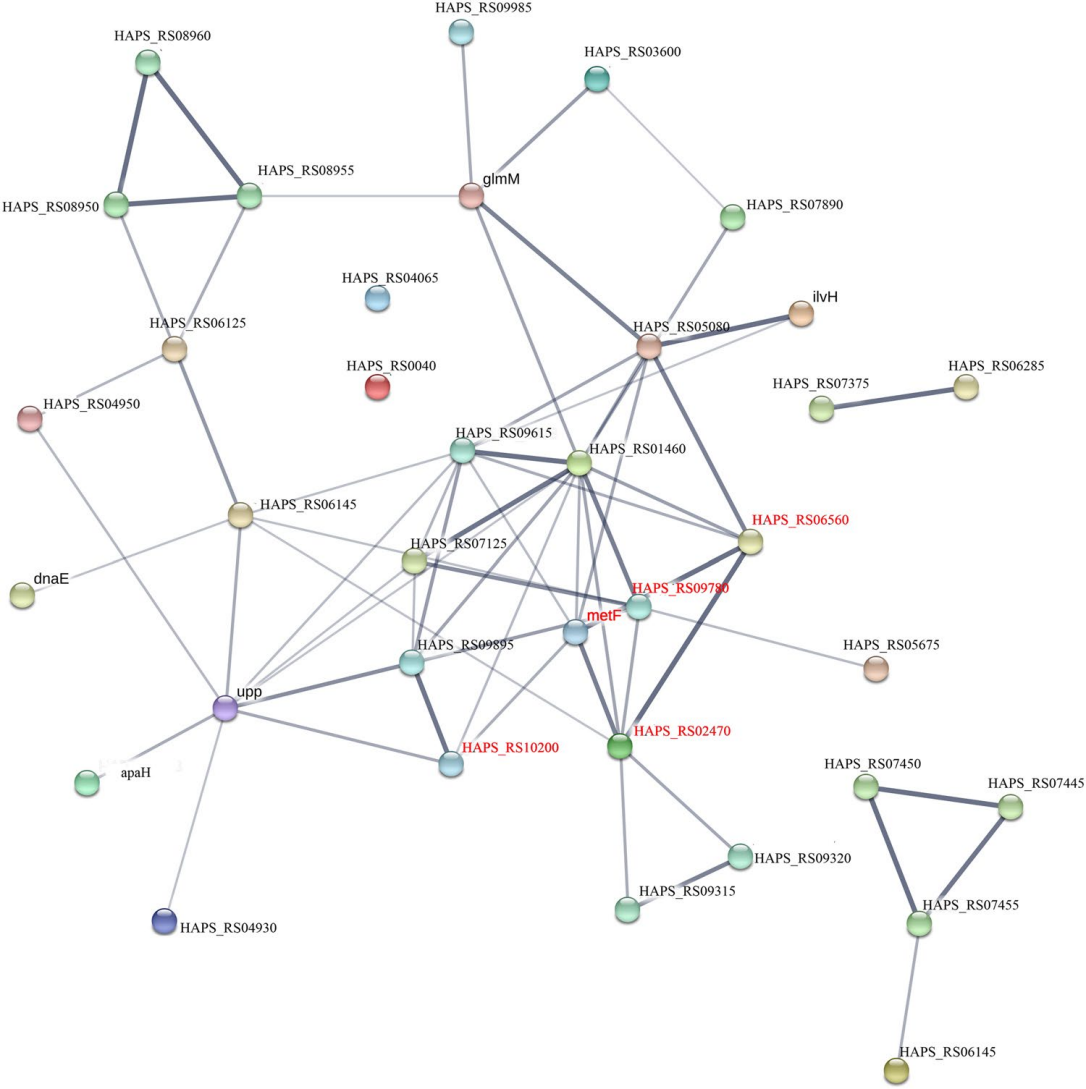
STRING analysis of the relationship between main 40 DE genes in metabolic pathways. The downregulated genes were marked with red, and the others were upregulated genes.

**Figure 6:**
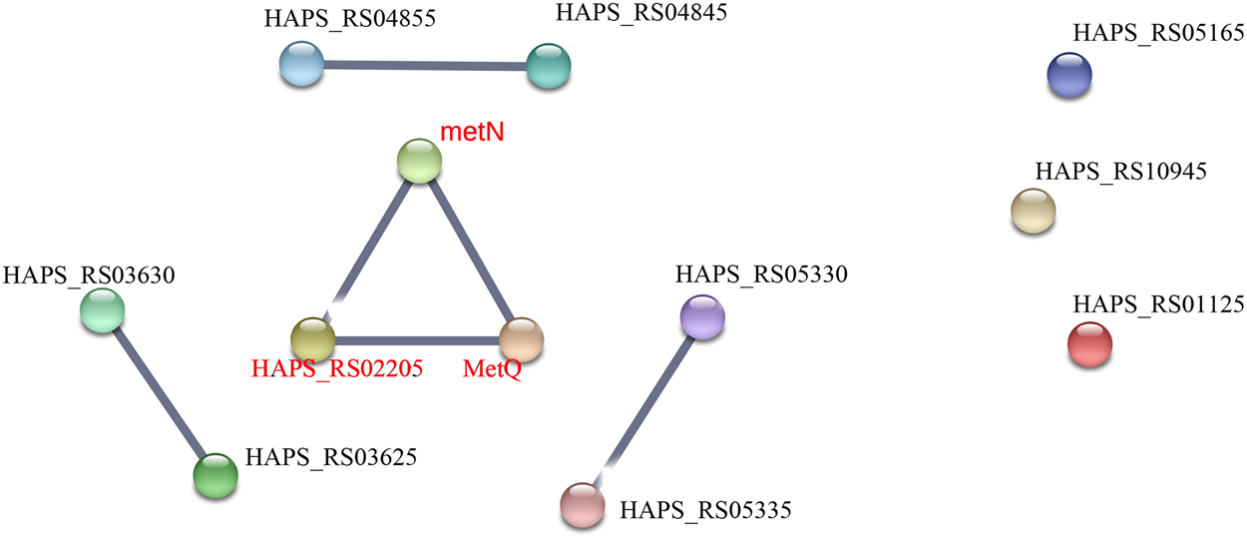
STRING analysis of the relationship between 16 DE genes in ABC transporter. The downregulated genes were marked with red, and the others were upregulated genes.

**Figure 7:**
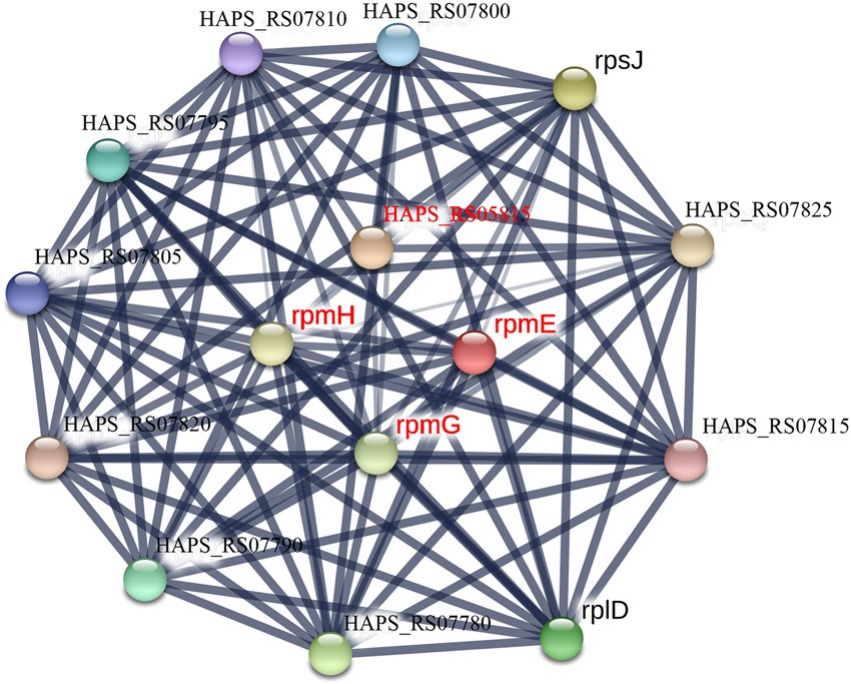
STRING analysis of the relationship between 15 DE genes in ribosome. The downregulated genes were marked with red, and the others were upregulated genes.

STRING is a web-based interface which can predict protein associations – direct physical binding and indirect interaction – such as participation in the same metabolic pathway or cellular process on the basis of genomic context, co-expression and data from reported literature (https://string.embl.de/)^25–27^. DE genes were analysed with STRING to predict the network of proteins encoded by DE genes. DE genes involved in the three main pathways (metabolic pathway, ABC transporters, ribosomes) related to resistance were selected for STRING analysis, using the *Sus scrofa* database. The network of predicted associations for all upregulated and downregulated DE genes encoding proteins and string symbols were shown in Supplementary Files 4–6. The detailed fold changes of major upregulated and downregulated DE genes (FC ≥ 2 or FC ≤ 0.5) of JS32 compared to JS0135 were also shown in Tables 3–4. Among these DE genes, most molecules were key molecules that link to each others, while several encoded proteins which were not linked to each other, indicating that their functions were unrelated or unknown according to the STRING analysis results. As shown in Figs 5–7 (FC ≥ 2 or FC ≤ 0.5), the DE genes of the three main resistance-related pathways encoded proteins which were associated with each other contributing to the resistance of *HPS* to tildipirosin together. The 40 DE genes from the Tables 3 and 4 encoded proteins associated with the metabolic pathway including 30 upregulated and 10 downregulated genes were selected for STRING analysis. Of the 40 genes, 4 DE genes were not found in the STRING database and the other 36 genes were shown in the Fig. 5. Among the 16 DE genes encoded ABC transporter proteins including 11 upregulated and 5 downregulated genes, 4 DE gene were not found in the STRING database, and the network of the other 12 genes were shown in the Fig. 6. The network of 15 DE genes encoded ribosome proteins including 11 upregulated and 4 downregulated genes were shown in the Fig. 7. All of them were linked with each other to regulate the resistance of *HPS* to tildipirosin.

### Validation by real-time quantitative PCR (RT-qPCR)

For verification of the RNA sequencing results, ten of the DE genes and three samples including *JS0135*, *JS32* and *HB32*, were selected on the basis of their importance as resistance determinants. Among the ten tested genes, *HAPS_RS09315*, *HAPS_RS09320*, *HAPS_RS11130*, *HAPS_RS06145*, *glmM*, *HAPS_RS04930*, *HAPS_RS03600*, *HAPS_RS03625*, *HAPS_RS07815* and *HAPS_RS10945* of *JS32* had fold changes of infinity (499,108), infinity (114,954), 1270, 98, 158, 676, 30, 683 and 460, respectively, when their expression levels were compared in the test and reference control. The fold changes of ten *HB32* genes were similar to those of *JS32*.

## Discussion

In the present study, *JS0135* and *HB32* were used to investigate the resistance mechanism to tildipirosin in *HPS*. *JS32* was induced from *JS0135* and could grow well on tryptone soy agar containing 256 MIC tildipirosin. The total bacterial count of *JS32* was significantly (p ≤ 0.01) decreased compared to *JS0135* and *HB32*, but attained logarithmic growth phase faster than the others; the growth curve of *HB32* was similar to *JS0135* (Fig. 1). The results of the current study were similar to those from the research reported by Chunmei Wang^8^. This variation might be associated with tildipirosin stimulation and the DNA replication pathway, which are involved in the downregulation of *rnhB* which expresses ribonuclease HII protein and is essential for growth according to previous reports (Table 4)^28–31^. The growth difference between tildipirosin-resistant and sensitive strains required further research. Three *HPS* serovars were indentified with a previously described multiplex PCR method which is faster, more sensitive and more specific than indirect hemagglutination (IHA)^32^. The results in Supplementary Figure S1 distinctly show that *JS0135*, *JS32* and *HB32* are serovars 4, 4 and 13, respectively.

According to a previous study by Chunmei Wang in 2014, and the significant KEGG membrane transport pathway analysis in Fig. 4, *JS0135*, *JS32* and *HB32* were selected to explore the resistance mechanism by observing membrane morphology diversity with SEM. The SEM results showed that the outer surfaces of induced and wild-type strains *JS32* and *HB32* were smoother than the control (*JS0135*), but no contrast was found between *JS32* and *HB32* (Fig. 2). Similar changes in the ultrastructure of CB-resistant *HPS* have been reported previously^8, 24^. The variance between resistant and sensitive *HPS* might be caused by membrane proteins including those encoded by the upregulated genes *HASP_RS10075*, *HASP_RS11135*, *HASP_RS07320*, *HASP_RS03695*, *HASP_RS08120* and *HASP_RS05335* (Supplementary File 1). The similarity of the *JS32* and *HB32* induced and wild-type tildipirosin-resistant bacteria suggest the same resistance mechanism from the morphology. A known membrane protein gene *HAPS_RS01150* is related to resistance in *Escherichia coli*, encoding an outer membrane lipoprotein involved in copper homeostasis and adhesion; its overproduction was found to increase multidrug resistance and copper through activation of genes encoding the *AcrD* and *Mdt* ABC drug efflux pumps^33, 34^. *HAPS_RS01150* (1.003 fold change) in *JS32* did not show any upregulation in the present study, but other genes encoding proteins displayed up- and downregulation (FC ≥ 2 or FC ≤ 0.5), and it is necessary to study these genes further.

In previous reports, CAMPs were shown to play an important role in inhibiting colonization and clearance of infections; furthermore Gram-negative bacteria represent a major target for CAMPs. However, the development of CAMP resistance permits Gram-negative bacteria to avoid being killed by both the host immune system and antibiotics^35, 36^. CAMP resistance genes including *HAPS_RS07240*, *HAPS_RS11325* and *HAPS_RS06175* encoding relative resistance proteins exhibited upregulation of gene expression (≥twofold changes), shown in Table 3.

The GO and KEGG analysis results are shown in Tables 3, 4; molecular function, biological process, cellular component, integral component of membrane, plasma membrane, transport, transposase activity and DNA-mediated transposition were the most abundant GO classification terms. Metabolic pathways, biosynthesis of secondary metabolites, ribosome, ABC transporters, biosynthesis of antibiotics, purine metabolism, microbial metabolism in diverse environments, quorum sensing and glycerophospholipid metabolism were the most abundant KEGG classification pathways. In these results, increased DE in the treatment group was focused on metabolic pathways, ABC transporters and ribosomes, while decreased DE was focused on metabolic pathways, PTS, ABC transporters and ribosomes (Fig. 4b). These profiles of major upregulated and downregulated genes in GO and KEGG analysis in the Tables 3, 4 have enabled us for the first time to systematically elucidate the resistance of *HPS* to tildipirosin. The following paragraphs analysed the possible mechanisms of *HPS* resistance to tildipirosin from metabolic, PTS, ABC transporters and ribosome pathways.

**Figure 8:**
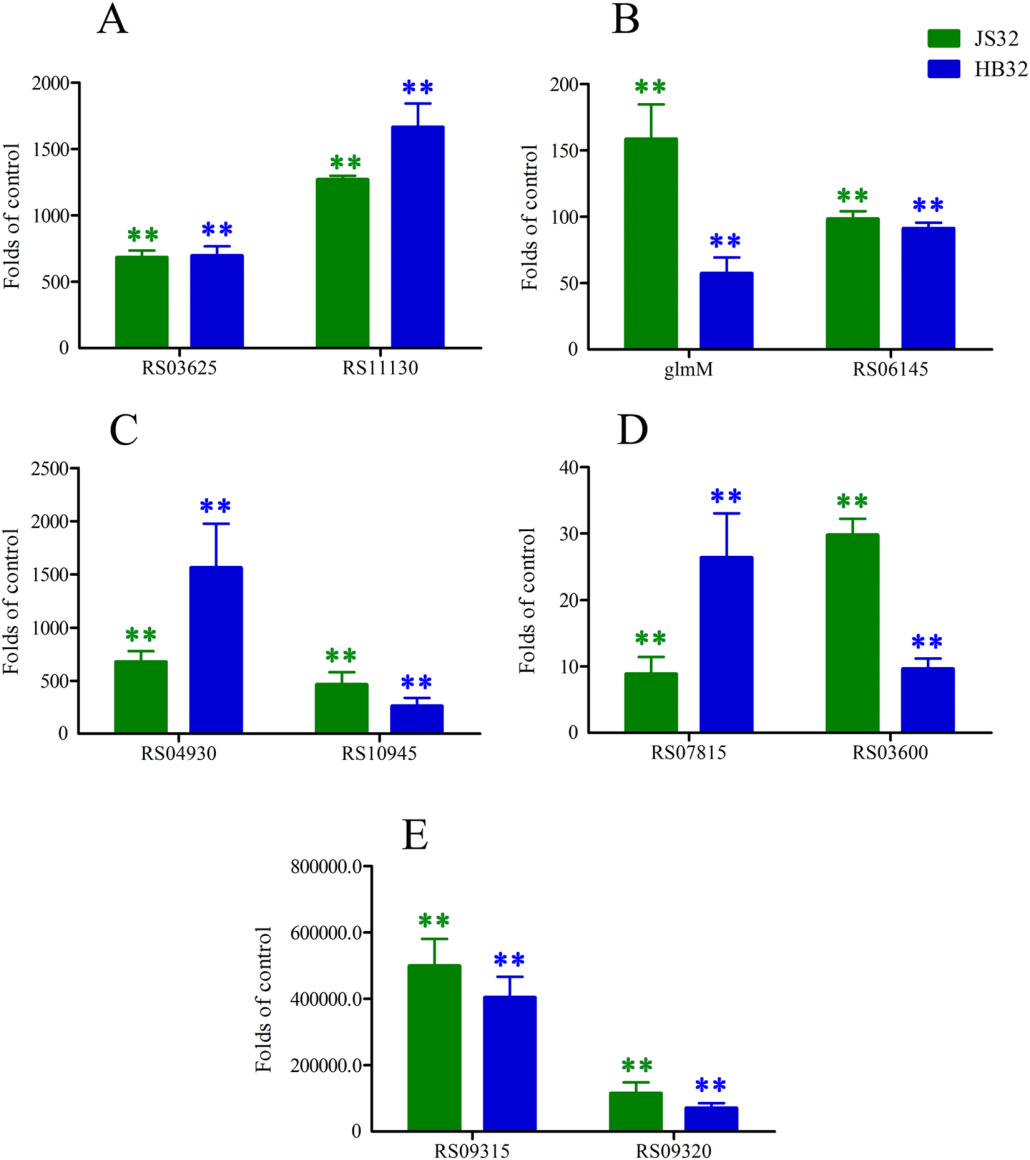
The differential expression on relative mRNA abundance of Ten genes in *JS32* and *HB32* compared with *JS0135*. Control, the value = 1, Values are mean ± SD. *Presents statistically significant *p* ≤ 0.05, **presents extremely significant *p* ≤ 0.01.

The genes involved in metabolic pathways, *HAPS_RS09315*, *HAPS_RS09320*, *HAPS_RS08960*, *HAPS_RS08955* and *HAPS_RS08950*, encoding restriction endonuclease subunit M, DNA cytosine methyltransferase, hydroxyethylthiazole kinase, hydroxymethylpyrimidine kinase and thiamine phosphate synthase, respectively, were infinitely upregulated (Table 3); this was verified by RT-qPCR, which indicates that the RNA sequence results were reliable (Fig. 8). Among these genes, DNA cytosine methyltransferase is a key factor as a marker for the presence of a family of phage-like elements, which confer macrolide resistance in streptococci and resistance to target site methylation in *PM* ^13, 37^. Moreover, nucleotide methylation can also offer antibiotic resistance, such as 16S rRNA methyltransferase in Enterobacteriaceae^38^. It has been previously been reported that the upregulation of thiamine phosphate synthase can cause an increase in resistance to multiple stresses in *Schizosaccharomyces*, and thiamine supplementation might also contribute to chemotherapy resistance in cancer cells^39, 40^. Another key upregulated gene *glmM* (2.6-fold change), encoding phosphoglucosamine mutase, has been demonstrated to contribute to the resistance of *Streptococcus*, and is the drug target for regulating resistance. In addition, *glmM* is directly upstream of a multiple repeat polypeptide essential for the expression of methicillin resistance in *Staphylococcus aureus* ^41–44^. The other upregulated genes have not been reported, but also might contribute to regulate metabolic pathways related to bacterial resistance to tildipirosin which were in need of verification in the future.

Other upregulated ribosome and ABC transporter pathway genes encoding ribosomal proteins, transporter permeases and membrane proteins, including *HAPS_RS07815*, *HAPS_RS07810*, *rpsJ*, *rplD*, *HAPS_RS07825*, *HAPS_RS07790*, *HAPS_RS07805* and *HAPS_RS07780*; and *HAPS_RS10945*, *HAPS_RS03625*, *HAPS_RS05335*, *HAPS_RS03630*, *HAPS_RS00310*, *HAPS_RS05165* and *HAPS_RS00315*, respectively, were found to have a significant effect on the treatment group. Previous reports have stated that tigecycline resistance is associated with mutations in *rpsJ* in *Klebsiella pneumoniae*. *RpsJ* acts as general target of tigecycline adaption and a marker for alterations in antibiotic resistance in bacteria; the V57L mutation in *rpsJ* might cause weaker binding of tigecycline to 16S rRNA, leading to tigecycline resistance^45–48^. In *RplD*, encoding the ribosomal protein L4, it has also been found that the A2059G mutation confers resistance to macrolides and lincosamides^12, 49–51^. Other genes relative to ribosomes, encoding ribosomal proteins, are also concerned with resistance. Dennis conducted a study on the *E. coli* response to chloramphenicol^52^; when Gram-negative bacteria, such as *HPS*, experience low levels of translation inhibition, a compensatory mechanism might be triggered in which the synthesis of ribosomal proteins is initially upregulated, but as the inhibition stress increases this compensation fails to keep pace and the cells succumb to antibiotic killing^20, 52^. There were 11 significantly upregulated genes related to the ABC transport system in the treatment group (Table 3). The bacterial cell envelope is a target of many antibiotics, and disruption of its structure inhibits transmembrane transport functions and impairs normal physiological functions. The key transport systems critical for bacterial viability and survival are the ABC transporter pathways^53^. ABC transporters play a significant role in bacteria, conferring multidrug resistance (MDR) through overexpression as described in previous reports^54^. Moreover, the active movement of compounds across membranes carried out by ABC transporters can cause drug resistance in anti-infective therapies^55^. Resistance against antimicrobial peptides in many firmicutes bacteria is mediated by an ABC transporter^56^. ABC transporters are involved in secretion of the antibiotic through the cell membrane and also contribute to acquisition of antibiotic resistance. ABC transporters were the first proteins to be implicated in the mechanism of resistance to macrolides, as described in antibiotic-producing actinomycetes^57, 58^. The variation between treatment and control groups was also caused by the ABC transporter cell membrane proteins expressed, as described in Fig. 6. Although DE genes in the ABC transporter pathway have not been reported in resistance, these are novel genes related to the resistance mechanism, worth exploring further.

Other downregulated genes belonging to the PTS, metabolism, ribosome and ABC transport pathways are shown in Table 4. The PTS system is responsible for the transport of a variety of carbohydrates in prokaryotes. PTS components participate in signal transduction, chemotaxis and the regulation of essential physiological processes^59, 60^. As for downregulation, reduced expression of ABC transporter genes (ABC subfamily) is tightly linked to Cry1Ac resistance in *Plutella xylostella* ^61^. All downregulated genes in these pathways, such as *metQ*, *MetN*, *metF* and *rpmE*, contributed to the regulation of resistance to tildipirosin in this study, shown in Table 4. Meanwhile, the STRING analysis indicated that the main up and down-regulated DE genes encoded proteins which could interact with the metabolic pathway (Fig. 5), ABC transporters (Fig. 6), ribosomes (Fig. 7) and PTS, regulating these genes or other cells to facilitate the resistance of tildipirosin in *HPS*. *HAPS_RS08950*, *HAPS_RS08955* and *HAPS_RS08960* which were part of metabolic pathway encoded thiamine phosphate synthase, hydroxymethylpyrimidine and hydroxyethylthiazole kinase, respectively were associated with each other immediately whose upregulated fold changes were infinite in the Fig. 5 and Table 3. Meanwhile, downregulated genes of metN, HAPS_RS02205 and MetQ belonging to ABC transporters pathway in the Fig. 6 were linked with each other, and all up and down regulated genes of ribosome pathway were connected with each other closely in the Fig. 7. All of these key genes regulated and controlled the resistance of *HPS* to tildipirosin together, especially for the upregulated genes of metabolic pathway who may contributed to resistance of *HPS* crucially.

Ten selected genes from the transcriptome profiling in Table 3 were selected for RNA sequence validation by RT-qPCR. There was the same trend of upregulation, but a difference in fold changes in these genes between transcriptome and RT-qPCR analysis, shown in Fig. 8. The main reasons were different batches of samples resulting in fold change variation.

Concluding our findings, the data obtained from transcriptional profiling of *JS32* and *JS0135* provide new sights into the complex mechanisms underlying the general response to tildipirosin treatment. In addition, distinctive DE genes in the treatment group indicate that more attention should be paid to a new resistance factor metabolic pathway, particularly related to the upregulated genes (*HAPS_RS09315*, *HAPS_RS09320*, *HAPS_RS08950* and *HAPS_RS08955*) which are overexpressed infinitely. The other new genes *HAPS_RS03625* and *HAPS_RS04930* (fold changes > 500, Fig. 8) involved in ribosomes, ABC transport and CAMP, which are interrelated closely as shown in Figs 5–7, are also worthy of future study. The new tildipirosin resistance mechanisms in *HPS* are complex, and this study provides a new perspective to study macrolide resistance. More attention to study at the protein level is needed to investigate the expression of resistance genes.

## Materials and Methods

### Bacterial strains and antibiotics

*HPS JS0135* was obtained from the State Key Laboratory of Microbiology at Huazhong Agricultural University; *HB32* was isolated from the lung of a diseased piglet in Jiangsu and Hubei, China. They were identified as serovars 4 and 13, respectively, by PCR with a previously described method^32, 62^. The primers were designed as shown in Table 1. *HPS* was subcultured in tryptone soya agar (TSA) and tryptone soya broth (TSB) (Qingdao Hai Bo Biological Technology Co., Ltd., Shangdong, China) supplemented with 5% fetal bovine serum (Zhejiang Tianhang Biotechnology Co., Ltd., Zhejiang, China) and 10 μg/ml nicotinamide adenine dinucleotide (NAD) (Qingdao Hope Bio-Technology Co., Ltd., Shandong, China). Tildipirosin with >99.5% purity was used, donated from Hubei Huisheng Biological Technology Company (Hubei, China).

### Determination of induced and natural resistance

The MICs of *JS0135*, *JS32* and *HB32* were determined with twofold broth dilution (0.0625–32 μg/ml) according to the CLSI M07-A9 standard. *Enterococcus faecalis* (ATCC 29212) was used as the quality control (QC) strain to detect the credibility of susceptibility testing^63^. *JS32* was induced from *JS0135* by incubation with increasing tildipirosin concentrations (from 0.0625 to 64 μg/ml)^8^. One colony of *JS0135* (MIC = 0.125) was incubated into TSB with 0.5 MIC tildipirosin at 37 °C with shaking (220 rpm) for 12 h. When induced colonies had grown stable, cultures were inoculated into TSB with the next highest concentration of tildipirosin^64^. At last, one colony (MIC = 32) remained with high resistance stability, and was named *JS32*. *HB32* (MIC = 32), a clinical isolate, is a naturally resistant strain. MICs for tildipirosin to *HPS* were determined by using agar dilution method as recommended by the Clinical and Laboratory Standards Institute (CLSI) M31-A3 guidelines. All experiments involved in MIC determination were preformed according to these guidelines.

### Growth curve comparison

*JS32*, *HB32* and *JS0135* were inoculated into TSB cultures for more than three generations until stable growth was achieved. Then, 100 μl of the three bacterial cultures (1 × 10^6^ CFU/ml) was selected to inoculate into new 100 ml TSB cultures. Each newly selected strain was incubated on a shaker at 220 rpm at 37 °C for 24 h. Growth curves were determined by measuring the optical density (600 nm) of the cultures every 2 h with a spectrophotometer (UV2100, Shanghai, China).

### Transmission Electron Microscopy analysis

Bacteria (*JS32*, *JS0135*, *HB32*) were cultured in TSB to reach mid-logarithmic phase (12 h). Three cultures were centrifuged and washed with phosphate-buffered saline (PBS) twice. The washed bacterial sediment was fixed with 2.5% buffered glutaraldehyde for 1 h, and then fixed in 1% buffered osmium tetroxide for 1 h. The fixed samples were dehydrated through a graded ethanol series, and embedded in resin. The morphology of *JS32*, *JS0135* and *HB32* was observed using a Tecnai G2 20 S-TWIN transmission electron microscopy (TEM) (JSM-6390LV, NTC, Japan) at an acceleration voltage of 200 kv (FEI, Hillshoro, Oreg, USA).

### Transcriptome analysis

In this study, an RNA sequence analysis was prepared and submitted to Shanghai Biochip Corporation (Shanghai, China) for mRNA purification, library preparation and sequencing. In brief, bacterial cultures (*JS32*, *JS0135*, *HB32*) were centrifuged for 10 min (3000 *g* at 4 °C). Total RNA of bacterial samples was extracted and purified with RNAiso Plus Reagent (TaKaRa Biotechnology Co., Ltd, USA) and DNase (Qiagen, Germany) according to the manufacturer’s instructions^20^. The remaining DNA was removed by RNase-free DNase I (Ambion Inc., Texas, USA). RNA concentration and purity were evaluated by A260/A280 spectrophotometer readings (NanoDrop 2000, Thermo Fisher Scientific Inc., USA) and agarose gel electrophoresis, respectively. Ribosomal RNA was removed from the total RNA with Ribozero Kit was followed with the strand specific RNA-seq protocol on Illumina Hiseq. 2500 platform (paired-edn sequencing; 100 bp fragments) at Shanghai Biochip Corporation. Firstly, strand cDNA synthesis was conducted with using SuperScriptII (Invitrogen, Carlsbad, CA) in the presence of random hexamer primers. Secondly, another cDNA was synthesized before end-repair and dA-tailing. DNA fragment ligation was performed with TruSeq adapter and amplified with TruSeq PCR primers for sequencing. Reads longer than 35 nt and ≤2 N (ambiguous nucleotides) were retained. Meanwhile, paired reads that got mapped to sliva database (https://www.arb-silva.de/download/arb-files/) were removed.

Each gene expression in different samples were transformed to counts per gene (CPG) by DE sequence package with blind and fit-only parameter^65^. Mean and SD of CPG expression were calculated for JS32 and JS0135 from their respective repeats and compared to check the DE genes. Genes with a fold change ≥2 and q-value ≤ 0.05 were selected for analysis, since a 1.5-fold change in transcription level was regarded as biologically significant in previous studies^66, 67^. DE analysis of the transcripts was conducted with the R package DESEq^68^. A transcript was considered to have significant DE if the false discovery rate (FDR) was ≤0.05. The data had been deposited in Gene Expression Omnibus (GEO) and were accessible through accession number GSE42814 (https://www.ncbi.nlm.nih.gov/gds/?term=SH0165). GO, as an international standardized system for a functional classification of genes, provided an updated terminology and comprehensively described the properties of genes and their products in the organism. KEGG database (https://www.genome.jp/kegg) was utilized to find the linkage of the DE with different pathways. Functional classification of transcripts with significant DE was conducted with Blast2GO software and KEGG pathway analysis. Associations of the proteins encoded by DE genes were analysed with STRING (http://www.string-db.org/)^25^.

### RT-qPCR analysis

Ten genes (*HAPS_RS03625*, *HAPS_RS11130*, *glmM*, *HAPS_RS06145*, *HAPS_RS04930*, *HAPS_RS10945*, *HAPS_RS07815*, *HAPS_RS03600*, *HAPS_RS09315*, *HAPS_RS09320*) encoding proteins related to the resistance mechanism of *HPS* were selected for validation of RNA sequence results with RT-qPCR (CFX 384, Bio-Rad). Total RNA was extracted from *JS32*, *JS0135* and *HB32*. RT-qPCR was performed in triplicate as described previously^65, 69^. All primers were originally designed by the NCBI online primer-blast function, as shown in Table 1 (https://www.ncbi.nlm.nih.gov/). The thermal cycler conditions were as follows: denaturation at 95 °C for 10 s, annealing at 56 °C for 20 s and extension at 72 °C for 20 s. The 2^−ΔΔCt^ method was used for quantification with 16S rRNA as a reference gene, and the relative abundance was normalized to the control. The fold changes were calculated by the 2^−ΔΔCt^ formula^70^.

### Statistical analysis

Statistical analysis were conducted with using SPSS version 22.0 (IBM Corp., Armonk, NY, USA). The two-tailed t-test was applied to estimate the mean ± standard deviation (SD) and significant difference of RNA-seq and RT-qPCR results. A p value of ≤0.05 was considered to indicate a statically significant result. *p ≤ 0.05 and **p ≤ 0.01.

### Ethic Statement

The animals which were used to isolate *HPS* in this study were conducted according to relevant guidelines and regulations of Animal Care Center, Hubei Science and Technology Agency in China (SYXK 2013-0044) and animal housing care and experimental protocol were conducted according to the regulation of experimental animal usage in Hubei province of China. In addition, the protocol was approved by the Ethics Committee of Huazhong Agricultural University.

## Electronic supplementary material

Supplementary file 1

Supplementary file 2

Supplementary file 3

Supplementary file 4

Supplementary file 5

Supplementary file 6

Figure. S1

Figure. S2
