# Supplementary material for: Comparative transcriptional profiling of tildipirosin-resistant and sensitive Haemophilus parasuis
Source: Sci Rep. 2017 Aug 8;7:7517. doi: 10.1038/s41598-017-07972-5 (PMC5548900; doi:10.1038/s41598-017-07972-5)
Supplement: Supplementary file 1 [file 41598_2017_7972_MOESM1_ESM.pdf]

Comparative transcriptional profiling of tildipirosin-resistant and sensitive *Haemophilus parasuis*

Zhixin Lei[ab], Shulin Fu[c], Bing Yang[ab], Qianying Liu[ab], Saeed Ahmed[ab], Lei Xu[c], Jincheng Xiong[ab], Jiyue Cao[ab*], Yinsheng Qiu[c*]

[a] Veterinary Pharmacology Laboratory, College of Veterinary Medicine, Huazhong Agricultural University, Wuhan, 430070, PR China

[b] National Reference Laboratory of Veterinary Drug Residues and MAO Key Laboratory for Detection of Veterinary Drug Residues, Huazhong Agriculture University, Wuhan, 430070, PR China

[c] School of Animal Science and Nutritional Engineering, Wuhan Polytechnic University, Wuhan 430023, PR China

[*]***Corresponding author:***

Prof. Dr. Ji-yue Cao, Caojiyue@mail.hzau.edu.cn

Prof. Dr. Yinsheng Qiu, qiuyinsheng6405@aliyun.com

Table. 1 The total upregulated and downregulated DE genes of JS32 compared with JS0135

gene id	gene name	description	GO_term	kegg_term	log2FC	updown
7278765	HAPS_RS09320	DNA cytosine methyltransferase	DNA (cytosine-5-)-methyltransferase activity|DNA modification	Metabolic pathways|Cysteine and methionine metabolism|MicroRNAs in cancer	Inf	UP
7276735	HAPS_RS02885	hypothetical protein	-	-	Inf	UP
23375380	HAPS_RS03985	transposase, partial	-	-	Inf	UP
7278764	HAPS_RS09315	restriction endonuclease subunit M	DNA (cytosine-5-)-methyltransferase activity|DNA modification	Metabolic pathways|Cysteine and methionine metabolism|MicroRNAs in cancer	Inf	UP
7276852	HAPS_RS00230	spore coat protein	response to antibiotic|lipid A biosynthetic process|transaminase activity|pyridoxal phosphate binding	-	Inf	UP
7278673	HAPS_RS08895	site-specific DNA-methyltransferase	DNA-methyltransferase activity|DNA methylation	-	Inf	UP
7278672	HAPS_RS08890	type III restriction-modification system EcoPI enzyme subunit res	DNA restriction-modification system|restriction endodeoxyribonuclease activity	-	Inf	UP
7278763	HAPS_RS09310	type II restriction endonuclease HgAI	-	-	Inf	UP
7276974	HAPS_RS02055	chromosome segregation ATPase	molecular_function|biological_process|cellular_component	-	Inf	UP
7278766	HAPS_RS09325	transcriptional regulator	cellular_component|biological_process|molecular_function	-	Inf	UP
7278680	HAPS_RS08930	DUF305 domain-containing protein	-	-	Inf	UP
7276848	HAPS_RS00210	CMP-N-acetylneuraminate-beta-galactosamide-alpha-2, 3-sialyltransferase	-	-	Inf	UP
7278297	HAPS_RS03810	hypothetical protein	-	-	Inf	UP
7276850	HAPS_RS00220	glycosyl transferase	molecular_function|biological_process|cellular_component	-	Inf	UP
7278682	HAPS_RS08940	methionine sulfoxide reductase	-	-	Inf	UP

7276849	HAPS_RS00215	hypothetical protein	-	-	Inf	UP
7278338	HAPS_RS04015	preprotein translocase	DNA recombination|protein binding|establishment of integrated proviral latency|provirus excision|DNA integration|provirus|prophage integrase activity|viral entry into host cell|DNA binding	-	Inf	UP
7276734	HAPS_RS02880	hypothetical protein	-	-	5.838693486	UP
7276851	HAPS_RS00225	glycosyl transferase	cytoplasm|fucosylation|fucosyltransferase activity|transferase activity|protein glycosylation|transferase activity, transferring glycosyl groups|galactoside 2-alpha-L-fucosyltransferase activity|glycoprotein-N-acetylgalactosamine 3-beta-galactosyltransferase activity|alpha-(1,2)-fucosyltransferase activity	-	Inf	UP
7278208	HAPS_RS10560	transcriptional regulator	biological_process|molecular_function|cellular_component	-	9.704690558	UP
7276854	HAPS_RS00240	polysaccharide biosynthesis protein CapD	catalytic activity|polysaccharide biosynthetic process	-	Inf	UP
7278750	HAPS_RS09250	transcriptional regulator	-	-	Inf	UP
23375326	HAPS_RS01830	hypothetical protein	regulation of transcription, DNA-templated|sequence-specific DNA binding transcription factor activity	-	6.454452277	UP
7278674	HAPS_RS08900	ATPase (AAA+ superfamily) protein	-	-	Inf	UP
7276853	HAPS_RS00235	glycosyl transferase	molecular_function|biological_process	-	Inf	UP
7278337	HAPS_RS04010	transcriptional regulator	-	-	Inf	UP
7277433	HAPS_RS01225	hypothetical protein	biological_process|molecular_function|cellular_component	-	Inf	UP
7276929	HAPS_RS01835	hypothetical protein	cellular_component|biological_process|molecular_function	-	5.110947532	UP
7278671	HAPS_RS08885	transcriptional regulator	sequence-specific DNA binding transcription factor activity|regulation of transcription, DNA-templated	-	Inf	UP

7278678	HAPS_RS08920	transcriptional regulator	sequence-specific DNA binding transcription factor activity|regulation of transcription, DNA-templated	-	Inf	UP
23375379	HAPS_RS03980	MULTISPECIES: hypothetical protein, partial	-	-	Inf	UP
7278676	HAPS_RS08910	MerR family transcriptional regulator	regulation of transcription, DNA-templated	-	Inf	UP
23375304	HAPS_RS00740	hypothetical protein	-	-	4.186471477	UP
7278035	HAPS_RS00970	PTS mannose transporter subunit IIAB	mannose transport|phosphoenolpyruvate-dependent sugar phosphotransferase system|protein-N(PI)-phosphohistidine-sugar phosphotransferase activity|carbohydrate transmembrane transport	Metabolic pathways|Amino sugar and nucleotide sugar metabolism|Fructose and mannose metabolism|Phosphotransferase system (PTS)	-2.599575471	DOWN
7278762	HAPS_RS09305	DDE transposase	transposase activity|transposition, DNA-mediated	-	Inf	UP
7277472	-	-	-	-	-3.721064565	DOWN
7277936	HAPS_RS07445	3D-(3,5/4)-trihydroxycyclohexane-1,2-dione acylhydrolase (decyclizing)	transferase activity|inositol catabolic process|hydrolase activity, acting on acid carbon-carbon bonds, in ketonic substances	Metabolic pathways|Microbial metabolism in diverse environments|Inositol phosphate metabolism	4.303853938	UP
7278335	HAPS_RS04000	hypothetical protein	-	-	Inf	UP
23375341	HAPS_RS02410	hypothetical protein	anaerobic respiration|iron ion binding|4 iron, 4 sulfur cluster binding|metal ion binding|dimethyl sulfoxide reductase complex|iron-sulfur cluster binding	Sulfur metabolism	Inf	UP
7278810	-	-	-	-	-3.665528435	DOWN
7278033	HAPS_RS00960	PTS fructose transporter subunit IID	protein-N(PI)-phosphohistidine-mannose phosphotransferase system transporter activity|hexose transmembrane transport|plasma membrane|carbohydrate transport|monosaccharide transport|metabolic process|mannose transport|membrane|transmembrane	Metabolic pathways|Amino sugar and nucleotide sugar metabolism|Fructose and mannose metabolism|Phosphotransferase system (PTS)	-2.630006793	DOWN

			transport|phosphoenolpyruvate-dependent sugar phosphotransferase system|integral component of membrane|carbohydrate transmembrane transport|integral component of plasma membrane|transport			
7278034	HAPS_RS00965	PTS fructose transporter subunit IIC	membrane|plasma membrane|carbohydrate transport|transport|integral component of membrane|phosphoenolpyruvate-dependent sugar phosphotransferase system	Metabolic pathways|Amino sugar and nucleotide sugar metabolism|Fructose and mannose metabolism|Phosphotransferase system (PTS)	-2.33608009	DOWN
23375305	HAPS_RS00745	hypothetical protein	-	Pertussis	3.191135334	UP
7278683	HAPS_RS08945	MULTISPECIES: hypothetical protein	cellular_component|biological_process|molecular_function	-	Inf	UP
7278037	HAPS_RS00980	DeoR family transcriptional regulator	sequence-specific DNA binding transcription factor activity|transcription, DNA-templated|intracellular|DNA binding|regulation of transcription, DNA-templated	-	-2.399428365	DOWN
7278125	-	-	-	-	-4.284654209	DOWN
7277583	HAPS_RS08105	transposase	cellular_component|molecular_function|biological_process	-	Inf	UP
7278871	HAPS_RS05575	integrase	establishment of integrated proviral latency|prophage integrase activity|viral entry into host cell|DNA binding|DNA integration|DNA recombination	-	2.929602194	UP
7277222	HAPS_RS04905	PTS sucrose transporter subunit IIBC	sucrose transport|phosphoenolpyruvate-dependent sugar phosphotransferase system	Phosphotransferase system (PTS)|Starch and sucrose metabolism	-2.552407786	DOWN
7277414	HAPS_RS01140	sugar ABC transporter substrate-binding protein	-	-	-1.849192963	DOWN
7277937	HAPS_RS07450	myo-inosose-2 dehydratase	myo-inosose-2 dehydratase activity|inositol catabolic process	Metabolic pathways|Microbial metabolism in diverse environments|Inositol phosphate	2.675045058	UP

				metabolism		
7277669	rnhB	ribonuclease HII	RNA binding|RNA-DNA hybrid ribonuclease activity|manganese ion binding|nuclease activity|nucleic acid binding|hydrolase activity|RNA phosphodiester bond hydrolysis, endonucleolytic|nucleic acid phosphodiester bond hydrolysis|metal ion binding|endonuclease activity|RNA catabolic process|RNA phosphodiester bond hydrolysis|cytoplasm	DNA replication	-1.832547092	DOWN
7276695	HAPS_RS02700	hypothetical protein	-	-	Inf	UP
23375440	HAPS_RS07230	hypothetical protein	-	-	Inf	UP
7277084	HAPS_RS04945	transcriptional regulator IlvY	cytoplasm|branched-chain amino acid biosynthetic process|regulation of transcription, DNA-templated|DNA binding|cellular amino acid biosynthetic process|transcription, DNA-templated|sequence-specific DNA binding transcription factor activity	-	2.289202683	UP
7278811	-	-	-	-	-2.924620532	DOWN
7278036	HAPS_RS00975	tagatose-6-phosphate ketose isomerase	carbohydrate binding|intramolecular oxidoreductase activity, interconverting aldoses and ketoses|carbohydrate metabolic process|isomerase activity	-	-1.772358261	DOWN
23375314	HAPS_RS01405	hypothetical protein	-	-	-2.053383027	DOWN
7278453	metN	D-methionine ABC transporter, ATP-binding protein	ATP catabolic process|Gram-negative-bacterium-type cell wall|amino acid transmembrane transport|protein binding|ion transmembrane transport|hydrolase activity|D-methionine transport|nucleotide binding|amino acid transport|nucleoside-triphosphatase activity|methionine transport|D-methionine transmembrane	ABC transporters	-1.506498993	DOWN

			transporter activity|ATPase activity|plasma membrane|metabolic process|methionine transmembrane transporter activity|membrane|transport|transmembrane transport|ATP binding			
7277452	HAPS_RS01315	NAD(P)H-dependent oxidoreductase	electron carrier activity	-	-1.694393168	DOWN
7277495	HAPS_RS06575	DDE transposase	transposition, DNA-mediated|transposase activity	-	-2.781873899	DOWN
7278379	HAPS_RS07615	colicin Js receptor	colicin transport|receptor activity|colicin transmembrane transporter activity	-	Inf	UP
7278424	HAPS_RS07815	MULTISPECIES: 50S ribosomal protein L16	structural constituent of ribosome|intracellular|translation|cytosolic large ribosomal subunit|ribosome|tRNA binding|rRNA binding|ribonucleoprotein complex|RNA binding	Ribosome	2.157934836	UP
7278753	HAPS_RS09265	DNA uptake Rossmann fold nucleotide-binding protein	molecular_function	-	Inf	UP
7277899	HAPS_RS07265	biotin transporter BioY	molecular_function|biological_process	ABC transporters	-1.698839064	DOWN
7277685	HAPS_RS08615	MULTISPECIES: integrase, partial	transposition, DNA-mediated|protein-DNA complex|transposase activity|sequence-specific DNA binding|DNA recombination|nucleic acid binding|DNA binding|transposition|DNA insertion or deletion binding|DNA integration	-	5.644000208	UP
7278469	HAPS_RS02290	carbon storage regulator	RNA binding|regulation of carbohydrate metabolic process	Two-component system	-1.800127114	DOWN
7278922	HAPS_RS05820	hypothetical protein	-	-	-1.77171914	DOWN
7278438	HAPS_RS07890	F0F1 ATP synthase subunit epsilon	proton-transporting ATP synthase complex, catalytic core F(1)|proton-transporting ATP synthase activity, rotational mechanism|plasma membrane ATP synthesis coupled proton transport|ATP catabolic process|transport|ATP synthesis coupled proton transport|ion	Metabolic pathways|Oxidative phosphorylation|Photosynthesis	-2.984968884	DOWN

			transport|proton-transporting ATPase activity, rotational mechanism|ATP binding|ATP biosynthetic process|proton transport|membrane|plasma membrane			
7278681	HAPS_RS08935	diguanylate cyclase	fatty acid biosynthetic process|enoyl-[acyl-carrier-protein] reductase (NADH) activity	Metabolic pathways|Fatty acid metabolism|Fatty acid biosynthesis	Inf	UP
7278246	HAPS_RS03560	hypothetical protein	-	-	-1.920244436	DOWN
7276766	HAPS_RS03030	hypothetical protein	-	-	Inf	UP
7278032	HAPS_RS00955	beta-galactosidase	carbohydrate metabolic process|beta-galactosidase activity	Galactose metabolism	-1.40626703	DOWN
25120000	-	-	cellular_component|molecular_function|biological_process	-	Inf	UP
7278840	HAPS_RS05430	ABC transporter	membrane|transport|ATPase activity, coupled to transmembrane movement of substances|ATP binding	Quorum sensing	2.062121604	UP
7277476	-	-	-	-	-3.343406487	DOWN
7278334	HAPS_RS03995	hypothetical protein	-	-	Inf	UP
7276680	HAPS_RS02625	hypothetical protein	-	-	2.106597431	UP
23375386	HAPS_RS04250	transcriptional regulator	biological_process|molecular_function|cellular_component	-	1.971845738	UP
25119994	HAPS_RS11185	restriction endonuclease subunit S	-	-	2.072210552	UP
7277560	HAPS_RS07995	nucleotide exchange factor GrpE	protein folding|adenyl-nucleotide exchange factor activity|response to stress	-	-1.66978547	DOWN
7278533	HAPS_RS02610	hypothetical protein	cytochrome complex assembly|oxidation-reduction process|catalytic activity	-	1.920624235	UP
7278819	HAPS_RS05315	MULTISPECIES: translation initiation factor IF-1	translational initiation|translation initiation factor activity	-	-2.201028312	DOWN
7278971	HAPS_RS06055	sugar fermentation stimulation protein SfsA	DNA binding|positive regulation of transcription, DNA-templated	-	1.89514219	UP
7278841	HAPS_RS05435	ABC transporter permease	integral component of plasma membrane|peptide-transporting ATPase activity|peptide	Quorum sensing	1.952413852	UP

			transport|ATP-binding cassette (ABC) transporter complex			
23375560	HAPS_RS10725	polysaccharide biosynthesis protein	-	-	6.598409601	UP
7277209	HAPS_RS07115	hypothetical protein	cellular_component|molecular_function|biological_process	-	-1.38310727	DOWN
7277241	metF	5,10-methylenetetrahydrofolate reductase	methionine metabolic process|methionine biosynthetic process|oxidoreductase activity|cellular amino acid biosynthetic process|cytosol|oxidation-reduction process|methylenetetrahydrofolate reductase (NAD(P)H) activity|tetrahydrofolate interconversion	Metabolic pathways|Microbial metabolism in diverse environments|Carbon metabolism|Carbon fixation pathways in prokaryotes|One carbon pool by folate	-1.296638212	DOWN
7278422	HAPS_RS07805	MULTISPECIES: 50S ribosomal protein L22	ribonucleoprotein complex|rRNA binding|response to antibiotic|large ribosomal subunit|RNA binding|cytosolic large ribosomal subunit|translation|intracellular|structural constituent of ribosome|ribosome	Ribosome	1.893095211	UP
7278423	HAPS_RS07810	30S ribosomal protein S3	ribosome|cytosolic small ribosomal subunit|structural constituent of ribosome|translation|intracellular|mRNA binding|small ribosomal subunit|RNA binding|ribonucleoprotein complex|rRNA binding	Ribosome	1.927744951	UP
7277502	HAPS_RS06610	cell envelope protein TonB	-	-	2.123051769	UP
23375342	HAPS_RS02415	hypothetical protein	membrane|plasma membrane|anaerobic respiration|integral component of membrane|dimethyl sulfoxide reductase complex|oxidoreductase activity|anaerobic electron transport chain|oxidation-reduction process	-	4.130966859	UP
7277561	HAPS_RS08000	hypothetical protein	-	-	-1.842362909	DOWN
7277442	HAPS_RS01265	ABC transporter ATP-binding protein	anion transmembrane transporter activity|anion transport|ATP binding|ATPase activity, coupled to transmembrane movement of substances	-	-1.964921991	DOWN

25119992	HAPS_RS11175	hypothetical protein	collagen trimer|extracellular matrix structural constituent	-	3.363081732	UP
25120023	HAPS_RS11330	hypothetical protein	collagen trimer|metal ion binding|extracellular matrix structural constituent	-	-1.460771024	DOWN
7278663	HAPS_RS08850	preprotein translocase subunit SecE	protein secretion	Quorum sensing|Protein export|Bacterial secretion system	-1.396834435	DOWN
7278356	HAPS_RS04105	tRNA 5-methoxyuridine(34)/uridine 5-oxyacetic acid(34) synthase CmoB	transferase activity, transferring alkyl or aryl (other than methyl) groups|transferase activity|methylation|tRNA (uracil) methyltransferase activity|tRNA methylation|tRNA wobble uridine modification|methyltransferase activity	-	-1.554116427	DOWN
7278347	-	-	-	-	-2.26124672	DOWN
7278419	HAPS_RS07790	MULTISPECIES: 50S ribosomal protein L23	structural constituent of ribosome|translation	Ribosome	1.780584045	UP
7278505	HAPS_RS02470	S-adenosylmethionine synthase	methionine adenosyltransferase activity|magnesium ion binding|ATP binding|potassium ion binding	Metabolic pathways|Biosynthesis of secondary metabolites|Biosynthesis of amino acids|Cysteine and methionine metabolism	-1.262569701	DOWN
7277659	-	-	-	-	-2.61267293	DOWN
25120015	-	-	transposase activity|transposition, DNA-mediated	-	-3.672924454	DOWN
7277082	HAPS_RS04935	ATP-dependent helicase	helicase activity|hydrolase activity|ATP binding|nucleic acid binding|DNA binding|nucleotide binding|ATP-dependent helicase activity|ATP catabolic process|ATP-dependent DNA helicase activity|DNA duplex unwinding|hydrolase activity, acting on acid anhydrides	-	1.758325282	UP
7278418	rplD	50S ribosomal protein L4	translation|cytosolic large ribosomal subunit|regulation of transcription, DNA-templated|regulation of	Ribosome	1.875752748	UP

			translation|structural constituent of ribosome|negative regulation of translation|ribosome|DNA-templated transcription, termination|rRNA binding|ribonucleoprotein complex|response to antibiotic|RNA binding|translation repressor activity|transcription, DNA-templated			
7277266	HAPS_RS02615	cytochrome C biogenesis protein CcdA	molecular_function|cytochrome complex assembly|membrane	-	1.944093839	UP
7277492	HAPS_RS06560	5-methyltetrahydropteroyltriglutamate--homocysteine S-methyltransferase	5-methyltetrahydropteroyltriglutamate-homocysteine S-methyltransferase activity|methionine biosynthetic process	Metabolic pathways|Biosynthesis of secondary metabolites|Biosynthesis of amino acids|Cysteine and methionine metabolism|Selenocompound metabolism	-1.509862246	DOWN
23375557	-	-	-	-	7.306123217	UP
7276679	HAPS_RS02620	peptide-methionine (R)-S-oxide reductase	pathogenesis|peptide-methionine (S)-S-oxide reductase activity	-	1.775356644	UP
7277864	HAPS_RS04820	hypothetical protein	-	-	-2.937405903	DOWN
7277443	HAPS_RS01270	dehydrogenase	cellular_component|biological_process|molecular_function	-	-2.155789631	DOWN
7276916	HAPS_RS00550	division/cell wall cluster transcriptional repressor MraZ	biological_process|molecular_function|cellular_component	-	-1.301021645	DOWN
7278189	HAPS_RS10065	C4-dicarboxylate ABC transporter	C4-dicarboxylate transmembrane transporter activity|C4-dicarboxylate transport	Two-component system	-1.123513198	DOWN
7278129	HAPS_RS09775	DNA repair protein RadA	nucleoside-triphosphatase activity|cellular response to DNA damage stimulus|DNA binding|nucleotide binding|DNA repair|ATP catabolic process|damaged DNA binding|recombinational repair|metal ion binding|ATP binding|DNA-dependent ATPase activity|DNA metabolic	-	-1.198892602	DOWN

			process			
25120019	HAPS_RS11310	hypothetical protein	collagen trimer|extracellular matrix structural constituent	-	4.570704623	UP
7278172	groES	molecular chaperone GroES	cytosol|identical protein binding|protein binding|cytoplasm|ATP binding|cell division|response to heat|protein folding|virion assembly|unfolded protein binding	-	-1.672288981	DOWN
7277921	HAPS_RS07375	glycerol-3-phosphate acyltransferase	lipid metabolic process|phospholipid biosynthetic process|phospholipid metabolic process|transferase activity|integral component of membrane|transferase activity, transferring acyl groups other than amino-acyl groups|glycerol-3-phosphate O-acyltransferase activity|membrane|plasma membrane|acyl-phosphate glycerol-3-phosphate acyltransferase activity	Metabolic pathways|Biosynthesis of secondary metabolites|Glycerophospholipid metabolism|Glycerolipid metabolism	-1.315245742	DOWN
7278087	HAPS_RS06285	phosphate acyltransferase	transferase activity, transferring acyl groups other than amino-acyl groups|transferase activity|cytoplasm|oxidation-reduction process|fatty acid biosynthetic process|phospholipid biosynthetic process|phospholipid metabolic process|lipid metabolic process|oxidoreductase activity, acting on the CH-OH group of donors, NAD or NADP as acceptor	Metabolic pathways|Biosynthesis of secondary metabolites|Glycerophospholipid metabolism|Glycerolipid metabolism	1.763728025	UP
23375310	HAPS_RS01205	bacteriophage protein	biological_process|molecular_function|cellular_component	-	Inf	UP
7278333	HAPS_RS03990	hypothetical protein	-	-	Inf	UP
7278421	HAPS_RS07800	MULTISPECIES: 30S ribosomal protein S19	structural constituent of ribosome|translation	Ribosome	1.776533259	UP
7278896	tuf	elongation factor Tu	response to antibiotic|GTP catabolic process|nucleotide binding|translational elongation|membrane|GTPase activity|plasma membrane|cytoplasm|translation|translation	-	1.881710636	UP

			elongation factor activity|GTP binding|intracellular			
7278814	fusA	elongation factor G	cytoplasm|GTPase activity|GTP binding|translation|translation elongation factor activity|intracellular|translational elongation|GTP catabolic process|nucleotide binding	-	1.778705511	UP
23375418	HAPS_RS06060	hypothetical protein	integral component of membrane|phosphorylation|transport|plasma membrane|sugar:proton symporter activity|membrane|hexose transmembrane transport|glucose transport|protein-N(PI)-phosphohistidine-sugar phosphotransferase activity|phosphoenolpyruvate-dependent sugar phosphotransferase system|transferase activity|carbohydrate transmembrane transport|ion transmembrane transport|carbohydrate transport|glucose transmembrane transporter activity|kinase activity|proton transport	Amino sugar and nucleotide sugar metabolism|Glycolysis / Gluconeogenesis|Phosphotransferase system (PTS)	-1.556622016	DOWN
7278631	HAPS_RS00080	hypothetical protein	-	-	Inf	UP
7276816	HAPS_RS03250	hypothetical protein	tRNA binding|rescue of stalled ribosome|ribosomal large subunit binding|regulation of translation	-	-2.759595383	DOWN
7278941	HAPS_RS05910	N-acetylmannosamine-6-phosphate 2-epimerase	N-acetylneuraminate catabolic process|N-acetylmannosamine metabolic process|metabolic process|N-acetylmannosamine catabolic process|catalytic activity|carbohydrate metabolic process|N-acylglucosamine-6-phosphate 2-epimerase	Amino sugar and nucleotide sugar metabolism	-1.212099579	DOWN

			activity|isomerase activity			
7278345	hslO	molecular chaperone Hsp33	molecular_function|biological_process|cellular_component	-	-1.514463502	DOWN
7277672	HAPS_RS08550	DNA-binding protein	-	-	-1.08712377	DOWN
7277094	rpmE	50S ribosomal protein L31	ribosome|metal ion binding|translation|cytosolic large ribosomal subunit|structural constituent of ribosome|RNA binding|zinc ion binding|rRNA binding|ribonucleoprotein complex	Ribosome	-1.04860361	DOWN
7278417	HAPS_RS07780	50S ribosomal protein L3	structural constituent of ribosome|cytosolic large ribosomal subunit|ribonucleoprotein complex|translation|rRNA binding|RNA binding|ribosome	Ribosome	1.676961795	UP
7277081	HAPS_RS04930	beta-hexosaminidase	hydrolase activity, hydrolyzing O-glycosyl compounds|cytoplasm|metabolic process|hydrolase activity|carbohydrate metabolic process|cell division|hydrolase activity, acting on glycosyl bonds|peptidoglycan biosynthetic process|beta-N-acetylhexosaminidase activity|peptidoglycan turnover|regulation of cell shape	Metabolic pathways|Amino sugar and nucleotide sugar metabolism|beta-Lactam resistance	2.007952895	UP
7277441	HAPS_RS01260	ABC transporter substrate-binding protein	cellular_component|molecular_function|biological_process	-	-1.452132428	DOWN
7276948	HAPS_RS01930	transcriptional regulator	DNA binding|regulation of transcription, DNA-templated|transcription, DNA-templated|sequence-specific DNA binding	-	8.009512211	UP
7277039	HAPS_RS04320	hypothetical protein	-	-	Inf	UP
7278326	uvrC	excinuclease ABC subunit C	excinuclease ABC activity|cellular response to DNA damage stimulus|nuclease activity|excinuclease repair complex|DNA repair|DNA binding|DNA catabolic process, endonucleolytic|cytoplasm|nucleotide-excision	Nucleotide excision repair	1.59023168	UP

			repair|SOS response			
7278325	HAPS_RS03950	protease HtpX	peptidase activity|zinc ion binding|proteolysis|metallopeptidase activity|metalloendopeptidase activity|hydrolase activity|metal ion binding|integral component of membrane|response to temperature stimulus|plasma membrane|membrane	-	-1.286369482	DOWN
23375345	HAPS_RS02560	hypothetical protein	-	-	2.679742762	UP
7278174	HAPS_RS09985	galactose-1-phosphate uridylyltransferase	UTP:galactose-1-phosphate uridylyltransferase activity|galactose metabolic process	Metabolic pathways|Amino sugar and nucleotide sugar metabolism|Galactose metabolism|Prolactin signaling pathway	-1.228100027	DOWN
7277985	HAPS_RS00720	membrane protein	biological_process	-	-1.114777619	DOWN
7278420	HAPS_RS07795	50S ribosomal protein L2	ribosome|transferase activity|translation|intracellular|cytosolic large ribosomal subunit|structural constituent of ribosome|protein binding|RNA binding|zinc ion binding|rRNA binding|ribonucleoprotein complex|large ribosomal subunit	Ribosome	1.726552563	UP
7277083	upp	uracil phosphoribosyltransferase	pyrimidine-containing compound salvage|uracil phosphoribosyltransferase activity	Metabolic pathways|Pyrimidine metabolism	1.692545386	UP
7277216	HAPS_RS07150	DNA mismatch repair protein MutS	mismatch repair|ATP binding	Mismatch repair	1.537570335	UP
7276876	HAPS_RS00355	tol-pal system-associated acyl-CoA thioesterase	membrane|thiolester hydrolase activity|plasma membrane|protein binding|metabolic process|hydrolase activity	-	-1.207215115	DOWN

7278754	HAPS_RS09270	twitching motility protein PilT	migration in host|protein binding	-	6.634897641	UP
7278416	rpsJ	MULTISPECIES: 30S ribosomal protein S10	structural constituent of ribosome|translation	Ribosome	1.579751246	UP
7278425	HAPS_RS07820	MULTISPECIES: 50S ribosomal protein L29	translation|structural constituent of ribosome	Ribosome	1.675799119	UP
7278479	HAPS_RS02340	RNA helicase	cytoplasm|protein binding|RNA helicase activity|ribosomal large subunit assembly|hydrolase activity|ATP-dependent RNA helicase activity|ATP catabolic process|RNA catabolic process|ATP binding|helicase activity|ATP-dependent helicase activity|nucleic acid binding|nucleotide binding|RNA binding|cellular response to cold|hydrolase activity, acting on acid anhydrides|mRNA stabilization	RNA degradation	1.539914284	UP
7276818	HAPS_RS03255	hypothetical protein	plasma membrane|cell wall	-	-1.099796473	DOWN
7277260	HAPS_RS11140	thiol reductase thioredoxin	protein binding	-	-1.521207878	DOWN
7278444	HAPS_RS07920	ribokinase	ribokinase activity|D-ribose metabolic process	Pentose phosphate pathway	-1.004510222	DOWN
23375546	-	-	asexual sporulation|molecular_function	-	-1.492873896	DOWN
7276975	HAPS_RS02060	hypothetical protein	-	-	Inf	UP
25120020	HAPS_RS11315	putative amidophosphoribosyltransferase	-	-	Inf	UP
7278452	HAPS_RS02205	methionine ABC transporter permease	transport|ATPase activity, coupled to transmembrane movement of substances	ABC transporters	-1.105960252	DOWN
7277075	HAPS_RS04490	IS110 family transposase	transposase activity|transposition, DNA-mediated	-	Inf	UP
7278336	HAPS_RS04005	hypothetical protein	-	-	Inf	UP
7276947	HAPS_RS01925	plasmid maintenance protein ParE	cell death|molecular_function	-	Inf	UP
7278511	HAPS_RS02500	IS110 family transposase	transposition, DNA-mediated|transposase activity	-	Inf	UP
7277970	HAPS_RS00645	IS110 family transposase	transposase activity|transposition, DNA-mediated	-	Inf	UP
7277534	HAPS_RS06770	membrane protein	-	-	-1.639390925	DOWN

7277005	metQ	membrane protein	transport|amino acid transport|plasma membrane|membrane	ABC transporters	-1.1174643	DOWN
7278612	HAPS_RS10540	lactoylglutathione lyase	lyase activity|cellular_component|metal ion binding|lactoylglutathione lyase activity|glutathione metabolic process	Pyruvate metabolism	-1.03214369	DOWN
7277831	HAPS_RS04655	PTS glucose transporter subunit IIA	phosphoenolpyruvate-dependent sugar phosphotransferase system|glucose transport	Amino sugar and nucleotide sugar metabolism|Glycolysis / Gluconeogenesis|Phosphotransferase system (PTS)|Starch and sucrose metabolism	-1.358769999	DOWN
23375502	-	-	biological_process|molecular_function	-	-2.107918148	DOWN
7277850	HAPS_RS04750	hypothetical protein	FMN binding|oxidation-reduction process|oxidoreductase activity	-	-1.007827124	DOWN
7276915	HAPS_RS00545	IS110 family transposase	transposition, DNA-mediated|transposase activity	-	7.695204273	UP
25120010	HAPS_RS11265	site-specific DNA-methyltransferase	DNA methylation|site-specific DNA-methyltransferase (adenine-specific) activity|DNA restriction-modification system	-	2.007026782	UP
23375514	-	-	transposase activity|transposition, DNA-mediated	-	Inf	UP
7278198	HAPS_RS10110	peptidyl-prolyl cis-trans isomerase	cytoplasm|membrane|protein folding|peptidyl-prolyl cis-trans isomerase activity|periplasmic space|peptidyl-proline modification|FK506 binding|cytosol|isomerase activity|protein peptidyl-prolyl isomerization	-	-1.058690849	DOWN
7278131	HAPS_RS09785	IS110 family transposase	transposase activity|transposition, DNA-mediated	-	Inf	UP
7278542	HAPS_RS10200	glutamate synthase subunit beta	cellular amino acid biosynthetic process|oxidoreductase activity|oxidation-reduction process|L-glutamate	Metabolic pathways|Biosynthesis of secondary metabolites|Biosynthesis	-1.031793795	DOWN

			biosynthetic process|glutamate synthase (NADPH) activity|nitrogen compound metabolic process|metal ion binding|glutamate biosynthetic process|4 iron, 4 sulfur cluster binding|flavin adenine dinucleotide binding|iron-sulfur cluster binding|protein binding|oxidoreductase activity, acting on the CH-NH2 group of donors, NAD or NADP as acceptor	of antibiotics|Microbial metabolism in diverse environments|Biosynthesis of amino acids|Alanine, aspartate and glutamate metabolism|Nitrogen metabolism		
7278735	dnaK	molecular chaperone DnaK	protein domain specific binding|chaperone binding	RNA degradation|Longevity regulating pathway - worm	-1.220601416	DOWN
7278870	-	-	-	-	-2.738156474	DOWN
7276961	HAPS_RS01990	antitoxin	-	-	-1.44482212	DOWN
7277754	HAPS_RS10745	cytochrome c-type biogenesis protein CcmE	catalytic activity|cytochrome complex assembly	-	-1.07284192	DOWN
23375476	HAPS_RS08310	hypothetical protein	polyamine-transporting ATPase activity|putrescine transport|spermidine transport	ABC transporters	-1.688911224	DOWN
7278769	HAPS_RS09340	hypothetical protein	-	-	Inf	UP
7277356	HAPS_RS09615	phosphoribosylformylglycinamidine synthase	purine ribonucleotide biosynthetic process|phosphoribosylformylglycinamidine synthase activity	Metabolic pathways|Biosynthesis of secondary metabolites|Biosynthesis of antibiotics|Purine metabolism	1.511020219	UP
7277411	HAPS_RS01125	cysteine/glutathione ABC transporter ATP-binding protein/permease CydC	ATP binding|transmembrane transport|integral component of membrane|glutathione transmembrane transport|transport|ATPase activity, coupled to transmembrane movement of substances|plasma membrane|metabolic process|membrane|ATPase activity|cysteine export|nucleoside-triphosphatase activity|regulation of heme biosynthetic process|ATP catabolic process|nucleotide binding	ABC transporters	1.526579839	UP

25120006	HAPS_RS11245	hypothetical protein	-	-	Inf	UP
7276957	HAPS_RS01970	hypothetical protein	biological_process|molecular_function|cellular_component	-	-2.507194183	DOWN
7278893	HAPS_RS05685	anthranilate synthase component II	anthranilate synthase activity|tryptophan biosynthetic process	Folate biosynthesis	1.457116197	UP
7277508	fis	Fis family transcriptional regulator	regulation of transcription, DNA-templated	-	-1.304025647	DOWN
7278176	HAPS_RS09995	integrase	transposition, DNA-mediated|transposase activity	-	1.531919528	UP
7278677	HAPS_RS08915	cation transporter	transport|response to metal ion|transporter activity	-	Inf	UP
7277751	HAPS_RS10730	glycosyl transferase	polysaccharide biosynthetic process|transferase activity, transferring glycosyl groups	-	1.420384752	UP
7277503	HAPS_RS06615	TonB system transport protein ExbD	transport|transporter activity	-	2.0932457	UP
7277379	HAPS_RS09715	23S rRNA pseudouridylate synthase B	intramolecular transferase activity|pseudouridine synthesis|pseudouridine synthase activity|rRNA processing|RNA binding|enzyme-directed rRNA pseudouridine synthesis|isomerase activity	-	1.416302737	UP
25120024	-	-	-	-	-2.307782679	DOWN
7278686	HAPS_RS08955	hydroxymethylpyrimidine/phosphomethylpyrimidine kinase	phosphomethylpyrimidine kinase activity|thiamine biosynthetic process	Metabolic pathways|Thiamine metabolism	Inf	UP
23375506	-	-	glutathione biosynthetic process|transferase activity, transferring acyl groups|glutathione metabolic process|glutathione hydrolase activity|hydrolase activity|transferase activity|periplasmic space|proteolysis|gamma-glutamyltransferase activity|peptidase activity	Metabolic pathways|Glutathione metabolism|Taurine and hypotaurine metabolism	1.584578524	UP
7277875	HAPS_RS04875	hypothetical protein	molecular_function|biological_process|cellular_component	-	-1.007468514	DOWN
7277752	HAPS_RS10735	amylovoran biosynthesis protein AmsE	polysaccharide biosynthetic process|transferase activity, transferring glycosyl groups	-	1.428396394	UP

7278578	HAPS_RS10375	oxidoreductase	biological_process|molecular_function|cellular_component	Metabolic pathways|Arginine and proline metabolism	1.590917489	UP
7278685	HAPS_RS08950	thiamine phosphate synthase	thiamine-phosphate diphosphorylase activity|thiamine biosynthetic process	Metabolic pathways|Thiamine metabolism	Inf	UP
7278166	rpmG	MULTISPECIES: 50S ribosomal protein L33	translation|structural constituent of ribosome	Ribosome	-1.129883585	DOWN
7277114	HAPS_RS05080	PLP-dependent threonine dehydratase	threonine metabolic process	Metabolic pathways|Biosynthesis of secondary metabolites|Biosynthesis of antibiotics|Biosynthesis of amino acids|Carbon metabolism|Glycine, serine and threonine metabolism|Valine, leucine and isoleucine biosynthesis	1.357426547	UP
7278352	HAPS_RS04085	6-phosphogluconolactonase	pentose-phosphate shunt|6-phosphogluconolactonase activity	Metabolic pathways|Biosynthesis of secondary metabolites|Biosynthesis of antibiotics|Microbial metabolism in diverse environments|Carbon metabolism|Pentose phosphate pathway	1.343231896	UP
7278426	HAPS_RS07825	30S ribosomal protein S17	cytosolic small ribosomal subunit|structural constituent of ribosome|translation|SSU rRNA binding|ribosome|response to antibiotic|rRNA binding|ribonucleoprotein complex|RNA binding	Ribosome	1.400550991	UP
7277330	HAPS_RS01725	type I restriction endonuclease HindVIIP subunit M	Type I site-specific deoxyribonuclease complex|DNA-methyltransferase activity|DNA modification	-	1.302905186	UP
7277113	glmM	phosphoglucosamine mutase	peptidoglycan biosynthetic process|phosphoglucosamine	Metabolic pathways|Biosynthesis of	1.379694839	UP

			mutase activity|lipopolysaccharide biosynthetic process	antibiotics|Amino sugar and nucleotide sugar metabolism		
23375398	-	-	-	-	-2.854685455	DOWN
7277431	HAPS_RS01215	hypothetical protein	-	-	Inf	UP
7278751	HAPS_RS09255	phosphoglycolate phosphatase	-	-	Inf	UP
7278835	HAPS_RS05395	molecular chaperone HtpG	protein folding|response to heat|ATP binding|unfolded protein binding|protein binding|plasma membrane|cytoplasm|membrane|cytosol|ATPase activity, coupled|cellular response to DNA damage stimulus|ATP catabolic process|nucleotide binding	-	-1.014805653	DOWN
7277801	-	-	-	-	-2.220454468	DOWN
7277279	guaA	GMP synthetase	GMP synthase (glutamine-hydrolyzing) activity|purine ribonucleotide biosynthetic process	Metabolic pathways	1.291412301	UP
7278043	nusA	transcription termination protein NusA	regulation of DNA-templated transcription, termination|nucleotide binding|DNA binding|transcription antitermination|DNA repair|sequence-specific DNA binding transcription factor activity|RNA binding|cytosol|transcription, DNA-templated|protein binding|regulation of transcription, DNA-templated|cytoplasm|DNA-templated transcription, termination	-	1.304137217	UP
7278478	HAPS_RS02335	tRNA-specific adenosine deaminase	zinc ion binding|metal ion binding|tRNA wobble adenosine to inosine editing|catalytic activity|tRNA-specific adenosine deaminase activity|hydrolase activity|tRNA processing	-	1.895945922	UP
7278973	HAPS_RS06065	MULTISPECIES: HslU--HslV peptidase proteolytic	hydrolase activity|metal ion binding|response to	-	-1.424634974	DOWN

		subunit	heat|catalytic activity|protein catabolic process|protein binding|identical protein binding|cytoplasm|HslUV protease complex|peptidase activity|threonine-type endopeptidase activity|cytosol|proteasome core complex|metabolic process|proteolysis involved in cellular protein catabolic process|proteolysis			
23375550	-	-	-	-	8.391384501	UP
7277471	-	-	-	-	-3.711941922	DOWN
7278380	HAPS_RS07620	iron-regulated lipoprotein	molecular_function|biological_process	-	Inf	UP
7278127	HAPS_RS09765	DNA methyltransferase	nucleic acid phosphodiester bond hydrolysis|mismatch repair|endonuclease activity	-	-1.553556325	DOWN
7278670	HAPS_RS08880	DsbA family protein	cellular_component|molecular_function|biological_process	-	Inf	UP
7278845	HAPS_RS05455	DNA-binding protein	identical protein binding	-	-1.086418628	DOWN
7278190	panF	sodium/panthothenate symporter	membrane|plasma membrane|transport|sodium ion binding|pantothenate transmembrane transport|integral component of membrane|ion transport|transporter activity|transmembrane transport|pantothenate transmembrane transporter activity|sodium ion transmembrane transporter activity|sodium ion transport|sodium ion export|symporter activity	-	1.295215737	UP
7278872	HAPS_RS05580	antirepressor	-	-	1.293662491	UP
23375441	HAPS_RS07235	hypothetical protein	-	-	3.369074664	UP
7277780	HAPS_RS10870	DNA topoisomerase IV subunit B	ATP catabolic process|chromosome|isomerase activity|magnesium ion binding|chromosome organization|sister chromatid cohesion|metal ion binding|DNA topological change|nucleotide binding|DNA	-	1.371212155	UP

			binding|response to antibiotic|plasmid partitioning|DNA topoisomerase activity|nucleoid|chromosome segregation|DNA topoisomerase type II (ATP-hydrolyzing) activity|ATP binding|DNA-dependent DNA replication			
7278874	HAPS_RS05590	hypothetical protein	-	-	1.256242643	UP
7278369	fabG	beta-ketoacyl-ACP reductase	3-oxoacyl-[acyl-carrier-protein] reductase (NADPH) activity|fatty acid elongation|NADP binding	Metabolic pathways|Fatty acid metabolism|Fatty acid biosynthesis|Biotin metabolism|Biosynthesis of unsaturated fatty acids	1.175131282	UP
7277214	HAPS_RS07140	diaminopimelate decarboxylase	lysine biosynthetic process via diaminopimelate|diaminopimelate decarboxylase activity	Metabolic pathways|Biosynthesis of secondary metabolites|Biosynthesis of antibiotics|Microbial metabolism in diverse environments|Biosynthesis of amino acids|Lysine biosynthesis	1.329350388	UP
7277022	HAPS_RS04235	hypothetical protein	-	-	Inf	UP
7276725	HAPS_RS02835	hypothetical protein	-	-	2.074252495	UP
7278839	HAPS_RS05425	ABC transporter ATP-binding protein	oligopeptide transport|ATP binding|oligopeptide-transporting ATPase activity	Quorum sensing	1.307330261	UP
7276863	HAPS_RS00285	IS110 family transposase	transposase activity|transposition, DNA-mediated	-	5.345539731	UP
23375551	HAPS_RS10235	transposase	-	-	4.716500124	UP
7278887	HAPS_RS05655	ribonucleoside-triphosphate reductase	-	-	-1.035149294	DOWN
7278768	HAPS_RS09335	hypothetical protein	-	-	Inf	UP
7276856	HAPS_RS00250	tyrosine phosphatase	phosphoprotein phosphatase activity|peptidyl-tyrosine	-	1.223137329	UP

			dephosphorylation|polysaccharide biosynthetic process|colanic acid biosynthetic process|hydrolase activity|protein tyrosine phosphatase activity			
7277432	HAPS_RS01220	hypothetical protein	-	-	Inf	UP
7277085	HAPS_RS04950	lipooligosaccharide D-glycero-D-manno-heptosyltransferase	transferase activity|lipopolysaccharide biosynthetic process|transferase activity, transferring glycosyl groups|lipopolysaccharide core region biosynthetic process|membrane|metabolic process|lipopolysaccharide N-acetylglucosaminyltransferase activity	Metabolic pathways|Lipopolysaccharide biosynthesis	1.319723841	UP
7278938	HAPS_RS05895	GTPase HflX	rRNA binding|ribosome binding|nucleotide binding|GTP catabolic process|viral latency|GTP binding|cytoplasm|GTPase activity|ATP binding|response to heat	-	1.243443398	UP
23375516	HAPS_RS09370	hypothetical protein	-	-	1.71049479	UP
23375467	-	-	transposase activity|transposition, DNA-mediated	-	6.511675154	UP
7276730	HAPS_RS02860	hypothetical protein	-	-	-1.708255807	DOWN
7277291	HAPS_RS01545	transcriptional regulator	biological_process	-	-1.167712614	DOWN
7277304	HAPS_RS01600	tRNA (adenosine(37)-N6)-threonylcarbamoyltransferase complex dimerization subunit type 1 TsaB	tRNA processing|cytoplasm|identical protein binding|protein binding|metallopeptidase activity|threonylcarbamoyladenosine biosynthetic process	-	1.439925028	UP
7278863	HAPS_RS05545	ABC transporter permease	transport|ATPase activity, coupled to transmembrane movement of substances	-	2.067228954	UP
7277038	HAPS_RS04315	hypothetical protein	molecular_function|biological_process|cellular_component	-	-1.152610189	DOWN
7278873	HAPS_RS05585	transposase	transposition, DNA-mediated|transposase activity	-	1.66889389	UP
7277511	ubiB	ubiquinone biosynthesis protein UbiB	protein kinase activity|nucleotide binding|transferase activity, transferring phosphorus-containing	-	1.169910598	UP

			groups|ubiquinone biosynthetic process|regulation of ubiquinone biosynthetic process|phosphorylation|transferase activity|integral component of membrane|ATP binding|protein phosphorylation|membrane|kinase activity|plasma membrane			
7277600	HAPS_RS08195	acyl carrier protein	fatty acid biosynthetic process|ACP phosphopantetheine attachment site binding involved in fatty acid biosynthetic process	-	-1.735364128	DOWN
7277246	HAPS_RS11070	sodium-dependent transporter	solute:sodium symporter activity|membrane|transport	-	1.883284225	UP
7276769	HAPS_RS03035	hypothetical protein	-	-	-1.772272167	DOWN
7278057	HAPS_RS06145	phosphatidate cytidylyltransferase	lipid metabolic process|phospholipid biosynthetic process|transferase activity, transferring phosphorus-containing groups|nucleotidyltransferase activity|phosphatidate cytidylyltransferase activity|plasma membrane|membrane|CDP-diacylglycerol biosynthetic process|transferase activity|integral component of membrane	Metabolic pathways|Biosynthesis of secondary metabolites|Glycerophospholipid metabolism	1.162025812	UP
7277873	HAPS_RS04865	ABC transporter ATPase	metabolic process|cytoplasm|ATPase activity|regulation of transposon integration|ATP binding|transport|ATP catabolic process|DNA binding|nucleotide binding|nucleoside-triphosphatase activity	-	1.421396648	UP
7277658	-	-	-	-	-2.523280226	DOWN
7278319	HAPS_RS03920	3-phenylpropionic acid transporter	symporter activity|membrane|plasma membrane|transport|integral component of membrane|transmembrane transport	-	1.165228183	UP

7276896	cysS	cysteine--tRNA ligase	cysteine-tRNA ligase activity|cysteinyl-tRNA aminoacylation	Aminoacyl-tRNA biosynthesis	1.267869049	UP
7278191	HAPS_RS10075	membrane protein	plasma membrane|membrane|integral component of membrane	-	1.665177326	UP
7277080	rumB	23S rRNA (uracil(747)-C(5))-methyltransferase RlmC	rRNA base methylation|metal ion binding|4 iron, 4 sulfur cluster binding|RNA methyltransferase activity|transferase activity|RNA processing|methyltransferase activity|iron-sulfur cluster binding|RNA metabolic process|methylation|rRNA (uridine-C5-)-methyltransferase activity|rRNA methylation|iron ion binding|rRNA (uridine) methyltransferase activity|rRNA processing	-	1.217590145	UP
7277259	HAPS_RS11135	membrane protein	transport|integral component of membrane|plasma membrane|amino acid transport|membrane	-	1.731031417	UP
7277938	HAPS_RS07455	inositol 2-dehydrogenase	metabolic process|oxidoreductase activity	Metabolic pathways|Biosynthesis of antibiotics|Microbial metabolism in diverse environments	1.24949581	UP
23375296	-	-	molecular_function|biological_process	-	1.147948329	UP
7277910	HAPS_RS07320	membrane protein	cellular_component|molecular_function|biological_process	-	1.26116693	UP
23375510	-	-	-	-	5.816937878	UP
7277235	HAPS_RS11020	hypothetical protein	hydrolase activity|metal ion binding|metabolic process	-	1.320854606	UP
7278894	HAPS_RS05690	DNA recombination protein RmuC	molecular_function|biological_process|cellular_component	-	1.224214483	UP
7278907	HAPS_RS05750	tRNA(ANN) t(6)A37 threonylcarbamoyladenosine modification protein	cytoplasm|transferase activity|ATP binding|tRNA processing|tRNA binding|double-stranded RNA binding|rRNA processing|threonylcarbamoyladenosine biosynthetic process|ribosome biogenesis|nucleotide binding|nucleotidyltransferase activity|RNA binding	-	1.124292548	UP

7277702	HAPS_RS08720	transcriptional regulator	regulation of transcription, DNA-templated|negative regulation of transcription, DNA-templated|sequence-specific DNA binding transcription factor activity|D-gluconate catabolic process|transcription, DNA-templated|positive regulation of transcription, DNA-templated|cellular response to DNA damage stimulus|DNA binding	-	1.209402561	UP
7278061	lpxD	UDP-3-O-(3-hydroxymyristoyl)glucosamine N-acyltransferase	lipid metabolic process|lipid A biosynthetic process|response to antibiotic|N-acyltransferase activity|identical protein binding|cytoplasm|transferase activity, transferring acyl groups|transferase activity, transferring acyl groups other than amino-acyl groups|transferase activity	Metabolic pathways|Lipopolysaccharide biosynthesis	1.219070576	UP
7278351	HAPS_RS04080	hypothetical protein	molecular_function|biological_process|cellular_component	-	1.130996622	UP
7278042	HAPS_RS01005	ribosome maturation factor	ribosomal small subunit biogenesis|cytoplasm|ribosome biogenesis|translation|ribosomal small subunit assembly	-	1.826979944	UP
7276793	-	-	-	-	-2.087064171	DOWN
7277276	HAPS_RS01460	IMP dehydrogenase	purine ribonucleotide biosynthetic process|IMP dehydrogenase activity	Metabolic pathways|Biosynthesis of secondary metabolites|Purine metabolism|Drug metabolism - other enzymes	1.125655003	UP
23375311	HAPS_RS01210	Mu protein C/ Mor gp17 transcription regulator	-	-	Inf	UP
7278891	HAPS_RS05675	anaerobic ribonucleoside-triphosphate reductase	molecular_function|biological_process|cellular_component	Metabolic pathways|Purine metabolism|Pyrimidine metabolism	1.13314156	UP
7278014	HAPS_RS00865	NrdR family transcriptional regulator	DNA binding|nucleotide binding|zinc ion binding|transcription, DNA-templated|double-stranded	-	1.176494122	UP

			DNA binding|negative regulation of transcription, DNA-templated|regulation of transcription, DNA-templated|ATP binding|metal ion binding			
7277211	HAPS_RS07125	xanthine phosphoribosyltransferase	membrane|plasma membrane|purine ribonucleoside salvage|transferase activity|GMP salvage|metal ion binding|transferase activity, transferring glycosyl groups|XMP salvage|hypoxanthine phosphoribosyltransferase activity|IMP salvage|xanthine phosphoribosyltransferase activity|cytosol|magnesium ion binding|nucleoside metabolic process	Metabolic pathways|Biosynthesis of secondary metabolites|Purine metabolism	2.666628627	UP
23375561	HAPS_RS10920	beta-D-galactosidase	hydrolase activity, hydrolyzing O-glycosyl compounds|metabolic process|hydrolase activity|beta-galactosidase activity|carbohydrate metabolic process|catalytic activity|hydrolase activity, acting on glycosyl bonds|beta-galactosidase complex|carbohydrate binding	Metabolic pathways|Galactose metabolism	1.205718699	UP
7277869	HAPS_RS04845	ABC transporter substrate-binding protein	periplasmic space|oligopeptide transport|protein transport|transport|transporter activity|peptide transport	ABC transporters|Quorum sensing|beta-Lactam resistance	1.091938882	UP
7278637	HAPS_RS00110	ABC transporter permease	transport	-	1.079378743	UP
7276728	HAPS_RS02850	translocation protein TolB precursor	-	-	1.838755868	UP
7278741	rpmH	MULTISPECIES: 50S ribosomal protein L34	structural constituent of ribosome	Ribosome	-1.078295064	DOWN
7278023	HAPS_RS00910	BAX inhibitor protein	membrane|plasma membrane|integral component of membrane|regulation of proteolysis|negative regulation of apoptotic process	-	1.393648037	UP
7278121	-	-	-	-	1.52645721	UP
7276720	HAPS_RS02820	hypothetical protein	-	-	2.182917168	UP

7277800	rluD	23S rRNA pseudouridine(1911/1915/1917) synthase	protein binding|enzyme-directed rRNA pseudouridine synthesis|ribosomal large subunit assembly|pseudouridine synthesis|rRNA processing|pseudouridine synthase activity|isomerase activity|RNA binding	-	1.118745367	UP
7277212	HAPS_RS07130	IS110 family transposase	transposition, DNA-mediated|transposase activity	-	3.73510795	UP
7277551	HAPS_RS06855	3-deoxy-D-manno-octulosonic acid transferase	lipopolysaccharide biosynthetic process|Kdo2-lipid A biosynthetic process|transferase activity|integral component of membrane|biosynthetic process|plasma membrane|membrane|lipopolysaccharide core region biosynthetic process|lipid A biosynthetic process	Metabolic pathways|Lipopolysaccharide biosynthesis	1.061251634	UP
7278507	HAPS_RS02480	Nif3-like dinuclear metal center hexameric protein	cellular_component|molecular_function|biological_process	-	1.082041732	UP
7278846	HAPS_RS05460	Na+/H+ antiporter	biological_process|molecular_function	-	1.71176435	UP
23375497	-	-	pyridoxal phosphate binding|transferase activity|catalytic activity|biosynthetic process|regulation of transcription, DNA-templated|sequence-specific DNA binding transcription factor activity|transaminase activity|transcription, DNA-templated|DNA binding	-	Inf	UP
7278063	HAPS_RS06175	acyl	acyl-[acyl-carrier-protein]-UDP-N-acetylglucosamine O-acyltransferase activity|lipid A biosynthetic process|lipid metabolic process|transferase activity|lipid biosynthetic process|transferase activity, transferring acyl groups|identical protein binding|cytoplasm	Metabolic pathways|Lipopolysaccharide biosynthesis|Cationic antimicrobial peptide (CAMP) resistance	1.050861878	UP
7276857	HAPS_RS00255	tyrosine protein kinase	nucleotide binding|protein tyrosine kinase activity|regulation of catalytic activity|peptidyl-tyrosine autophosphorylation|lipopolysaccharide biosynthetic process|capsule polysaccharide biosynthetic	-	1.166272544	UP

			process|enzyme regulator activity|phosphorylation|transferase activity|integral component of membrane|ATP binding|kinase activity|membrane|identical protein binding|plasma membrane			
7277504	HAPS_RS06620	TonB-system energizer ExbB	transport|transporter activity	-	2.177624027	UP
23375475	HAPS_RS08305	hypothetical protein	-	-	1.894948688	UP
7277018	HAPS_RS04220	integrase	molecular_function|biological_process|cellular_component	-	-1.289124756	DOWN
7277434	HAPS_RS01230	hypothetical protein	-	-	Inf	UP
7278025	HAPS_RS00920	protein disulfide-isomerase	protein folding|cell redox homeostasis|metabolic process|outer membrane-bounded periplasmic space|protein disulfide isomerase activity|oxidation-reduction process|protein disulfide oxidoreductase activity|periplasmic space	-	1.16740708	UP
7277652	HAPS_RS08445	DNA repair protein HhH-GPD	-	-	1.57109199	UP
7278921	HAPS_RS05815	MULTISPECIES: 30S ribosomal protein S21	translation|ribonucleoprotein complex|structural constituent of ribosome|cytosolic small ribosomal subunit|ribosome	Ribosome	-1.563858769	DOWN
7278155	HAPS_RS09895	dihydroorotate dehydrogenase 2	pyrimidine ribonucleotide biosynthetic process|dihydroorotate oxidase activity	Metabolic pathways|Pyrimidine metabolism	1.338332528	UP
7277213	HAPS_RS07135	transposase	-	-	1.131545332	UP
7277553	dnaE	DNA polymerase III subunit alpha	DNA replication|DNA-directed DNA polymerase activity|DNA polymerase III complex	Metabolic pathways|Purine metabolism|Pyrimidine metabolism|Homologous recombination|Mismatch repair|DNA replication	1.143680298	UP

23375499	-	-	DNA binding|transposition, DNA-mediated|transposase activity	-	Inf	UP
7278687	HAPS_RS08960	hydroxyethylthiazole kinase	phosphomethylpyrimidine kinase activity|thiamine biosynthetic process	Metabolic pathways	Inf	UP
7277298	HAPS_RS01575	bifunctional glutamine synthetase adenylyltransferase/deadenyltransferase	metabolic process|[glutamate-ammonia-ligase] adenylyltransferase activity|nucleotide binding|ATP binding|nucleotidyltransferase activity|transferase activity	-	1.288212171	UP
7277087	HAPS_RS04955	ADP-heptose--LPS heptosyltransferase	-	-	1.217343796	UP
7276727	HAPS_RS02845	recombinase	protein binding|DNA metabolic process|protein-DNA complex|DNA synthesis involved in double-strand break repair via homologous recombination|DNA recombination|DNA binding|DNA duplex unwinding|single-stranded DNA binding	-	1.822591535	UP
7277126	-	-	transport|carbohydrate transmembrane transport|integral component of membrane|transporter activity|cellular response to mercury ion|transmembrane transport|metal ion binding|glycerol transport|membrane|plasma membrane|glycerol transmembrane transporter activity|glycerol channel activity	-	2.528404649	UP
7278857	HAPS_RS05515	peptidylprolyl isomerase	protein folding|peptidyl-prolyl cis-trans isomerase activity|integral component of membrane|plasma membrane|identical protein binding|membrane|isomerase activity|protein peptidyl-prolyl isomerization	-	1.121745418	UP
7276724	HAPS_RS02830	hypothetical protein	-	-	2.457901143	UP
23375493	HAPS_RS08690	hypothetical protein	-	-	-1.287420445	DOWN
7278348	HAPS_RS04065	phosphogluconate dehydrogenase	pentose-phosphate shunt|phosphogluconate dehydrogenase	Metabolic pathways|Biosynthesis of	1.020261881	UP

		(NADP(+)-dependent, decarboxylating)	(decarboxylating) activity	secondary metabolites|Biosynthesis of antibiotics|Microbial metabolism in diverse environments|Carbon metabolism|Pentose phosphate pathway|Glutathione metabolism		
7278342	rho	transcription termination factor Rho	hydrolase activity|helicase activity|DNA-templated transcription, termination|RNA-dependent ATPase activity|ATP binding|membrane|regulation of transcription, DNA-templated|protein binding|nucleoside-triphosphatase activity|RNA binding|transcription, DNA-templated|nucleotide binding|nucleic acid binding|ATP catabolic process	RNA degradation	1.06071099	UP
7278106	-	-	-	-	-2.665273333	DOWN
7277723	HAPS_RS10590	hypothetical protein	biological_process|molecular_function	-	1.821726212	UP
7276676	HAPS_RS01015	translation initiation factor IF-2	GTP catabolic process|nucleotide binding|translation initiation factor activity|membrane|GTPase activity|cytoplasm|protein binding|translational initiation|intracellular|GTP binding|translation|ribosomal small subunit binding|guanosine tetraphosphate binding	-	1.012254957	UP
23375463	HAPS_RS08180	hypothetical protein	proteolysis	-	1.09531219	UP
7277000	HAPS_RS02175	tRNA cytosine(34) acetyltransferase TmcA	transferase activity|tRNA wobble cytosine modification|ATP binding|tRNA processing|tRNA binding|tRNA acetylation|transferase activity, transferring acyl groups|cytoplasm|tRNA N-acetyltransferase activity|N-acetyltransferase activity|RNA binding|nucleotide binding	-	1.046484697	UP

7278639	HAPS_RS00120	RelE toxin	biological_process|translation termination factor activity	-	1.359925558	UP
7276685	HAPS_RS02655	phage capsid scaffolding protein	-	-	3.148295814	UP
7276987	HAPS_RS02115	DNA topoisomerase III	DNA topoisomerase type I activity|DNA recombination|metal ion binding|ATP binding|chromosome separation|DNA topoisomerase activity|magnesium ion binding|isomerase activity|DNA topological change|nucleotide binding|DNA binding	-	1.001653974	UP
7278254	HAPS_RS03600	UDP-N-acetylglucosamine 1-carboxyvinyltransferase	UDP-N-acetylglucosamine 1-carboxyvinyltransferase activity	Metabolic pathways|Amino sugar and nucleotide sugar metabolism	1.063975553	UP
7278300	ubiA	4-hydroxybenzoate octaprenyltransferase	4-hydroxybenzoate octaprenyltransferase activity|ubiquinone biosynthetic process|transferase activity, transferring alkyl or aryl (other than methyl) groups|transferase activity|integral component of membrane|prenyltransferase activity|plasma membrane|membrane	Metabolic pathways|Biosynthesis of secondary metabolites|Ubiquinone and other terpenoid-quinone biosynthesis	1.010266215	UP
7278869	-	-	-	-	-3.195571512	DOWN
23375424	-	-	biological_process|molecular_function	-	1.011636729	UP
7277653	HAPS_RS08450	ATP-dependent protease	biological_process	-	1.58419914	UP
7278259	HAPS_RS03625	ABC transporter permease	biological_process|molecular_function|cellular_component	ABC transporters	1.231430885	UP
7277425	HAPS_RS01195	YggW family oxidoreductase	4 iron, 4 sulfur cluster binding|cytoplasm|porphyrin-containing compound biosynthetic process	Metabolic pathways|Biosynthesis of secondary metabolites|Porphyrin and chlorophyll metabolism	1.022953413	UP
23375417	-	-	-	-	2.731983021	UP
7277020	HAPS_RS04230	IclR family transcriptional regulator	-	-	3.511161788	UP
23375422	-	-	-	-	-2.76764252	DOWN
7278614	HAPS_RS10550	hypothetical protein	-	-	1.039555561	UP

7276731	HAPS_RS02865	hypothetical protein	hyphal cell wall|fungal-type cell wall|fungal-type cell wall organization|molecular_function	-	5.45597025	UP
7278632	HAPS_RS00085	hypothetical protein	molecular_function|biological_process	-	2.724075192	UP
7276855	HAPS_RS00245	sugar transporter	polysaccharide transport|polysaccharide transmembrane transporter activity|polysaccharide biosynthetic process|porin activity|pore complex|membrane|carbohydrate transport|cell outer membrane|transport|carbohydrate transmembrane transport|integral component of membrane|ion transport|transmembrane transport	-	1.043807665	UP
7277278	-	-	-	-	1.115572067	UP
7277871	HAPS_RS04855	peptide ABC transporter permease	protein transport|transport|peptide transport|transporter activity|integral component of membrane|plasma membrane|membrane	ABC transporters|Quorum sensing|beta-Lactam resistance	1.01657303	UP
7277427	HAPS_RS01200	hypothetical protein	-	-	2.144323913	UP
7278019	HAPS_RS00890	cell division protein ZapB	cellular_component|biological_process|molecular_function	-	-1.37228185	DOWN
7278049	HAPS_RS06105	permease	molecular_function|biological_process	-	1.379247589	UP
7278001	HAPS_RS00800	sodium/glutamate symporter	symporter activity|sodium ion transport|glutamate:sodium symporter activity|amino acid transmembrane transport|membrane|plasma membrane|sodium ion transmembrane transport|ion transport|integral component of membrane|L-alpha-amino acid transmembrane transport|transmembrane transport|L-glutamate transport|ion transmembrane transport|transport	-	3.373882219	UP
7278305	HAPS_RS03850	hypothetical protein	-	-	1.139330327	UP
7278055	HAPS_RS06135	hydroxyacylglutathione hydrolase	hydroxyacylglutathione hydrolase activity|glutathione	Pyruvate metabolism	1.050991993	UP

			metabolic process			
7278580	-	-	-	-	-1.703962017	DOWN
23375542	HAPS_RS10015	hypothetical protein	-	-	-1.987869589	DOWN
23375472	HAPS_RS08265	hypothetical protein	-	-	2.362678189	UP
23375393	HAPS_RS04640	hypothetical protein	-	-	3.999989343	UP
7278260	HAPS_RS03630	ABC transporter ATP-binding protein	ATPase activity, coupled to transmembrane movement of substances|transport	ABC transporters	1.196917148	UP
7278623	HAPS_RS00040	fumarate reductase	fermentation|membrane|plasma membrane|anaerobic respiration|electron carrier activity|integral component of membrane|fumarate metabolic process	Metabolic pathways|Biosynthesis of secondary metabolites|Biosynthesis of antibiotics|Microbial metabolism in diverse environments|Carbon metabolism|Pyruvate metabolism|Two-component system|Carbon fixation pathways in prokaryotes|Citrate cycle (TCA cycle)|Oxidative phosphorylation|Butanoate metabolism	1.500358976	UP
23375316	HAPS_RS01465	exopolysaccharide biosynthesis protein	-	-	1.065819094	UP
7278053	HAPS_RS06125	phosphatidylglycerophosphatase	glycerophospholipid biosynthetic process|cell outer membrane|dephosphorylation|hydrolase activity|diacylglycerol diphosphate phosphatase activity|catalytic activity|phosphatidylglycerophosphatase activity|peptidoglycan biosynthetic process|phosphatidylglycerol biosynthetic process|lipid metabolic process|lipid catabolic	Metabolic pathways|Glycerophospholipid metabolism	1.029222961	UP

			process|undecaprenyl-diphosphatase activity|membrane|plasma membrane|integral component of plasma membrane|integral component of membrane|phospholipid catabolic process|phosphatidate phosphatase activity			
7278917	HAPS_RS05795	tRNA dihydrouridine(20/20a) synthase DusA	oxidoreductase activity|tRNA dihydrouridine synthase activity|oxidation-reduction process|flavin adenine dinucleotide binding|catalytic activity|tRNA processing|tRNA dihydrouridine synthesis	-	1.136895347	UP
7278675	HAPS_RS08905	hypothetical protein	-	-	Inf	UP
7276847	HAPS_RS00205	Lsg locus protein 1	molecular_function|polysaccharide biosynthetic process	-	1.045683871	UP
7276924	HAPS_RS00590	cell division protein FtsW	peptidoglycan transport|plasma membrane|protein binding|cell division site|membrane|lipid-linked peptidoglycan transport|integral component of plasma membrane|lipid-linked peptidoglycan transporter activity|transport|cell division|carbohydrate derivative transport|integral component of membrane|peptidoglycan biosynthetic process|regulation of cell shape	Cell cycle - Caulobacter	1.024133388	UP
7277047	HAPS_RS04360	hypothetical protein	molecular_function|biological_process|cellular_component	-	-1.358163484	DOWN
7277440	HAPS_RS01255	ABC transporter permease	anion transport|anion transmembrane transporter activity	-	-1.074995617	DOWN
7278365	HAPS_RS04150	rRNA methyltransferase	transferase activity|RNA methylation|RNA methyltransferase activity|RNA binding|RNA processing|methyltransferase activity|methylation	-	1.060122077	UP
7277860	-	-	cellular_component|biological_process|molecular_function	-	Inf	UP
7276809	HAPS_RS03215	potassium transporter	potassium ion transport|potassium ion transmembrane transport|glutathione-regulated potassium exporter activity	-	1.09092493	UP

7278292	HAPS_RS03780	terminase	-	-	2.361942275	UP
7278689	HAPS_RS08970	protease TldD	proteolysis	-	Inf	UP
23375307	HAPS_RS01030	hypothetical protein	transposase activity|transposition, DNA-mediated	-	-1.394767921	DOWN
23375426	-	-	-	-	Inf	UP
23375375	HAPS_RS03755	predicted ATPase involved in chromosome partitioning	ATPase activity|cytokinesis	-	1.399279576	UP
7278467	HAPS_RS02280	outer membrane-stress sensor serine endopeptidase DegS	cellular response to misfolded protein|intrinsic component of plasma membrane|serine-type endopeptidase activity	-	1.155699213	UP
7278591	-	-	-	-	-2.52616732	DOWN
7278296	HAPS_RS03805	transcriptional regulator	-	-	2.061810299	UP
7276794	apaH	bis(5\-nucleosyl)-tetraphosphatase (symmetrical)	nucleobase-containing compound metabolic process|bis(5'-nucleosyl)-tetraphosphatase (symmetrical) activity	Purine metabolism	1.009395891	UP
23375555	HAPS_RS10515	hypothetical protein	-	-	1.569903236	UP
7277023	HAPS_RS04240	hypothetical protein	cellular_component|molecular_function|biological_process	-	-1.771236342	DOWN
7278844	HAPS_RS05450	formyltetrahydrofolate deformylase	purine ribonucleotide biosynthetic process|hydroxymethyl-, formyl- and related transferase activity|formyltetrahydrofolate deformylase activity|purine nucleotide biosynthetic process|10-formyltetrahydrofolate biosynthetic process|hydrolase activity|metabolic process|one-carbon metabolic process|biosynthetic process|'de novo' IMP biosynthetic process|amino acid binding	Glyoxylate and dicarboxylate metabolism|One carbon pool by folate	1.029669891	UP
7278755	HAPS_RS09275	prevent-host-death protein	biological_process|molecular_function	-	2.428107311	UP
25120022	HAPS_RS11325	calcium-binding domain-containing protein	pathogenesis|proteolysis|metalloendopeptidase activity|extracellular region|zinc ion binding|calcium ion binding	Cationic antimicrobial peptide (CAMP) resistance	1.046577024	UP

25120002	HAPS_RS11225	hypothetical protein	collagen trimer|extracellular matrix structural constituent	-	1.074522385	UP
7277247	HAPS_RS11075	DNA adenine methylase	DNA repair|site-specific DNA-methyltransferase (adenine-specific) activity|DNA methylation	Mismatch repair	1.231996529	UP
7277675	HAPS_RS08565	restriction endonuclease	molecular_function	-	1.414293677	UP
7278290	HAPS_RS03770	hypothetical protein	-	-	2.246165087	UP
23375321	HAPS_RS01570	hypothetical protein	FMN binding|oxidoreductase activity|oxidation-reduction process	-	1.932827995	UP
7277255	HAPS_RS11115	secretin	Type IV pili-dependent localized adherence to host	-	1.136018094	UP
23375366	-	-	-	-	1.71287826	UP
23375554	-	-	molecular_function|biological_process|cellular_component	-	1.053215677	UP
7276925	HAPS_RS01815	IS110 family transposase	transposase activity|transposition, DNA-mediated	-	Inf	UP
23375358	HAPS_RS02890	phage tail protein/putative Fels-1 prophage host specificity protein	provirus|viral process	-	1.534513314	UP
7277127	HAPS_RS05140	lipopolysaccharide N-acetylmannosaminouronosyltransferase	transferase activity|transferase activity, transferring hexosyl groups|transferase activity, transferring glycosyl groups|biosynthetic process|enterobacterial common antigen biosynthetic process|lipopolysaccharide N-acetylmannosaminouronosyltransferase activity	-	1.094134955	UP
7276710	HAPS_RS02780	heme-binding protein	provirus	-	1.027976779	UP
23375376	-	-	-	-	Inf	UP
23375362	HAPS_RS03135	hypothetical protein	-	-	4.920595232	UP
7276839	HAPS_RS03355	hypothetical protein	membrane|integral component of plasma membrane|integral component of membrane	-	1.247131175	UP
23375410	HAPS_RS05645	hypothetical protein	-	-	3.74499632	UP
7278514	HAPS_RS02515	hypothetical protein	-	-	-1.032894321	DOWN

7278130	HAPS_RS09780	guanylate kinase	identical protein binding|cytoplasm|kinase activity|ATP binding|transferase activity|nucleotide phosphorylation|phosphorylation|purine nucleotide metabolic process|nucleotide binding|guanylate kinase activity	Metabolic pathways|Purine metabolism	1.142609344	UP
23375381	HAPS_RS04020	hypothetical protein	-	-	1.149545451	UP
7278726	-	-	molecular_function|biological_process|cellular_component	-	3.749896084	UP
7277008	HAPS_RS04180	formamidopyrimidine-DNA glycosylase	oxidized purine nucleobase lesion DNA N-glycosylase activity|DNA repair	Base excision repair	1.063345625	UP
7277074	HAPS_RS04485	hypothetical protein	-	-	1.817782232	UP
23375562	HAPS_RS11130	phosphatidylglycerophosphatase A	phospholipid catabolic process|phosphatidylglycerophosphatase activity	Metabolic pathways|Glycerophospholipid metabolism	2.207617426	UP
7276693	HAPS_RS02690	bacteriophage P2 Tail completion protein GPR	-	-	2.018194756	UP
23375501	HAPS_RS08995	LysR protein	negative regulation of secondary metabolite biosynthetic process|negative regulation of proteolysis|negative regulation of protein secretion	-	2.637747985	UP
25120017	HAPS_RS11300	PP-loop family protein	tRNA modification|ligase activity, forming carbon-nitrogen bonds	-	1.220310452	UP
7277911	-	-	-	-	1.810878434	UP
23375330	-	-	transposition, DNA-mediated|transposase activity	-	3.237265899	UP
7276708	HAPS_RS02770	antirepressor	cellular_component|molecular_function|biological_process	-	Inf	UP
7278809	HAPS_RS09540	tRNA (uridine(54)-C5)-methyltransferase TrmA	methylation|methyltransferase activity|tRNA binding|RNA processing|tRNA processing|RNA methyltransferase activity|transferase activity|rRNA binding|S-adenosylmethionine-dependent tRNA (m5U54)	-	1.002651343	UP

			methyltransferase activity|tRNA methylation			
23375368	HAPS_RS03700	hypothetical protein	-	-	3.053147122	UP
7276729	HAPS_RS02855	single-stranded DNA-binding protein	DNA repair|DNA replication|single-stranded DNA binding|DNA recombination	Homologous recombination|Mismatch repair|DNA replication	1.271964585	UP
23375470	-	-	growth of symbiont in host	-	2.464577475	UP
23375353	HAPS_RS02720	transposase	-	-	Inf	UP
7276694	HAPS_RS02695	phage virion morphogenesis protein	-	-	1.742068383	UP
7278546	-	-	cellular_component|biological_process|molecular_function	-	3.84787844	UP
23375403	-	-	-	-	1.649474772	UP
7278273	HAPS_RS03695	membrane protein	biological_process|molecular_function	-	1.018426078	UP
7276775	HAPS_RS03065	antirepressor	molecular_function|biological_process|cellular_component	-	2.59781149	UP
23375498	HAPS_RS08925	MULTISPECIES: hypothetical protein	-	-	Inf	UP
7277217	-	-	biological_process|molecular_function	-	1.650060171	UP
7278715	HAPS_RS09085	hypothetical protein	biological_process|molecular_function|cellular_component	-	1.737432435	UP
7278904	-	-	-	-	-2.248622813	DOWN
7276738	HAPS_RS02900	hypothetical protein	-	-	Inf	UP
7277457	HAPS_RS01335	toxin	negative regulation by symbiont of host cell cycle	-	1.693301938	UP
7278150	HAPS_RS09870	transcriptional regulator	regulation of transcription, DNA-templated|sequence-specific DNA binding transcription factor activity	-	1.024346463	UP
7278799	HAPS_RS09490	metal-dependent hydrolase	cellular_component|molecular_function|biological_process	-	1.408945986	UP
23375294	HAPS_RS00310	ABC transporter family protein	ATPase activity, coupled to transmembrane movement of substances|transport	ABC transporters	1.39107403	UP
23375496	-	-	-	-	3.195285556	UP

7277586	HAPS_RS08120	membrane protein	cellular_component|molecular_function|biological_process	-	1.084301862	UP
23375559	-	-	integral component of plasma membrane|transport|transmembrane transport|integral component of membrane|transporter activity|intrinsic component of plasma membrane|phosphorelay signal transduction system|carbohydrate transport|plasma membrane|membrane	Two-component system	1.389424125	UP
7278050	HAPS_RS06110	permease	biological_process|molecular_function	-	1.037817714	UP
7277387	HAPS_RS09750	peptidase	-	-	2.823762625	UP
7276726	HAPS_RS02840	hypothetical protein	-	-	2.049896591	UP
23375442	HAPS_RS07240	hypothetical protein	molecular_function|biological_process|cellular_component	Cationic antimicrobial peptide (CAMP) resistance	1.42549959	UP
7277782	HAPS_RS10880	metallophosphoesterase	protein serine/threonine phosphatase activity|protein dephosphorylation	-	1.090698825	UP
7277388	HAPS_RS10150	IS110 family transposase	transposition, DNA-mediated|transposase activity	-	1.699950238	UP
7277205	-	-	-	-	-1.508886835	DOWN
7277095	HAPS_RS04990	transposase	transposition, DNA-mediated|transposase activity	-	1.85472818	UP
7278643	ilvH	acetolactate synthase small subunit	cellular amino acid biosynthetic process|acetolactate synthase activity|valine biosynthetic process|transferase activity|amino acid binding|isoleucine biosynthetic process|branched-chain amino acid biosynthetic process|metabolic process	Metabolic pathways|Biosynthesis of secondary metabolites|Biosynthesis of antibiotics|Biosynthesis of amino acids|2-Oxocarboxylic acid metabolism|Pantothenate and CoA biosynthesis|Valine, leucine and isoleucine biosynthesis|Butanoate metabolism|C5-Branched dibasic acid metabolism	1.234375171	UP

23375295	HAPS_RS00315	hypothetical protein	-	ABC transporters	1.770068195	UP
23375407	HAPS_RS05415	hypothetical protein	-	-	2.289457955	UP
7276765	HAPS_RS03025	hypothetical protein	-	-	3.920458966	UP
7276803	HAPS_RS03190	lysine exporter protein LysE/YggA	transport|transporter activity	-	1.124450228	UP
7277289	HAPS_RS01535	hypothetical protein	-	-	1.59171855	UP
7278716	HAPS_RS09090	gluconate permease	amino acid transmembrane transport|cellular response to DNA damage stimulus|D-serine transmembrane transporter activity|gluconate transmembrane transport|transport|integral component of plasma membrane|integral component of membrane|D-serine transport|gluconate transmembrane transporter activity|membrane|plasma membrane	-	1.425682187	UP
23375352	HAPS_RS02660	hypothetical protein	-	-	4.002340766	UP
23375413	-	-	-	-	1.450235292	UP
7277132	HAPS_RS05165	arginine transporter permease subunit ArtQ	amine transport|transport|transporter activity|integral component of membrane|plasma membrane|metabolic process|membrane|amino acid transmembrane transport|amino acid transport|ATP catabolic process|polar-amino acid-transporting ATPase activity	ABC transporters	1.038011522	UP
7278711	HAPS_RS09065	oxidoreductase	phosphogluconate dehydrogenase (decarboxylating) activity|coenzyme binding|valine metabolic process|pentose-phosphate shunt|oxidoreductase activity, acting on the CH-OH group of donors, NAD or NADP as acceptor|oxidation-reduction process|oxidoreductase activity|3-hydroxyisobutyrate dehydrogenase activity	-	1.784275982	UP
7277700	HAPS_RS08710	gluconate permease	gluconate transmembrane transporter activity|gluconate	-	1.305001714	UP

			transport			
23375385	-	-	cellular_component|biological_process|molecular_function	-	3.723146683	UP
7276709	HAPS_RS02775	transcriptional regulator	regulation of transcription, DNA-templated|provirus|sequence-specific DNA binding transcription factor activity	-	2.315493026	UP
7277795	HAPS_RS10945	phosphonate ABC transporter permease	organic phosphonate transmembrane transporter activity|alkylphosphonate transport|alkylphosphonate transmembrane transporter activity|transmembrane transport|integral component of membrane|transporter activity|integral component of plasma membrane|transport|organic phosphonate transport|plasma membrane|membrane	ABC transporters	1.32942046	UP
23375471	-	-	oxidoreductase activity|cellular response to DNA damage stimulus|oxidation-reduction process	-	1.426128337	UP
7276882	HAPS_RS00385	molecular chaperone DjlA	membrane|chaperone binding|plasma membrane|integral component of membrane	-	1.001613894	UP
7276784	HAPS_RS03105	hypothetical protein	-	-	2.189588955	UP
7278823	HAPS_RS05335	membrane protein	transport|ATPase activity, coupled to transmembrane movement of substances	ABC transporters	1.604403572	UP
7278590	-	-	-	-	-1.530245113	DOWN
7278696	HAPS_RS09000	hypothetical protein	-	-	2.423776847	UP
7277010	HAPS_RS04190	transcriptional regulator	regulation of transcription, DNA-templated|provirus|sequence-specific DNA binding transcription factor activity	-	4.770324534	UP
7278592	-	-	-	-	-1.574350556	DOWN
7278822	HAPS_RS05330	manganese transporter	cation transmembrane transporter activity|ATP	ABC transporters	1.138404168	UP

			binding|cation transport|ATPase activity, coupled to transmembrane movement of substances			
7276786	-	-	-	-	Inf	UP
7276703	HAPS_RS02745	antitermination protein	-	-	1.802000026	UP
7277687	HAPS_RS08625	hypothetical protein	-	-	2.150982522	UP
7277880	HAPS_RS04900	transporter	alanine:sodium symporter activity|L-alanine transport	-	1.28843139	UP
23375293	HAPS_RS00020	hypothetical protein	-	-	1.663932235	UP
7276978	-	-	-	-	3.842696194	UP
7278472	HAPS_RS02305	DNA internalization-related competence protein ComEC/Rec2	hydrolase activity|integral component of membrane|establishment of competence for transformation|plasma membrane|metabolic process|membrane	-	1.151883113	UP
7277375	HAPS_RS09700	anthranilate synthase component I	anthranilate synthase activity|tryptophan biosynthetic process	Metabolic pathways|Biosynthesis of secondary metabolites|Biosynthesis of antibiotics|Biosynthesis of amino acids|Quorum sensing|Phenylalanine, tyrosine and tryptophan biosynthesis	1.584442939	UP
7278664	HAPS_RS08855	protease TldD	proteolysis	-	1.738417746	UP
