# Supplementary material for: Comparative transcriptional profiling of tildipirosin-resistant and sensitive Haemophilus parasuis
Source: Sci Rep. 2017 Aug 8;7:7517. doi: 10.1038/s41598-017-07972-5 (PMC5548900; doi:10.1038/s41598-017-07972-5)
Supplement: Supplementary file 2 [file 41598_2017_7972_MOESM2_ESM.pdf]

# Comparative transcriptional profiling of tildipirosin-resistant and sensitive *Haemophilus parasuis*

**Zhixin Lei[ab], Shulin Fu[c], Bing Yang[ab], Qianying Liu[ab], Saeed Ahmed[ab], Lei Xu[c], Jincheng Xiong[ab], Jiyue Cao[ab*], Yinsheng Qiu[c*]**

[a] Veterinary Pharmacology Laboratory, College of Veterinary Medicine, Huazhong Agricultural University, Wuhan, 430070, PR China

[b] National Reference Laboratory of Veterinary Drug Residues and MAO Key Laboratory for Detection of Veterinary Drug Residues, Huazhong Agriculture University, Wuhan, 430070, PR China

[c] School of Animal Science and Nutritional Engineering, Wuhan Polytechnic University, Wuhan 430023, PR China

[*]***Corresponding author:***

Prof. Dr. Ji-yue Cao, Caojiyue@mail.hzau.edu.cn

Prof. Dr. Yinsheng Qiu, qiuyinsheng6405@aliyun.com

Table. 2 The upregulated and downregulated DE genes in GO functional categories analysis

| GO_ID | GO_term | TYPE | UP_GENE | DOWN_GENE | all_diff_gene_in_all_GO | gene_UP_list | gene_DOWN_list |
|---|---|---|---|---|---|---|---|
| GO:0005581 | collagen trimer | cellular_component | 3 | 1 | 321 | 25120002(HAPS_RS11225),25119992(HAPS_RS11175),25120019(HAPS_RS11310), | 25120023(HAPS_RS11330), |
| GO:0000271 | polysaccharide biosynthetic process | biological_process | 6 | 0 | 321 | 7277752(HAPS_RS10735),7276855(HAPS_RS00245),7276856(HAPS_RS00250),7277751(HAPS_RS10730),7276847(HAPS_RS00205),7276854(HAPS_RS00240), | - |
| GO:0003333 | amino acid transmembrane transport | biological_process | 3 | 1 | 321 | 7278716(HAPS_RS09090),7277132(HAPS_RS05165),7278001(HAPS_RS00800), | 7278453(metN), |
| GO:0005201 | extracellular matrix structural constituent | molecular_function | 3 | 1 | 321 | 25120002(HAPS_RS11225),25119992(HAPS_RS11175),25120019(HAPS_RS11310), | 25120023(HAPS_RS11330), |
| GO:0009401 | phosphoenolpyruvate-dependent sugar phosphotransferase system | biological_process | 0 | 6 | 321 | - | 7278033(HAPS_RS00960),7277831(HAPS_RS04655),7278035(HAPS_RS00970),23375418(HAPS_RS06060),7277222(HAPS_RS04905),7278034(HAPS_RS00965), |
| GO:0009152 | purine ribonucleotide biosynthetic process | biological_process | 4 | 0 | 321 | 7278844(HAPS_RS05450),7277356(HAPS_RS09615),7277276(HAPS_RS01460),7277279(guaA), | - |
| GO:000 | lipid A | biological_proces | 4 | 0 | 321 | 7278061(lpxD),7278063(HAPS_RS06175), | - |

| | | | | | | | |
|---|---|---|---|---|---|---|---|
| 9245 | biosynthetic process | s | | | | 7277551(HAPS_RS06855),7276852(HAPS_RS00230), | |
| GO:0006457 | protein folding | biological_process | 2 | 4 | 321 | 7278025(HAPS_RS00920),7278857(HAPS_RS05515), | 7278835(HAPS_RS05395),7278198(HAPS_RS10110),7277560(HAPS_RS07995),7278172(groES), |
| GO:0004803 | transposase activity | molecular_function | 18 | 3 | 321 | 7276925(HAPS_RS01815),7277970(HAPS_RS00645),7276915(HAPS_RS00545),7277212(HAPS_RS07130),7278131(HAPS_RS09785),7277095(HAPS_RS04990),7278873(HAPS_RS05585),7278511(HAPS_RS02500),23375467(-),7277075(HAPS_RS04490),7277685(HAPS_RS08615),7278762(HAPS_RS09305),7276863(HAPS_RS00285),23375514(-),23375330(-),7277388(HAPS_RS10150),7278176(HAPS_RS09995),23375499(-), | 23375307(HAPS_RS01030),25120015(-),7277495(HAPS_RS06575), |
| GO:0006313 | transposition, DNA-mediated | biological_process | 18 | 3 | 321 | 7276925(HAPS_RS01815),7277970(HAPS_RS00645),7276915(HAPS_RS00545),7277212(HAPS_RS07130),7278131(HAPS_RS09785),7277095(HAPS_RS04990),7278873(HAPS_RS05585),7278511(HAPS_RS02500),23375467(-),7277075(HAPS_RS04490),7277685(HAPS_RS08615),7278762(HAPS_RS09305),7276863(HAPS_RS00285),23375514(-),23375330(-),7277388(HAPS_ | 23375307(HAPS_RS01030),25120015(-),7277495(HAPS_RS06575), |

| | | | | | | | |
|---|---|---|---|---|---|---|---|
| | | | | | | RS10150),7278176(HAPS_RS09995),23375499(-), | |
| GO:0006865 | amino acid transport | biological_process | 2 | 2 | 321 | 7277259(HAPS_RS11135),7277132(HAPS_RS05165), | 7277005(metQ),7278453(metN), |
| GO:0019843 | rRNA binding | molecular_function | 9 | 1 | 321 | 7278420(HAPS_RS07795),7278417(HAPS_RS07780),7278426(HAPS_RS07825),7278423(HAPS_RS07810),7278424(HAPS_RS07815),7278938(HAPS_RS05895),7278422(HAPS_RS07805),7278418(rplD),7278809(HAPS_RS09540), | 7277094(rpmE), |
| GO:0006184 | GTP catabolic process | biological_process | 4 | 0 | 321 | 7276676(HAPS_RS01015),7278814(fusA),7278896(tuf),7278938(HAPS_RS05895), | - |
| GO:0009408 | response to heat | biological_process | 1 | 3 | 321 | 7278938(HAPS_RS05895), | 7278835(HAPS_RS05395),7278973(HAPS_RS06065),7278172(groES), |
| GO:0034219 | carbohydrate transmembrane transport | biological_process | 2 | 3 | 321 | 7277126(-),7276855(HAPS_RS00245), | 7278033(HAPS_RS00960),7278035(HAPS_RS00970),23375418(HAPS_RS06060), |
| GO:0022625 | cytosolic large ribosomal subunit | cellular_component | 5 | 1 | 321 | 7278420(HAPS_RS07795),7278417(HAPS_RS07780),7278424(HAPS_RS07815),7278422(HAPS_RS07805),7278418(rplD), | 7277094(rpmE), |
| GO:0003924 | GTPase activity | molecular_function | 4 | 0 | 321 | 7276676(HAPS_RS01015),7278814(fusA),7278896(tuf),7278938(HAPS_RS05895), | - |
| GO:0005622 | intracellular | cellular_component | 7 | 1 | 321 | 7278420(HAPS_RS07795),7278423(HAPS_RS07810),7278424(HAPS_RS07815),7276676(HAPS_RS01015),7278814(fusA),727 | 7278037(HAPS_RS00980), |

| | | | | | | | |
|---|---|---|---|---|---|---|---|
| | | | | | | 8896(tuf),7278422(HAPS_RS07805), | |
| GO:0000049 | tRNA binding | molecular_function | 4 | 1 | 321 | 7278424(HAPS_RS07815),7278907(HAPS_RS05750),7278809(HAPS_RS09540),7277000(HAPS_RS02175), | 7276816(HAPS_RS03250), |
| GO:0003735 | structural constituent of ribosome | molecular_function | 11 | 4 | 321 | 7278420(HAPS_RS07795),7278417(HAPS_RS07780),7278416(rpsJ),7278426(HAPS_RS07825),7278423(HAPS_RS07810),7278419(HAPS_RS07790),7278424(HAPS_RS07815),7278421(HAPS_RS07800),7278422(HAPS_RS07805),7278418(rplD),7278425(HAPS_RS07820), | 7278741(rpmH),7278166(rpmG),7278921(HAPS_RS05815),7277094(rpmE), |
| GO:0003723 | RNA binding | molecular_function | 15 | 3 | 321 | 7278420(HAPS_RS07795),7278417(HAPS_RS07780),7278426(HAPS_RS07825),7278043(nusA),7278423(HAPS_RS07810),7277800(rluD),7278424(HAPS_RS07815),7278479(HAPS_RS02340),7277379(HAPS_RS09715),7278907(HAPS_RS05750),7278365(HAPS_RS04150),7278342(rho),7278422(HAPS_RS07805),7278418(rplD),7277000(HAPS_RS02175), | 7277669(rnhB),7278469(HAPS_RS02290),7277094(rpmE), |
| GO:0005215 | transporter activity | molecular_function | 11 | 0 | 321 | 7277126(-),7277869(HAPS_RS04845),7277504(HAPS_RS06620),7277503(HAPS_RS06615),7277871(HAPS_RS04855),7277132(HAPS_RS05165),7278190(panF),7277795(HAPS_RS10945),7276803(HAPS_RS03190),23375559(-),7278677(HAPS_RS089 | - |

| | | | | | | | |
|---|---|---|---|---|---|---|---|
| | | | | | | 15), | |
| GO:0008643 | carbohydrate transport | biological_process | 2 | 3 | 321 | 7276855(HAPS_RS00245),23375559(-), | 7278033(HAPS_RS00960),23375418(HAPS_RS06060),7278034(HAPS_RS00965), |
| GO:0003700 | sequence-specific DNA binding transcription factor activity | molecular_function | 10 | 1 | 321 | 7277084(HAPS_RS04945),23375497(-),7277702(HAPS_RS08720),7276709(HAPS_RS02775),7278150(HAPS_RS09870),7278043(nusA),7278671(HAPS_RS08885),7278678(HAPS_RS08920),7277010(HAPS_RS04190),23375326(HAPS_RS01830), | 7278037(HAPS_RS00980), |
| GO:0030529 | ribonucleoprotein complex | cellular_component | 7 | 2 | 321 | 7278420(HAPS_RS07795),7278417(HAPS_RS07780),7278426(HAPS_RS07825),7278423(HAPS_RS07810),7278424(HAPS_RS07815),7278422(HAPS_RS07805),7278418(rplD), | 7278921(HAPS_RS05815),7277094(rpmE), |
| GO:0046677 | response to antibiotic | biological_process | 7 | 0 | 321 | 7278061(lpxD),7278426(HAPS_RS07825),7277780(HAPS_RS10870),7278896(tuf),7276852(HAPS_RS00230),7278422(HAPS_RS07805),7278418(rplD), | - |
| GO:0005840 | ribosome | cellular_component | 7 | 2 | 321 | 7278420(HAPS_RS07795),7278417(HAPS_RS07780),7278426(HAPS_RS07825),7278423(HAPS_RS07810),7278424(HAPS_RS07815),7278422(HAPS_RS07805),7278418(rplD), | 7278921(HAPS_RS05815),7277094(rpmE), |
| GO:0003674 | molecular_function | molecular_function | 48 | 15 | 321 | 7276708(HAPS_RS02770),7278891(HAPS_RS05675),7278799(HAPS_RS09490),233 | 23375502(-),7277047(HAPS_RS04360),7276957(HAPS_RS01 |

| | | | | | | | |
|---|---|---|---|---|---|---|---|
| | | | | | | 75296(-),7277583(HAPS_RS08105),7278753(HAPS_RS09265),23375310(HAPS_RS01205),7276731(HAPS_RS02865),7277860(-),23375424(-),25120000(-),7276853(HAPS_RS00235),7278273(HAPS_RS03695),7278546(-),7278049(HAPS_RS06105),7278632(HAPS_RS00085),23375385(-),7278766(HAPS_RS09325),7278894(HAPS_RS05690),7277217(-),7276850(HAPS_RS00220),7278683(HAPS_RS08945),7278380(HAPS_RS07620),7276775(HAPS_RS03065),7278507(HAPS_RS02480),7278715(HAPS_RS09085),7278670(HAPS_RS08880),7278726(-),7277910(HAPS_RS07320),7276929(HAPS_RS01835),7278351(HAPS_RS04080),23375386(HAPS_RS04250),7276974(HAPS_RS02055),7277433(HAPS_RS01225),7277675(HAPS_RS08565),7278208(HAPS_RS10560),7278846(HAPS_RS05460),7277586(HAPS_RS08120),7276847(HAPS_RS00205),7277266(HAPS_RS02615),7277723(HAPS_RS10590),23375442(HAPS_RS07240),7278578(HAPS_RS10375),23375554(-),7278259(HAPS_RS03625),7278050(HAPS_RS06110),7278755(HAPS_RS09275),7276947(HAPS_RS01925), | 970),7277038(HAPS_RS04315),7276916(HAPS_RS00550),7277209(HAPS_RS07115),7277899(HAPS_RS07265),7277441(HAPS_RS01260),7278019(HAPS_RS00890),7277875(HAPS_RS04875),7278345(hslO),7277023(HAPS_RS04240),7277443(HAPS_RS01270),23375546(-),7277018(HAPS_RS04220), |

| | | | | | | | |
|---|---|---|---|---|---|---|---|
| GO:0016757 | transferase activity, transferring glycosyl groups | molecular_function | 6 | 0 | 321 | 7277085(HAPS_RS04950),7277752(HAPS_RS10735),7277211(HAPS_RS07125),7277127(HAPS_RS05140),7277751(HAPS_RS10730),7276851(HAPS_RS00225), | - |
| GO:0016853 | isomerase activity | molecular_function | 5 | 3 | 321 | 7277780(HAPS_RS10870),7277800(rluD),7278857(HAPS_RS05515),7277379(HAPS_RS09715),7276987(HAPS_RS02115), | 7278941(HAPS_RS05910),7278198(HAPS_RS10110),7278036(HAPS_RS00975), |
| GO:0006629 | lipid metabolic process | biological_process | 5 | 1 | 321 | 7278061(lpxD),7278087(HAPS_RS06285),7278063(HAPS_RS06175),7278053(HAPS_RS06125),7278057(HAPS_RS06145), | 7277921(HAPS_RS07375), |
| GO:0006810 | transport | biological_process | 26 | 7 | 321 | 7277126(-),7278260(HAPS_RS03630),7278823(HAPS_RS05335),7277869(HAPS_RS04845),7278716(HAPS_RS09090),7277504(HAPS_RS06620),7278863(HAPS_RS05545),23375294(HAPS_RS00310),7276855(HAPS_RS00245),7277503(HAPS_RS06615),7277871(HAPS_RS04855),7277259(HAPS_RS11135),7277873(HAPS_RS04865),7278319(HAPS_RS03920),7276924(HAPS_RS00590),7277246(HAPS_RS11070),7277132(HAPS_RS05165),7278190(panF),7278637(HAPS_RS00110),7277795(HAPS_RS10945),7276803(HAPS_RS03190),7277411(HAPS_RS01125),7278840(HAPS_RS05430),23375559(-),7278677(HAPS_RS08915),7278001(HAPS_RS00800), | 7278438(HAPS_RS07890),7278033(HAPS_RS00960),23375418(HAPS_RS06060),7278452(HAPS_RS02205),7278034(HAPS_RS00965),7277005(metQ),7278453(metN), |

| | | | | | | | |
|---|---|---|---|---|---|---|---|
| GO:0005575 | cellular_component | cellular_component | 30 | 13 | 321 | 7276708(HAPS_RS02770),7278891(HAPS_RS05675),7278799(HAPS_RS09490),7277583(HAPS_RS08105),23375310(HAPS_RS01205),7277860(-),25120000(-),7278546(-),23375385(-),7278766(HAPS_RS09325),7278894(HAPS_RS05690),7276850(HAPS_RS00220),7278683(HAPS_RS08945),7276775(HAPS_RS03065),7278507(HAPS_RS02480),7278715(HAPS_RS09085),7278670(HAPS_RS08880),7278726(-),7277910(HAPS_RS07320),7276929(HAPS_RS01835),7278351(HAPS_RS04080),23375386(HAPS_RS04250),7276974(HAPS_RS02055),7277433(HAPS_RS01225),7278208(HAPS_RS10560),7277586(HAPS_RS08120),23375442(HAPS_RS07240),7278578(HAPS_RS10375),23375554(-),7278259(HAPS_RS03625), | 7277047(HAPS_RS04360),7276957(HAPS_RS01970),7277038(HAPS_RS04315),7276916(HAPS_RS00550),7277209(HAPS_RS07115),7277441(HAPS_RS01260),7278019(HAPS_RS00890),7277875(HAPS_RS04875),7278345(hslO),7277023(HAPS_RS04240),7278612(HAPS_RS10540),7277443(HAPS_RS01270),7277018(HAPS_RS04220), |
| GO:0055085 | transmembrane transport | biological_process | 8 | 2 | 321 | 7277126(-),7276855(HAPS_RS00245),7278319(HAPS_RS03920),7278190(panF),7277795(HAPS_RS10945),7277411(HAPS_RS01125),23375559(-),7278001(HAPS_RS00800), | 7278033(HAPS_RS00960),7278453(metN), |
| GO:0008150 | biological_process | biological_process | 44 | 16 | 321 | 7276708(HAPS_RS02770),7278891(HAPS_RS05675),7278799(HAPS_RS09490),23375296(-),7277583(HAPS_RS08105),23375 | 23375502(-),7277047(HAPS_RS04360),7276957(HAPS_RS01970),7277038(HAPS_RS04315) |

| | | | | | | | |
|---|---|---|---|---|---|---|---|
| | | | | | | 310(HAPS_RS01205),7277860(-),23375424(-),25120000(-),7276853(HAPS_RS00235),7278273(HAPS_RS03695),7278546(-),7278049(HAPS_RS06105),7278632(HAPS_RS00085),7277653(HAPS_RS08450),23375385(-),7278766(HAPS_RS09325),7278894(HAPS_RS05690),7277217(-),7276850(HAPS_RS00220),7278683(HAPS_RS08945),7278380(HAPS_RS07620),7276775(HAPS_RS03065),7278507(HAPS_RS02480),7278715(HAPS_RS09085),7278670(HAPS_RS08880),7278726(-),7277910(HAPS_RS07320),7276929(HAPS_RS01835),7278351(HAPS_RS04080),23375386(HAPS_RS04250),7276974(HAPS_RS02055),7277433(HAPS_RS01225),7278208(HAPS_RS10560),7278846(HAPS_RS05460),7277586(HAPS_RS08120),7277723(HAPS_RS10590),23375442(HAPS_RS07240),7278639(HAPS_RS00120),7278578(HAPS_RS10375),23375554(-),7278259(HAPS_RS03625),7278050(HAPS_RS06110),7278755(HAPS_RS09275), | ,7276916(HAPS_RS00550),7277209(HAPS_RS07115),7277899(HAPS_RS07265),7277441(HAPS_RS01260),7277291(HAPS_RS01545),7278019(HAPS_RS00890),7277875(HAPS_RS04875),7277985(HAPS_RS00720),7278345(hslO),7277023(HAPS_RS04240),7277443(HAPS_RS01270),7277018(HAPS_RS04220), |
| GO:0042626 | ATPase activity, coupled to transmembrane | molecular_function | 7 | 2 | 321 | 7278260(HAPS_RS03630),7278823(HAPS_RS05335),7278822(HAPS_RS05330),7278863(HAPS_RS05545),23375294(HAPS_ | 7277442(HAPS_RS01265),7278452(HAPS_RS02205), |

| | | | | | | | |
|---|---|---|---|---|---|---|---|
| | movement of substances | | | | | RS00310),7277411(HAPS_RS01125),7278840(HAPS_RS05430), | |
| GO:0016021 | integral component of membrane | cellular_component | 27 | 5 | 321 | 7277126(-),7278716(HAPS_RS09090),7276839(HAPS_RS03355),7277511(ubiB),7276855(HAPS_RS00245),7277871(HAPS_RS04855),7277259(HAPS_RS11135),7278191(HAPS_RS10075),7278319(HAPS_RS03920),7276924(HAPS_RS00590),7277132(HAPS_RS05165),7278857(HAPS_RS05515),7278472(HAPS_RS02305),7278190(panF),7277795(HAPS_RS10945),7278023(HAPS_RS00910),7277411(HAPS_RS01125),23375342(HAPS_RS02415),7277551(HAPS_RS06855),23375559(-),7278300(ubiA),7278623(HAPS_RS00040),7278053(HAPS_RS06125),7276882(HAPS_RS00385),7276857(HAPS_RS00255),7278001(HAPS_RS00800),7278057(HAPS_RS06145), | 7278033(HAPS_RS00960),7278325(HAPS_RS03950),23375418(HAPS_RS06060),7278034(HAPS_RS00965),7277921(HAPS_RS07375), |
| GO:0006355 | regulation of transcription, DNA-templated | biological_process | 15 | 2 | 321 | 7277084(HAPS_RS04945),23375497(-),7277702(HAPS_RS08720),7276709(HAPS_RS02775),7278150(HAPS_RS09870),7278043(nusA),7278671(HAPS_RS08885),7276948(HAPS_RS01930),7278014(HAPS_RS00865),7278678(HAPS_RS08920),7278676(HAPS_RS08910),7278342(rho),7278418(rplD),7277010(HAPS_RS04190),233753 | 7277508(fis),7278037(HAPS_RS00980), |

| | | | | | | | |
|---|---|---|---|---|---|---|---|
| | | | | | | 26(HAPS_RS01830), | |
| GO:0019038 | provirus | cellular_component | 5 | 0 | 321 | 23375358(HAPS_RS02890),7278338(HAPS_RS04015),7276709(HAPS_RS02775),7276710(HAPS_RS02780),7277010(HAPS_RS04190), | - |
| GO:0008033 | tRNA processing | biological_process | 6 | 0 | 321 | 7278478(HAPS_RS02335),7278907(HAPS_RS05750),7278917(HAPS_RS05795),7277304(HAPS_RS01600),7278809(HAPS_RS09540),7277000(HAPS_RS02175), | - |
| GO:0009103 | lipopolysaccharide biosynthetic process | biological_process | 4 | 0 | 321 | 7277085(HAPS_RS04950),7277113(glmM),7277551(HAPS_RS06855),7276857(HAPS_RS00255), | - |
| GO:0006811 | ion transport | biological_process | 3 | 1 | 321 | 7276855(HAPS_RS00245),7278190(panF),7278001(HAPS_RS00800), | 7278438(HAPS_RS07890), |
| GO:0009058 | biosynthetic process | biological_process | 4 | 0 | 321 | 7278844(HAPS_RS05450),23375497(-),7277127(HAPS_RS05140),7277551(HAPS_RS06855), | - |
| GO:0006412 | translation | biological_process | 15 | 3 | 321 | 7278420(HAPS_RS07795),7278042(HAPS_RS01005),7278417(HAPS_RS07780),7278416(rpsJ),7278426(HAPS_RS07825),7278423(HAPS_RS07810),7278419(HAPS_RS07790),7278424(HAPS_RS07815),7276676(HAPS_RS01015),7278814(fusA),7278896(tuf),7278421(HAPS_RS07800),7278422(HAPS_RS07805),7278418(rplD),7278425(HAPS_RS07820), | 7278166(rpmG),7278921(HAPS_RS05815),7277094(rpmE), |

| | | | | | | | |
|---|---|---|---|---|---|---|---|
| GO:0016746 | transferase activity, transferring acyl groups | molecular_function | 4 | 0 | 321 | 7278061(lpxD),23375506(-),7278063(HAPS_RS06175),7277000(HAPS_RS02175), | - |
| GO:0005886 | plasma membrane | cellular_component | 27 | 11 | 321 | 7277126(-),7278716(HAPS_RS09090),7277511(ubiB),7277211(HAPS_RS07125),7277871(HAPS_RS04855),7277259(HAPS_RS11135),7278191(HAPS_RS10075),7278319(HAPS_RS03920),7276924(HAPS_RS00590),7277132(HAPS_RS05165),7278857(HAPS_RS05515),7278472(HAPS_RS02305),7278190(panF),7277795(HAPS_RS10945),7278023(HAPS_RS00910),7277411(HAPS_RS01125),23375342(HAPS_RS02415),7277551(HAPS_RS06855),7278896(tuf),23375559(-),7278300(ubiA),7278623(HAPS_RS00040),7278053(HAPS_RS06125),7276882(HAPS_RS00385),7276857(HAPS_RS00255),7278001(HAPS_RS00800),7278057(HAPS_RS06145), | 7278438(HAPS_RS07890),7278835(HAPS_RS05395),7276818(HAPS_RS03255),7278033(HAPS_RS00960),7278325(HAPS_RS03950),23375418(HAPS_RS06060),7278034(HAPS_RS00965),7277005(metQ),7276876(HAPS_RS00355),7278453(metN),7277921(HAPS_RS07375), |
