# Supplementary material for: Comparative transcriptional profiling of tildipirosin-resistant and sensitive Haemophilus parasuis
Source: Sci Rep. 2017 Aug 8;7:7517. doi: 10.1038/s41598-017-07972-5 (PMC5548900; doi:10.1038/s41598-017-07972-5)
Supplement: Supplementary file 3 [file 41598_2017_7972_MOESM3_ESM.pdf]

Comparative transcriptional profiling of tildipirosin-resistant and sensitive *Haemophilus parasuis*

Zhixin Lei[ab], Shulin Fu[c], Bing Yang[ab], Qianying Liu[ab], Saeed Ahmed[ab], Lei Xu[c], Jincheng Xiong[ab], Jiyue Cao[ab*], Yinsheng Qiu[c*]

[a] Veterinary Pharmacology Laboratory, College of Veterinary Medicine, Huazhong Agricultural University, Wuhan, 430070, PR China

[b] National Reference Laboratory of Veterinary Drug Residues and MAO Key Laboratory for Detection of Veterinary Drug Residues, Huazhong Agriculture University, Wuhan, 430070, PR China

[c] School of Animal Science and Nutritional Engineering, Wuhan Polytechnic University, Wuhan 430023, PR China

[*] ***Corresponding author:***

Prof. Dr. Ji-yue Cao, Caojiyue@mail.hzau.edu.cn

Prof. Dr. Yinsheng Qiu, qiuyinsheng6405@aliyun.com

Table. 3 The upregulated and downregulated DE genes in KEGG pathway classification analysis

pathway_ID	PATHWAY_DES	UP_GENES	DOWN_GENES	all_gene_in_all_pathway	gene_UP_list	gene_DOWN_list
ko02060	Phosphotransferase system (PTS)	0	6	884	-	7278035(HAPS_RS00970),7277831(HAPS_RS04655),7277222(HAPS_RS04905),23375418(HAPS_RS06060),7278033(HAPS_RS00960),7278034(HAPS_RS00965),
ko05206	MicroRNAs in cancer	2	0	884	7278764(HAPS_RS09315), 7278765(HAPS_RS09320),	-
ko00561	Glycerolipid metabolism	1	1	884	7278087(HAPS_RS06285),	7277921(HAPS_RS07375),
ko00564	Glycerophospholipid metabolism	4	1	884	23375562(HAPS_RS11130),7278057(HAPS_RS06145),7278053(HAPS_RS06125),7278087(HAPS_RS06285),	7277921(HAPS_RS07375),
ko00052	Galactose metabolism	1	2	884	23375561(HAPS_RS10920),	7278174(HAPS_RS09985),7278032(HAPS_RS00955),
ko00520	Amino sugar and nucleotide sugar metabolism	3	7	884	7277113(glmM),7277081(HAPS_RS04930),7278254(HAPS_RS03600),	7278035(HAPS_RS00970),7277831(HAPS_RS04655),7278174(HAPS_RS09985),23375418(HAPS_RS06060),7278033(HAPS_RS00960),7278941(HAPS_RS05910),7278034(HAPS_RS00965),
ko03010	Ribosome	11	4	884	7278420(HAPS_RS07795), 7278417(HAPS_RS07780), 7278418(rplD),7278421(HAPS_RS07800),7278425(H	7277094(rpmE),7278921(HAPS_RS05815),7278741(rpmH),7278166(rpmG),

					APS_RS07820),7278419(HAPS_RS07790),7278416(rpsJ),7278422(HAPS_RS07805),7278424(HAPS_RS07815),7278423(HAPS_RS07810),7278426(HAPS_RS07825),	
ko00730	Thiamine metabolism	2	0	884	7278685(HAPS_RS08950), 7278686(HAPS_RS08955),	-
ko00562	Inositol phosphate metabolism	2	0	884	7277937(HAPS_RS07450), 7277936(HAPS_RS07445),	-
ko01501	beta-Lactam resistance	3	0	884	7277869(HAPS_RS04845), 7277081(HAPS_RS04930), 7277871(HAPS_RS04855),	-
ko03018	RNA degradation	2	1	884	7278479(HAPS_RS02340), 7278342(rho),	7278735(dnaK),
ko00480	Glutathione metabolism	2	0	884	23375506(-),7278348(HAPS_RS04065),	-
ko00670	One carbon pool by folate	1	1	884	7278844(HAPS_RS05450),	7277241(metF),
ko01503	Cationic antimicrobial peptide (CAMP) resistance	3	0	884	23375442(HAPS_RS07240),25120022(HAPS_RS11325),7278063(HAPS_RS06175),	-
ko00650	Butanoate metabolism	2	0	884	7278643(ilvH),7278623(HAPS_RS00040),	-
ko03430	Mismatch repair	4	0	884	7276729(HAPS_RS02855),	-

					7277216(HAPS_RS07150), 7277247(HAPS_RS11075), 7277553(dnaE),	
ko03030	DNA replication	2	1	884	7276729(HAPS_RS02855), 7277553(dnaE),	7277669(rnhB),
ko00030	Pentose phosphate pathway	2	1	884	7278352(HAPS_RS04085), 7278348(HAPS_RS04065),	7278444(HAPS_RS07920),
ko02024	Quorum sensing	6	1	884	7278840(HAPS_RS05430), 7278841(HAPS_RS05435), 7277375(HAPS_RS09700), 7277869(HAPS_RS04845), 7278839(HAPS_RS05425), 7277871(HAPS_RS04855),	7278663(HAPS_RS08850),
ko00540	Lipopolysaccharide biosynthesis	4	0	884	7277085(HAPS_RS04950), 7277551(HAPS_RS06855), 7278063(HAPS_RS06175), 7278061(lpxD),	-
ko00270	Cysteine and methionine metabolism	2	2	884	7278764(HAPS_RS09315), 7278765(HAPS_RS09320),	7277492(HAPS_RS06560),7278505(HAPS_RS02470),
ko00051	Fructose and mannose metabolism	0	3	884	-	7278035(HAPS_RS00970),7278033(HAPS_RS00960),7278034(HAPS_RS00965),
ko02020	Two-component system	2	2	884	23375559(-),7278623(HAPS_RS00040),	7278189(HAPS_RS10065),7278469(HAPS_RS02290),
ko00290	Valine, leucine and isoleucine biosynthesis	2	0	884	7278643(ilvH),7277114(HAPS_RS05080),	-
ko00500	Starch and sucrose	0	2	884	-	7277831(HAPS_RS04655),7277222(HAPS_RS0

	metabolism					4905),
ko02010	ABC transporters	11	5	884	7277795(HAPS_RS10945), 7278259(HAPS_RS03625), 7278823(HAPS_RS05335), 7278822(HAPS_RS05330), 7278260(HAPS_RS03630), 23375295(HAPS_RS00315),7277411(HAPS_RS01125),7277869(HAPS_RS04845),23375294(HAPS_RS00310),7277132(HAPS_RS05165),7277871(HAPS_RS04855),	7278453(metN),23375476(HAPS_RS08310),7278452(HAPS_RS02205),7277005(metQ),7277899(HAPS_RS07265),
ko00061	Fatty acid biosynthesis	2	0	884	7278681(HAPS_RS08935), 7278369(fabG),	-
ko00230	Purine metabolism	7	0	884	7276794(apaH),7278891(HAPS_RS05675),7278130(HAPS_RS09780),7277553(dnaE),7277276(HAPS_RS01460),7277356(HAPS_RS09615),7277211(HAPS_RS07125),	-
ko00260	Glycine, serine and threonine metabolism	1	0	884	7277114(HAPS_RS05080),	-
ko00430	Taurine and hypotaurine metabolism	1	0	884	23375506(-),	-

ko00300	Lysine biosynthesis	1	0	884	7277214(HAPS_RS07140),	-
ko05133	Pertussis	1	0	884	23375305(HAPS_RS00745),	-
ko00450	Selenocompound metabolism	0	1	884	-	7277492(HAPS_RS06560),
ko00910	Nitrogen metabolism	0	1	884	-	7278542(HAPS_RS10200),
ko03070	Bacterial secretion system	0	1	884	-	7278663(HAPS_RS08850),
ko04917	Prolactin signaling pathway	0	1	884	-	7278174(HAPS_RS09985),
ko00660	C5-Branched dibasic acid metabolism	1	0	884	7278643(ilvH),	-
ko03060	Protein export	0	1	884	-	7278663(HAPS_RS08850),
ko00195	Photosynthesis	0	1	884	-	7278438(HAPS_RS07890),
ko00330	Arginine and proline metabolism	1	0	884	7278578(HAPS_RS10375),	-
ko04112	Cell cycle	1	0	884	7276924(HAPS_RS00590),	-
ko00630	Glyoxylate and dicarboxylate metabolism	1	0	884	7278844(HAPS_RS05450),	-
ko01210	2-Oxocarboxylic acid metabolism	1	0	884	7278643(ilvH),	-
ko00780	Biotin metabolism	1	0	884	7278369(fabG),	-
ko04212	Longevity regulating pathway	0	1	884	-	7278735(dnaK),
ko00790	Folate biosynthesis	1	0	884	7278893(HAPS_RS05685),	-
ko00920	Sulfur metabolism	1	0	884	23375341(HAPS_RS02410),	-

ko00983	Drug metabolism	1	0	884	7277276(HAPS_RS01460),	-
ko03420	Nucleotide excision repair	1	0	884	7278326(uvrC),	-
ko00860	Porphyrin and chlorophyll metabolism	1	0	884	7277425(HAPS_RS01195),	-
ko00020	Citrate cycle (TCA cycle)	1	0	884	7278623(HAPS_RS00040),	-
ko00770	Pantothenate and CoA biosynthesis	1	0	884	7278643(ilvH),	-
ko00250	Alanine, aspartate and glutamate metabolism	0	1	884	-	7278542(HAPS_RS10200),
ko03410	Base excision repair	1	0	884	7277008(HAPS_RS04180),	-
ko01040	Biosynthesis of unsaturated fatty acids	1	0	884	7278369(fabG),	-
ko00400	Phenylalanine, tyrosine and tryptophan biosynthesis	1	0	884	7277375(HAPS_RS09700),	-
ko00970	Aminoacyl-tRNA biosynthesis	1	0	884	7276896(cysS),	-
ko00130	Ubiquinone and other terpenoid-quinone biosynthesis	1	0	884	7278300(ubiA),	-
ko01100	Metabolic pathways	42	10	884	23375562(HAPS_RS11130),7278057(HAPS_RS06145),23375506(-),7278643(ilvH),7277114(HAPS_RS05080),7278687(HAPS_RS08960),7278155(HAPS_RS098	7278035(HAPS_RS00970),7278542(HAPS_RS10200),7277241(metF),7278438(HAPS_RS07890),7278174(HAPS_RS09985),7277921(HAPS_RS07375),7278033(HAPS_RS00960),7277492(HAPS_RS06560),7278505(HAPS_RS02470),7278034(HAPS_RS00965),

					95),7278685(HAPS_RS08950),7278053(HAPS_RS06125),7277085(HAPS_RS04950),7277083(upp),7277938(HAPS_RS07455),7278686(HAPS_RS08955),7277937(HAPS_RS07450),7278352(HAPS_RS04085),7278891(HAPS_RS05675),7278623(HAPS_RS00040),7278764(HAPS_RS09315),7277214(HAPS_RS07140),7277279(guaA),7278681(HAPS_RS08935),7277551(HAPS_RS06855),7277375(HAPS_RS09700),7278300(ubiA),7278130(HAPS_RS09780),7278765(HAPS_RS09320),7278348(HAPS_RS04065),7277936(HAPS_RS07445),7277113(glmM),7277425(HAPS_RS01195),7277081(HAPS_RS04930),7278063(HAPS_RS06175),23375561(HAPS_RS10920),7278578(HAPS_RS10375),7278061(lpxD	

					),7277553(dnaE),7277276(HAPS_RS01460),7277356(HAPS_RS09615),7278087(HAPS_RS06285),7278254(HAPS_RS03600),7278369(fabG),7277211(HAPS_RS07125),	
ko00190	Oxidative phosphorylation	1	1	884	7278623(HAPS_RS00040),	7278438(HAPS_RS07890),
ko01212	Fatty acid metabolism	2	0	884	7278681(HAPS_RS08935), 7278369(fabG),	-
ko00620	Pyruvate metabolism	2	1	884	7278623(HAPS_RS00040), 7278055(HAPS_RS06135),	7278612(HAPS_RS10540),
ko00010	Glycolysis / Gluconeogenesis	0	2	884	-	7277831(HAPS_RS04655),23375418(HAPS_RS06060),
ko00720	Carbon fixation pathways in prokaryotes	1	1	884	7278623(HAPS_RS00040),	7277241(metF),
ko01110	Biosynthesis of secondary metabolites	14	4	884	7278057(HAPS_RS06145), 7278643(ilvH),7277114(HAPS_RS05080),7278352(HAPS_RS04085),7278623(HAPS_RS00040),7277214(HAPS_RS07140),7277375(HAPS_RS09700),7278300(ubiA),7278348(HAPS_RS04065),7277425(HAPS_RS01	7278542(HAPS_RS10200),7277921(HAPS_RS07375),7277492(HAPS_RS06560),7278505(HAPS_RS02470),

					195),7277276(HAPS_RS01460),7277356(HAPS_RS09615),7278087(HAPS_RS06285),7277211(HAPS_RS07125),	
ko01120	Microbial metabolism in diverse environments	7	2	884	7277938(HAPS_RS07455), 7277937(HAPS_RS07450), 7278352(HAPS_RS04085), 7278623(HAPS_RS00040), 7277214(HAPS_RS07140), 7278348(HAPS_RS04065), 7277936(HAPS_RS07445),	7278542(HAPS_RS10200),7277241(metF),
ko00240	Pyrimidine metabolism	4	0	884	7278155(HAPS_RS09895), 7277083(upp),7278891(HAPS_RS05675),7277553(dnaE),	-
ko01230	Biosynthesis of amino acids	4	3	884	7278643(ilvH),7277114(HAPS_RS05080),7277214(HAPS_RS07140),7277375(HAPS_RS09700),	7278542(HAPS_RS10200),7277492(HAPS_RS06560),7278505(HAPS_RS02470),
ko01130	Biosynthesis of antibiotics	10	1	884	7278643(ilvH),7277114(HAPS_RS05080),7277938(HAPS_RS07455),7278352(HAPS_RS04085),7278623(HAPS_RS00040),7277214(HAPS_RS07140),7277375(H	7278542(HAPS_RS10200),

					APS_RS09700),7278348(HAPS_RS04065),7277113(glmM),7277356(HAPS_RS09615),	
ko03440	Homologous recombination	2	0	884	7276729(HAPS_RS02855), 7277553(dnaE),	-
ko01200	Carbon metabolism	4	1	884	7277114(HAPS_RS05080), 7278352(HAPS_RS04085), 7278623(HAPS_RS00040), 7278348(HAPS_RS04065),	7277241(metF),
