# Supplementary material for: Comparative transcriptional profiling of tildipirosin-resistant and sensitive Haemophilus parasuis
Source: Sci Rep. 2017 Aug 8;7:7517. doi: 10.1038/s41598-017-07972-5 (PMC5548900; doi:10.1038/s41598-017-07972-5)
Supplement: Supplementary file 4 [file 41598_2017_7972_MOESM4_ESM.pdf]

# Comparative transcriptional profiling of tildipirosin-resistant and sensitive *Haemophilus parasuis*

**Zhixin Lei[ab], Shulin Fu[c], Bing Yang[ab], Qianying Liu[ab], Saeed Ahmed[ab], Lei Xu[c], Jincheng Xiong[ab], Jiyue Cao[ab*], Yinsheng Qiu[c*]**

[a] Veterinary Pharmacology Laboratory, College of Veterinary Medicine, Huazhong Agricultural University, Wuhan, 430070, PR China

[b] National Reference Laboratory of Veterinary Drug Residues and MAO Key Laboratory for Detection of Veterinary Drug Residues, Huazhong Agriculture University, Wuhan, 430070, PR China

[c] School of Animal Science and Nutritional Engineering, Wuhan Polytechnic University, Wuhan 430023, PR China

[*]***Corresponding author:***

Prof. Dr. Ji-yue Cao, Caojiyue@mail.hzau.edu.cn

Prof. Dr. Yinsheng Qiu, qiuyinsheng6405@aliyun.com

Table. 4 The upregulated and downregulated DE genes of JS32 compared with JS0135 in Metabolic pathway.

| gene id | gene name | String_symble | description | log2FC | updown |
|---|---|---|---|---|---|
| 7278765 | HAPS_RS09320 | HAPS_1919 | DNA cytosine methyltransferase | Inf | UP |
| 7276735 | HAPS_RS02885 | HAPS_0586 | hypothetical protein | Inf | UP |
| 23375380 | HAPS_RS03985 | | transposase, partial | Inf | UP |
| 7278764 | HAPS_RS09315 | HAPS_1918 | restriction endonuclease subunit M | Inf | UP |
| 7276852 | HAPS_RS00230 | wbgX | spore coat protein | Inf | UP |
| 7278673 | HAPS_RS08895 | HAPS_1827 | site-specific DNA-methyltransferase | Inf | UP |
| 7278672 | HAPS_RS08890 | HAPS_1826 | type III restriction-modification system EcoPI enzyme subunit res | Inf | UP |
| 7278763 | HAPS_RS09310 | hgaI | type II restriction endonuclease HgAI | Inf | UP |
| 7276974 | HAPS_RS02055 | HAPS_0415 | chromosome segregation ATPase | Inf | UP |
| 7278766 | HAPS_RS09325 | HAPS_1920 | transcriptional regulator | Inf | UP |
| 7278680 | HAPS_RS08930 | HAPS_1834 | DUF305 domain-containing protein | Inf | UP |
| 7276848 | HAPS_RS00210 | lsgB | CMP-N-acetylneuraminate-beta-galactosamide-alpha-2, 3-sialyltransferase | Inf | UP |
| 7278297 | HAPS_RS03810 | HAPS_0782 | hypothetical protein | Inf | UP |
| 7276850 | HAPS_RS00220 | wcwK | glycosyl transferase | Inf | UP |
| 7278682 | HAPS_RS08940 | HAPS_1836 | methionine sulfoxide reductase | Inf | UP |
| 7276849 | HAPS_RS00215 | HAPS_0043 | hypothetical protein | Inf | UP |
| 7278338 | HAPS_RS04015 | HAPS_0823 | preprotein translocase | Inf | UP |
| 7276734 | HAPS_RS02880 | HAPS_0585 | hypothetical protein | 5.838693486 | UP |
| 7276851 | HAPS_RS00225 | wcfQ | glycosyl transferase | Inf | UP |
| 7278208 | HAPS_RS10560 | HAPS_2174 | transcriptional regulator | 9.704690558 | UP |

| | | | | | |
|---|---|---|---|---|---|
| 7276854 | HAPS_RS00240 | capD | polysaccharide biosynthesis protein CapD | Inf | UP |
| 7278750 | HAPS_RS09250 | HAPS_1904 | transcriptional regulator | Inf | UP |
| 23375326 | HAPS_RS01830 | | hypothetical protein | 6.454452277 | UP |
| 7278674 | HAPS_RS08900 | HAPS_1828 | ATPase (AAA+ superfamily) protein | Inf | UP |
| 7276853 | HAPS_RS00235 | wbgY | glycosyl transferase | Inf | UP |
| 7278337 | HAPS_RS04010 | HAPS_0822 | transcriptional regulator | Inf | UP |
| 7277433 | HAPS_RS01225 | HAPS_0246 | hypothetical protein | Inf | UP |
| 7276929 | HAPS_RS01835 | HAPS_0370 | hypothetical protein | 5.110947532 | UP |
| 7278671 | HAPS_RS08885 | HAPS_1825 | transcriptional regulator | Inf | UP |
| 7278678 | HAPS_RS08920 | HAPS_1832 | transcriptional regulator | Inf | UP |
| 23375379 | HAPS_RS03980 | | MULTISPECIES: hypothetical protein, partial | Inf | UP |
| 7278676 | HAPS_RS08910 | HAPS_1830 | MerR family transcriptional regulator | Inf | UP |
| 23375304 | HAPS_RS00740 | | hypothetical protein | 4.186471477 | UP |
| 7278035 | HAPS_RS00970 | ptsEIIB | PTS mannose transporter subunit IIAB | -2.599575471 | DOWN |
| 7278762 | HAPS_RS09305 | HAPS_1916 | DDE transposase | Inf | UP |
| 7277472 | - | | - | -3.721064565 | DOWN |
| 7277936 | HAPS_RS07445 | iolD | 3D-(3,5/4)-trihydroxycyclohexane-1,2-dione acylhydrolase (decyclizing) | 4.303853938 | UP |
| 7278335 | HAPS_RS04000 | HAPS_0820 | hypothetical protein | Inf | UP |
| 23375341 | HAPS_RS02410 | | hypothetical protein | Inf | UP |
| 7278810 | - | | - | -3.665528435 | DOWN |
| 7278033 | HAPS_RS00960 | ptsEIID | PTS fructose transporter subunit IID | -2.630006793 | DOWN |
| 7278034 | HAPS_RS00965 | ptsEIIC | PTS fructose transporter subunit IIC | -2.33608009 | DOWN |
| 23375305 | HAPS_RS00745 | | hypothetical protein | 3.191135334 | UP |
| 7278683 | HAPS_RS08945 | HAPS_1837 | MULTISPECIES: hypothetical protein | Inf | UP |

| | | | | | |
|---|---|---|---|---|---|
| 7278037 | HAPS_RS00980 | agaR | DeoR family transcriptional regulator | -2.399428365 | DOWN |
| 7278125 | - | | - | -4.284654209 | DOWN |
| 7277583 | HAPS_RS08105 | HAPS_1670 | transposase | Inf | UP |
| 7278871 | HAPS_RS05575 | int | integrase | 2.929602194 | UP |
| 7277222 | HAPS_RS04905 | ptsB | PTS sucrose transporter subunit IIBC | -2.552407786 | DOWN |
| 7277414 | HAPS_RS01140 | | sugar ABC transporter substrate-binding protein | -1.849192963 | DOWN |
| 7277937 | HAPS_RS07450 | iolE | myo-inosose-2 dehydratase | 2.675045058 | UP |
| 7277669 | rnhB | rnhB | ribonuclease HII | -1.832547092 | DOWN |
| 7276695 | HAPS_RS02700 | | hypothetical protein | Inf | UP |
| 23375440 | HAPS_RS07230 | | hypothetical protein | Inf | UP |
| 7277084 | HAPS_RS04945 | ilvY | transcriptional regulator IlvY | 2.289202683 | UP |
| 7278811 | - | | - | -2.924620532 | DOWN |
| 7278036 | HAPS_RS00975 | agaS | tagatose-6-phosphate ketose isomerase | -1.772358261 | DOWN |
| 23375314 | HAPS_RS01405 | | hypothetical protein | -2.053383027 | DOWN |
| 7278453 | metN | metN | D-methionine ABC transporter, ATP-binding protein | -1.506498993 | DOWN |
| 7277452 | HAPS_RS01315 | | NAD(P)H-dependent oxidoreductase | -1.694393168 | DOWN |
| 7277495 | HAPS_RS06575 | HAPS_1357 | DDE transposase | -2.781873899 | DOWN |
| 7278379 | HAPS_RS07615 | cjrC | colicin Js receptor | Inf | UP |
| 7278424 | HAPS_RS07815 | rplP | MULTISPECIES: 50S ribosomal protein L16 | 2.157934836 | UP |
| 7278753 | HAPS_RS09265 | HAPS_1907 | DNA uptake Rossmann fold nucleotide-binding protein | Inf | UP |
| 7277899 | HAPS_RS07265 | | biotin transporter BioY | -1.698839064 | DOWN |
| 7277685 | HAPS_RS08615 | HAPS_1772 | MULTISPECIES: integrase, partial | 5.644000208 | UP |
| 7278469 | HAPS_RS02290 | csrA | carbon storage regulator | -1.800127114 | DOWN |
| 7278922 | HAPS_RS05820 | | hypothetical protein | -1.77171914 | DOWN |
| 7278438 | HAPS_RS07890 | atpC | F0F1 ATP synthase subunit epsilon | -2.984968884 | DOWN |

| | | | | | |
|---|---|---|---|---|---|
| 7278681 | HAPS_RS08935 | HAPS_1835 | diguanylate cyclase | Inf | UP |
| 7278246 | HAPS_RS03560 | | hypothetical protein | -1.920244436 | DOWN |
| 7276766 | HAPS_RS03030 | HAPS_0615 | hypothetical protein | Inf | UP |
| 7278032 | HAPS_RS00955 | bgaC | beta-galactosidase | -1.40626703 | DOWN |
| 25120000 | - | | - | Inf | UP |
| 7278840 | HAPS_RS05430 | oppCD | ABC transporter | 2.062121604 | UP |
| 7277476 | - | | - | -3.343406487 | DOWN |
| 7278334 | HAPS_RS03995 | HAPS_0819 | hypothetical protein | Inf | UP |
| 7276680 | HAPS_RS02625 | HAPS_0531 | hypothetical protein | 2.106597431 | UP |
| 23375386 | HAPS_RS04250 | | transcriptional regulator | 1.971845738 | UP |
| 25119994 | HAPS_RS11185 | HAPS_0349 | restriction endonuclease subunit S | 2.072210552 | UP |
| 7277560 | HAPS_RS07995 | grpE | nucleotide exchange factor GrpE | -1.66978547 | DOWN |
| 7278533 | HAPS_RS02610 | HAPS_0528 | hypothetical protein | 1.920624235 | UP |
| 7278819 | HAPS_RS05315 | infA | MULTISPECIES: translation initiation factor IF-1 | -2.201028312 | DOWN |
| 7278971 | HAPS_RS06055 | sfsA | sugar fermentation stimulation protein SfsA | 1.89514219 | UP |
| 7278841 | HAPS_RS05435 | HAPS_1117 | ABC transporter permease | 1.952413852 | UP |
| 23375560 | HAPS_RS10725 | | polysaccharide biosynthesis protein | 6.598409601 | UP |
| 7277209 | HAPS_RS07115 | | hypothetical protein | -1.38310727 | DOWN |
| 7277241 | metF | metF | 5,10-methylenetetrahydrofolate reductase | -1.296638212 | DOWN |
| 7278422 | HAPS_RS07805 | rplV | MULTISPECIES: 50S ribosomal protein L22 | 1.893095211 | UP |
| 7278423 | HAPS_RS07810 | rpsC | 30S ribosomal protein S3 | 1.927744951 | UP |
| 7277502 | HAPS_RS06610 | HAPS_1364 | cell envelope protein TonB | 2.123051769 | UP |
| 23375342 | HAPS_RS02415 | | hypothetical protein | 4.130966859 | UP |
| 7277561 | HAPS_RS08000 | HAPS_1648 | hypothetical protein | -1.842362909 | DOWN |
| 7277442 | HAPS_RS01265 | HAPS_0255 | ABC transporter ATP-binding protein | -1.964921991 | DOWN |

| | | | | | |
|---|---|---|---|---|---|
| 25119992 | HAPS_RS11175 | | hypothetical protein | 3.363081732 | UP |
| 25120023 | HAPS_RS11330 | | hypothetical protein | -1.460771024 | DOWN |
| 7278663 | HAPS_RS08850 | secE | preprotein translocase subunit SecE | -1.396834435 | DOWN |
| 7278356 | HAPS_RS04105 | | tRNA 5-methoxyuridine(34)/uridine 5-oxyacetic acid(34) synthase CmoB | -1.554116427 | DOWN |
| 7278347 | - | | - | -2.26124672 | DOWN |
| 7278419 | HAPS_RS07790 | rplW | MULTISPECIES: 50S ribosomal protein L23 | 1.780584045 | UP |
| 7278505 | HAPS_RS02470 | metK | S-adenosylmethionine synthase | -1.262569701 | DOWN |
| 7277659 | - | | - | -2.61267293 | DOWN |
| 25120015 | - | | - | -3.672924454 | DOWN |
| 7277082 | HAPS_RS04935 | HAPS_1014 | ATP-dependent helicase | 1.758325282 | UP |
| 7278418 | rplD | rplD | 50S ribosomal protein L4 | 1.875752748 | UP |
| 7277266 | HAPS_RS02615 | ccdA | cytochrome C biogenesis protein CcdA | 1.944093839 | UP |
| 7277492 | HAPS_RS06560 | metE | 5-methyltetrahydropteroyltriglutamate--homocysteine S-methyltransferase | -1.509862246 | DOWN |
| 23375557 | - | | - | 7.306123217 | UP |
| 7276679 | HAPS_RS02620 | HAPS_0530 | peptide-methionine (R)-S-oxide reductase | 1.775356644 | UP |
| 7277864 | HAPS_RS04820 | | hypothetical protein | -2.937405903 | DOWN |
| 7277443 | HAPS_RS01270 | HAPS_0256 | dehydrogenase | -2.155789631 | DOWN |
| 7276916 | HAPS_RS00550 | mraZ | division/cell wall cluster transcriptional repressor MraZ | -1.301021645 | DOWN |
| 7278189 | HAPS_RS10065 | HAPS_2074 | C4-dicarboxylate ABC transporter | -1.123513198 | DOWN |
| 7278129 | HAPS_RS09775 | radA | DNA repair protein RadA | -1.198892602 | DOWN |
| 25120019 | HAPS_RS11310 | | hypothetical protein | 4.570704623 | UP |
| 7278172 | groES | groS | molecular chaperone GroES | -1.672288981 | DOWN |
| 7277921 | HAPS_RS07375 | plsY | glycerol-3-phosphate acyltransferase | -1.315245742 | DOWN |

| | | | | | |
|---|---|---|---|---|---|
| 7278087 | HAPS_RS06285 | plsX | phosphate acyltransferase | 1.763728025 | UP |
| 23375310 | HAPS_RS01205 | | bacteriophage protein | Inf | UP |
| 7278333 | HAPS_RS03990 | HAPS_0818 | hypothetical protein | Inf | UP |
| 7278421 | HAPS_RS07800 | rpsS | MULTISPECIES: 30S ribosomal protein S19 | 1.776533259 | UP |
| 7278896 | tuf | tuf | elongation factor Tu | 1.881710636 | UP |
| 7278814 | fusA | fusA | elongation factor G | 1.778705511 | UP |
| 23375418 | HAPS_RS06060 | | hypothetical protein | -1.556622016 | DOWN |
| 7278631 | HAPS_RS00080 | HAPS_0016 | hypothetical protein | Inf | UP |
| 7276816 | HAPS_RS03250 | HAPS_0665 | hypothetical protein | -2.759595383 | DOWN |
| 7278941 | HAPS_RS05910 | nanE | N-acetylmannosamine-6-phosphate 2-epimerase | -1.212099579 | DOWN |
| 7278345 | hslO | hslO | molecular chaperone Hsp33 | -1.514463502 | DOWN |
| 7277672 | HAPS_RS08550 | HAPS_1759 | DNA-binding protein | -1.08712377 | DOWN |
| 7277094 | rpmE | rpmE | 50S ribosomal protein L31 | -1.04860361 | DOWN |
| 7278417 | HAPS_RS07780 | rplC | 50S ribosomal protein L3 | 1.676961795 | UP |
| 7277081 | HAPS_RS04930 | nagZ | beta-hexosaminidase | 2.007952895 | UP |
| 7277441 | HAPS_RS01260 | HAPS_0254 | ABC transporter substrate-binding protein | -1.452132428 | DOWN |
| 7276948 | HAPS_RS01930 | HAPS_0389 | transcriptional regulator | 8.009512211 | UP |
| 7277039 | HAPS_RS04320 | HAPS_0888 | hypothetical protein | Inf | UP |
| 7278326 | uvrC | uvrC | excinuclease ABC subunit C | 1.59023168 | UP |
| 7278325 | HAPS_RS03950 | HAPS_0810 | protease HtpX | -1.286369482 | DOWN |
| 23375345 | HAPS_RS02560 | | hypothetical protein | 2.679742762 | UP |
| 7278174 | HAPS_RS09985 | HAPS_2059 | galactose-1-phosphate uridylyltransferase | -1.228100027 | DOWN |
| 7277985 | HAPS_RS00720 | | membrane protein | -1.114777619 | DOWN |
| 7278420 | HAPS_RS07795 | rplB | 50S ribosomal protein L2 | 1.726552563 | UP |
| 7277083 | upp | upp | uracil phosphoribosyltransferase | 1.692545386 | UP |

| | | | | | |
|---|---|---|---|---|---|
| 7277216 | HAPS_RS07150 | HAPS_1471 | DNA mismatch repair protein MutS | 1.537570335 | UP |
| 7276876 | HAPS_RS00355 | ybgC | tol-pal system-associated acyl-CoA thioesterase | -1.207215115 | DOWN |
| 7278754 | HAPS_RS09270 | HAPS_1908 | twitching motility protein PilT | 6.634897641 | UP |
| 7278416 | rpsJ | rpsJ | MULTISPECIES: 30S ribosomal protein S10 | 1.579751246 | UP |
| 7278425 | HAPS_RS07820 | rpmC | MULTISPECIES: 50S ribosomal protein L29 | 1.675799119 | UP |
| 7278479 | HAPS_RS02340 | deaD | RNA helicase | 1.539914284 | UP |
| 7276818 | HAPS_RS03255 | HAPS_0667 | hypothetical protein | -1.099796473 | DOWN |
| 7277260 | HAPS_RS11140 | trxA | thiol reductase thioredoxin | -1.521207878 | DOWN |
| 7278444 | HAPS_RS07920 | | ribokinase | -1.004510222 | DOWN |
| 23375546 | - | | - | -1.492873896 | DOWN |
| 7276975 | HAPS_RS02060 | HAPS_0416 | hypothetical protein | Inf | UP |
| 25120020 | HAPS_RS11315 | comFC | putative amidophosphoribosyltransferase | Inf | UP |
| 7278452 | HAPS_RS02205 | metI | methionine ABC transporter permease | -1.105960252 | DOWN |
| 7277075 | HAPS_RS04490 | HAPS_0924 | IS110 family transposase | Inf | UP |
| 7278336 | HAPS_RS04005 | HAPS_0821 | hypothetical protein | Inf | UP |
| 7276947 | HAPS_RS01925 | HAPS_0388 | plasmid maintenance protein ParE | Inf | UP |
| 7278511 | HAPS_RS02500 | | IS110 family transposase | Inf | UP |
| 7277970 | HAPS_RS00645 | | IS110 family transposase | Inf | UP |
| 7277534 | HAPS_RS06770 | | membrane protein | -1.639390925 | DOWN |
| 7277005 | metQ | plpA | membrane protein | -1.1174643 | DOWN |
| 7278612 | HAPS_RS10540 | glo1 | lactoylglutathione lyase | -1.03214369 | DOWN |
| 7277831 | HAPS_RS04655 | crr | PTS glucose transporter subunit IIA | -1.358769999 | DOWN |
| 23375502 | - | | - | -2.107918148 | DOWN |
| 7277850 | HAPS_RS04750 | HAPS_0977 | hypothetical protein | -1.007827124 | DOWN |
| 7276915 | HAPS_RS00545 | | IS110 family transposase | 7.695204273 | UP |

| | | | | | |
|---|---|---|---|---|---|
| 25120010 | HAPS_RS11265 | | site-specific DNA-methyltransferase | 2.007026782 | UP |
| 23375514 | - | | - | Inf | UP |
| 7278198 | HAPS_RS10110 | fklB | peptidyl-prolyl cis-trans isomerase | -1.058690849 | DOWN |
| 7278131 | HAPS_RS09785 | HAPS_2016 | IS110 family transposase | Inf | UP |
| 7278542 | HAPS_RS10200 | gltD | glutamate synthase subunit beta | -1.031793795 | DOWN |
| 7278735 | dnaK | HAPS_1889 | molecular chaperone DnaK | -1.220601416 | DOWN |
| 7278870 | - | | - | -2.738156474 | DOWN |
| 7276961 | HAPS_RS01990 | | antitoxin | -1.44482212 | DOWN |
| 7277754 | HAPS_RS10745 | | cytochrome c-type biogenesis protein CcmE | -1.07284192 | DOWN |
| 23375476 | HAPS_RS08310 | | hypothetical protein | -1.688911224 | DOWN |
| 7278769 | HAPS_RS09340 | | hypothetical protein | Inf | UP |
| 7277356 | HAPS_RS09615 | HAPS_1978 | phosphoribosylformylglycinamidine synthase | 1.511020219 | UP |
| 7277411 | HAPS_RS01125 | cydC | cysteine/glutathione ABC transporter ATP-binding protein/permease CydC | 1.526579839 | UP |
| 25120006 | HAPS_RS11245 | HAPS_0889 | hypothetical protein | Inf | UP |
| 7276957 | HAPS_RS01970 | HAPS_0398 | hypothetical protein | -2.507194183 | DOWN |
| 7278893 | HAPS_RS05685 | HAPS_1169 | anthranilate synthase component II | 1.457116197 | UP |
| 7277508 | fis | fis | Fis family transcriptional regulator | -1.304025647 | DOWN |
| 7278176 | HAPS_RS09995 | HAPS_2061 | integrase | 1.531919528 | UP |
| 7278677 | HAPS_RS08915 | HAPS_1831 | cation transporter | Inf | UP |
| 7277751 | HAPS_RS10730 | lsgE | glycosyl transferase | 1.420384752 | UP |
| 7277503 | HAPS_RS06615 | HAPS_1365 | TonB system transport protein ExbD | 2.0932457 | UP |
| 7277379 | HAPS_RS09715 | HAPS_2001 | 23S rRNA pseudouridylate synthase B | 1.416302737 | UP |
| 25120024 | - | | - | -2.307782679 | DOWN |
| 7278686 | HAPS_RS08955 | thiD | hydroxymethylpyrimidine/phosphomethylpyrimidine kinase | Inf | UP |

| | | | | | |
|---|---|---|---|---|---|
| 23375506 | - | | - | 1.584578524 | UP |
| 7277875 | HAPS_RS04875 | HAPS_1002 | hypothetical protein | -1.007468514 | DOWN |
| 7277752 | HAPS_RS10735 | lsgF | amylovoran biosynthesis protein AmsE | 1.428396394 | UP |
| 7278578 | HAPS_RS10375 | | oxidoreductase | 1.590917489 | UP |
| 7278685 | HAPS_RS08950 | thiE | thiamine phosphate synthase | Inf | UP |
| 7278166 | rpmG | rpmG | MULTISPECIES: 50S ribosomal protein L33 | -1.129883585 | DOWN |
| 7277114 | HAPS_RS05080 | ilvA | PLP-dependent threonine dehydratase | 1.357426547 | UP |
| 7278352 | HAPS_RS04085 | devB | 6-phosphogluconolactonase | 1.343231896 | UP |
| 7278426 | HAPS_RS07825 | rpsQ | 30S ribosomal protein S17 | 1.400550991 | UP |
| 7277330 | HAPS_RS01725 | alxA | type I restriction endonuclease HindVIIP subunit M | 1.302905186 | UP |
| 7277113 | glmM | glmM | phosphoglucosamine mutase | 1.379694839 | UP |
| 23375398 | - | | - | -2.854685455 | DOWN |
| 7277431 | HAPS_RS01215 | HAPS_0244 | hypothetical protein | Inf | UP |
| 7278751 | HAPS_RS09255 | HAPS_1905 | phosphoglycolate phosphatase | Inf | UP |
| 7278835 | HAPS_RS05395 | htpG | molecular chaperone HtpG | -1.014805653 | DOWN |
| 7277801 | - | | - | -2.220454468 | DOWN |
| 7277279 | guaA | guaA | GMP synthetase | 1.291412301 | UP |
| 7278043 | nusA | nusA | transcription termination protein NusA | 1.304137217 | UP |
| 7278478 | HAPS_RS02335 | tadA | tRNA-specific adenosine deaminase | 1.895945922 | UP |
| 7278973 | HAPS_RS06065 | hslV | MULTISPECIES: HslU--HslV peptidase proteolytic subunit | -1.424634974 | DOWN |
| 23375550 | - | | - | 8.391384501 | UP |
| 7277471 | - | | - | -3.711941922 | DOWN |
| 7278380 | HAPS_RS07620 | cjrA | iron-regulated lipoprotein | Inf | UP |
| 7278127 | HAPS_RS09765 | HAPS_2012 | DNA methyltransferase | -1.553556325 | DOWN |
| 7278670 | HAPS_RS08880 | HAPS_1824 | DsbA family protein | Inf | UP |

| | | | | | |
|---|---|---|---|---|---|
| 7278845 | HAPS_RS05455 | hns | DNA-binding protein | -1.086418628 | DOWN |
| 7278190 | panF | panF | sodium/panthothenate symporter | 1.295215737 | UP |
| 7278872 | HAPS_RS05580 | HAPS_1148 | antirepressor | 1.293662491 | UP |
| 23375441 | HAPS_RS07235 | | hypothetical protein | 3.369074664 | UP |
| 7277780 | HAPS_RS10870 | parE | DNA topoisomerase IV subunit B | 1.371212155 | UP |
| 7278874 | HAPS_RS05590 | HAPS_1150 | hypothetical protein | 1.256242643 | UP |
| 7278369 | fabG | fabG | beta-ketoacyl-ACP reductase | 1.175131282 | UP |
| 7277214 | HAPS_RS07140 | lysA | diaminopimelate decarboxylase | 1.329350388 | UP |
| 7277022 | HAPS_RS04235 | HAPS_0871 | hypothetical protein | Inf | UP |
| 7276725 | HAPS_RS02835 | HAPS_0576 | hypothetical protein | 2.074252495 | UP |
| 7278839 | HAPS_RS05425 | HAPS_1115 | ABC transporter ATP-binding protein | 1.307330261 | UP |
| 7276863 | HAPS_RS00285 | | IS110 family transposase | 5.345539731 | UP |
| 23375551 | HAPS_RS10235 | | transposase | 4.716500124 | UP |
| 7278887 | HAPS_RS05655 | HAPS_1163 | ribonucleoside-triphosphate reductase | -1.035149294 | DOWN |
| 7278768 | HAPS_RS09335 | | hypothetical protein | Inf | UP |
| 7276856 | HAPS_RS00250 | ptp | tyrosine phosphatase | 1.223137329 | UP |
| 7277432 | HAPS_RS01220 | HAPS_0245 | hypothetical protein | Inf | UP |
| 7277085 | HAPS_RS04950 | lbgB | lipooligosaccharide D-glycero-D-manno-heptosyltransferase | 1.319723841 | UP |
| 7278938 | HAPS_RS05895 | | GTPase HflX | 1.243443398 | UP |
| 23375516 | HAPS_RS09370 | | hypothetical protein | 1.71049479 | UP |
| 23375467 | - | | - | 6.511675154 | UP |
| 7276730 | HAPS_RS02860 | | hypothetical protein | -1.708255807 | DOWN |
| 7277291 | HAPS_RS01545 | HAPS_0309 | transcriptional regulator | -1.167712614 | DOWN |
| 7277304 | HAPS_RS01600 | HAPS_0322 | tRNA (adenosine(37)-N6)-threonylcarbamoyltransferase complex dimerization subunit type 1 TsaB | 1.439925028 | UP |

| | | | | | |
|---|---|---|---|---|---|
| 7278863 | HAPS_RS05545 | | ABC transporter permease | 2.067228954 | UP |
| 7277038 | HAPS_RS04315 | HAPS_0887 | hypothetical protein | -1.152610189 | DOWN |
| 7278873 | HAPS_RS05585 | HAPS_0842 | transposase | 1.66889389 | UP |
| 7277511 | ubiB | ubiB | ubiquinone biosynthesis protein UbiB | 1.169910598 | UP |
| 7277600 | HAPS_RS08195 | acpP | acyl carrier protein | -1.735364128 | DOWN |
| 7277246 | HAPS_RS11070 | HAPS_2280 | sodium-dependent transporter | 1.883284225 | UP |
| 7276769 | HAPS_RS03035 | | hypothetical protein | -1.772272167 | DOWN |
| 7278057 | HAPS_RS06145 | cdsA | phosphatidate cytidylyltransferase | 1.162025812 | UP |
| 7277873 | HAPS_RS04865 | HAPS_1000 | ABC transporter ATPase | 1.421396648 | UP |
| 7277658 | - | | - | -2.523280226 | DOWN |
| 7278319 | HAPS_RS03920 | hcaT | 3-phenylpropionic acid transporter | 1.165228183 | UP |
| 7276896 | cysS | cysS | cysteine--tRNA ligase | 1.267869049 | UP |
| 7278191 | HAPS_RS10075 | HAPS_2076 | membrane protein | 1.665177326 | UP |
| 7277080 | rumB | rlmC | 23S rRNA (uracil(747)-C(5))-methyltransferase RlmC | 1.217590145 | UP |
| 7277259 | HAPS_RS11135 | rhtB | membrane protein | 1.731031417 | UP |
| 7277938 | HAPS_RS07455 | iolG | inositol 2-dehydrogenase | 1.24949581 | UP |
| 23375296 | - | | - | 1.147948329 | UP |
| 7277910 | HAPS_RS07320 | | membrane protein | 1.26116693 | UP |
| 23375510 | - | | - | 5.816937878 | UP |
| 7277235 | HAPS_RS11020 | HAPS_2269 | hypothetical protein | 1.320854606 | UP |
| 7278894 | HAPS_RS05690 | rmuC | DNA recombination protein RmuC | 1.224214483 | UP |
| 7278907 | HAPS_RS05750 | HAPS_1182 | tRNA(ANN) t(6)A37 threonylcarbamoyladenosine modification protein | 1.124292548 | UP |
| 7277702 | HAPS_RS08720 | | transcriptional regulator | 1.209402561 | UP |
| 7278061 | lpxD | lpxD | UDP-3-O-(3-hydroxymyristoyl)glucosamine N-acyltransferase | 1.219070576 | UP |

| | | | | | |
|---|---|---|---|---|---|
| 7278351 | HAPS_RS04080 | HAPS_0836 | hypothetical protein | 1.130996622 | UP |
| 7278042 | HAPS_RS01005 | rimP | ribosome maturation factor | 1.826979944 | UP |
| 7276793 | - | | - | -2.087064171 | DOWN |
| 7277276 | HAPS_RS01460 | guaB | IMP dehydrogenase | 1.125655003 | UP |
| 23375311 | HAPS_RS01210 | | Mu protein C/ Mor gp17 transcription regulator | Inf | UP |
| 7278891 | HAPS_RS05675 | nrdD | anaerobic ribonucleoside-triphosphate reductase | 1.13314156 | UP |
| 7278014 | HAPS_RS00865 | nrdR | NrdR family transcriptional regulator | 1.176494122 | UP |
| 7277211 | HAPS_RS07125 | gpt | xanthine phosphoribosyltransferase | 2.666628627 | UP |
| 23375561 | HAPS_RS10920 | | beta-D-galactosidase | 1.205718699 | UP |
| 7277869 | HAPS_RS04845 | HAPS_0996 | ABC transporter substrate-binding protein | 1.091938882 | UP |
| 7278637 | HAPS_RS00110 | HAPS_0022 | ABC transporter permease | 1.079378743 | UP |
| 7276728 | HAPS_RS02850 | HAPS_0579 | translocation protein TolB precursor | 1.838755868 | UP |
| 7278741 | rpmH | rpmH | MULTISPECIES: 50S ribosomal protein L34 | -1.078295064 | DOWN |
| 7278023 | HAPS_RS00910 | | BAX inhibitor protein | 1.393648037 | UP |
| 7278121 | - | | - | 1.52645721 | UP |
| 7276720 | HAPS_RS02820 | | hypothetical protein | 2.182917168 | UP |
| 7277800 | rluD | rluD | 23S rRNA pseudouridine(1911/1915/1917) synthase | 1.118745367 | UP |
| 7277212 | HAPS_RS07130 | HAPS_1467 | IS110 family transposase | 3.73510795 | UP |
| 7277551 | HAPS_RS06855 | kdtA | 3-deoxy-D-manno-octulosonic acid transferase | 1.061251634 | UP |
| 7278507 | HAPS_RS02480 | HAPS_0502 | Nif3-like dinuclear metal center hexameric protein | 1.082041732 | UP |
| 7278846 | HAPS_RS05460 | | Na+/H+ antiporter | 1.71176435 | UP |
| 23375497 | - | | - | Inf | UP |
| 7278063 | HAPS_RS06175 | lpxA | acyl | 1.050861878 | UP |
| 7276857 | HAPS_RS00255 | wzz | tyrosine protein kinase | 1.166272544 | UP |
| 7277504 | HAPS_RS06620 | HAPS_1366 | TonB-system energizer ExbB | 2.177624027 | UP |

| | | | | | |
|---|---|---|---|---|---|
| 23375475 | HAPS_RS08305 | | hypothetical protein | 1.894948688 | UP |
| 7277018 | HAPS_RS04220 | HAPS_0867 | integrase | -1.289124756 | DOWN |
| 7277434 | HAPS_RS01230 | HAPS_0247 | hypothetical protein | Inf | UP |
| 7278025 | HAPS_RS00920 | dsbC | protein disulfide-isomerase | 1.16740708 | UP |
| 7277652 | HAPS_RS08445 | hicB | DNA repair protein HhH-GPD | 1.57109199 | UP |
| 7278921 | HAPS_RS05815 | rpsU | MULTISPECIES: 30S ribosomal protein S21 | -1.563858769 | DOWN |
| 7278155 | HAPS_RS09895 | pyrD | dihydroorotate dehydrogenase 2 | 1.338332528 | UP |
| 7277213 | HAPS_RS07135 | HAPS_1468 | transposase | 1.131545332 | UP |
| 7277553 | dnaE | dnaE | DNA polymerase III subunit alpha | 1.143680298 | UP |
| 23375499 | - | | - | Inf | UP |
| 7278687 | HAPS_RS08960 | thiM | hydroxyethylthiazole kinase | Inf | UP |
| 7277298 | HAPS_RS01575 | glnE | bifunctional glutamine synthetase adenylyltransferase/deadenyltransferase | 1.288212171 | UP |
| 7277087 | HAPS_RS04955 | HAPS_1019 | ADP-heptose--LPS heptosyltransferase | 1.217343796 | UP |
| 7276727 | HAPS_RS02845 | HAPS_0578 | recombinase | 1.822591535 | UP |
| 7277126 | - | | - | 2.528404649 | UP |
| 7278857 | HAPS_RS05515 | ppiD | peptidylprolyl isomerase | 1.121745418 | UP |
| 7276724 | HAPS_RS02830 | HAPS_0575 | hypothetical protein | 2.457901143 | UP |
| 23375493 | HAPS_RS08690 | | hypothetical protein | -1.287420445 | DOWN |
| 7278348 | HAPS_RS04065 | gnd | phosphogluconate dehydrogenase (NADP(+)-dependent, decarboxylating) | 1.020261881 | UP |
| 7278342 | rho | rho | transcription termination factor Rho | 1.06071099 | UP |
| 7278106 | - | | - | -2.665273333 | DOWN |
| 7277723 | HAPS_RS10590 | hxuB | hypothetical protein | 1.821726212 | UP |
| 7276676 | HAPS_RS01015 | infB | translation initiation factor IF-2 | 1.012254957 | UP |

| | | | | | |
|---|---|---|---|---|---|
| 23375463 | HAPS_RS08180 | | hypothetical protein | 1.09531219 | UP |
| 7277000 | HAPS_RS02175 | tmcA | tRNA cytosine(34) acetyltransferase TmcA | 1.046484697 | UP |
| 7278639 | HAPS_RS00120 | stbE | RelE toxin | 1.359925558 | UP |
| 7276685 | HAPS_RS02655 | HAPS_0536 | phage capsid scaffolding protein | 3.148295814 | UP |
| 7276987 | HAPS_RS02115 | topB | DNA topoisomerase III | 1.001653974 | UP |
| 7278254 | HAPS_RS03600 | murA | UDP-N-acetylglucosamine 1-carboxyvinyltransferase | 1.063975553 | UP |
| 7278300 | ubiA | ubiA | 4-hydroxybenzoate octaprenyltransferase | 1.010266215 | UP |
| 7278869 | - | | - | -3.195571512 | DOWN |
| 23375424 | - | | - | 1.011636729 | UP |
| 7277653 | HAPS_RS08450 | comM | ATP-dependent protease | 1.58419914 | UP |
| 7278259 | HAPS_RS03625 | HAPS_0744 | ABC transporter permease | 1.231430885 | UP |
| 7277425 | HAPS_RS01195 | hemN1 | YggW family oxidoreductase | 1.022953413 | UP |
| 23375417 | - | | - | 2.731983021 | UP |
| 7277020 | HAPS_RS04230 | HAPS_0869 | IclR family transcriptional regulator | 3.511161788 | UP |
| 23375422 | - | | - | -2.76764252 | DOWN |
| 7278614 | HAPS_RS10550 | HAPS_2172 | hypothetical protein | 1.039555561 | UP |
| 7276731 | HAPS_RS02865 | HAPS_0582 | hypothetical protein | 5.45597025 | UP |
| 7278632 | HAPS_RS00085 | HAPS_0017 | hypothetical protein | 2.724075192 | UP |
| 7276855 | HAPS_RS00245 | wza | sugar transporter | 1.043807665 | UP |
| 7277278 | - | | - | 1.115572067 | UP |
| 7277871 | HAPS_RS04855 | oppC | peptide ABC transporter permease | 1.01657303 | UP |
| 7277427 | HAPS_RS01200 | HAPS_0240 | hypothetical protein | 2.144323913 | UP |
| 7278019 | HAPS_RS00890 | HAPS_0177 | cell division protein ZapB | -1.37228185 | DOWN |
| 7278049 | HAPS_RS06105 | HAPS_1256 | permease | 1.379247589 | UP |
| 7278001 | HAPS_RS00800 | | sodium/glutamate symporter | 3.373882219 | UP |

| | | | | | |
|---|---|---|---|---|---|
| 7278305 | HAPS_RS03850 | | hypothetical protein | 1.139330327 | UP |
| 7278055 | HAPS_RS06135 | gloB | hydroxyacylglutathione hydrolase | 1.050991993 | UP |
| 7278580 | - | | - | -1.703962017 | DOWN |
| 23375542 | HAPS_RS10015 | | hypothetical protein | -1.987869589 | DOWN |
| 23375472 | HAPS_RS08265 | | hypothetical protein | 2.362678189 | UP |
| 23375393 | HAPS_RS04640 | | hypothetical protein | 3.999989343 | UP |
| 7278260 | HAPS_RS03630 | HAPS_0745 | ABC transporter ATP-binding protein | 1.196917148 | UP |
| 7278623 | HAPS_RS00040 | frdD | fumarate reductase | 1.500358976 | UP |
| 23375316 | HAPS_RS01465 | | exopolysaccharide biosynthesis protein | 1.065819094 | UP |
| 7278053 | HAPS_RS06125 | pgpB | phosphatidylglycerophosphatase | 1.029222961 | UP |
| 7278917 | HAPS_RS05795 | HAPS_1192 | tRNA dihydrouridine(20/20a) synthase DusA | 1.136895347 | UP |
| 7278675 | HAPS_RS08905 | | hypothetical protein | Inf | UP |
| 7276847 | HAPS_RS00205 | wzx | Lsg locus protein 1 | 1.045683871 | UP |
| 7276924 | HAPS_RS00590 | ftsW | cell division protein FtsW | 1.024133388 | UP |
| 7277047 | HAPS_RS04360 | | hypothetical protein | -1.358163484 | DOWN |
| 7277440 | HAPS_RS01255 | HAPS_0253 | ABC transporter permease | -1.074995617 | DOWN |
| 7278365 | HAPS_RS04150 | ftsJ | rRNA methyltransferase | 1.060122077 | UP |
| 7277860 | - | | - | Inf | UP |
| 7276809 | HAPS_RS03215 | kefBC | potassium transporter | 1.09092493 | UP |
| 7278292 | HAPS_RS03780 | | terminase | 2.361942275 | UP |
| 7278689 | HAPS_RS08970 | | protease TldD | Inf | UP |
| 23375307 | HAPS_RS01030 | | hypothetical protein | -1.394767921 | DOWN |
| 23375426 | - | | - | Inf | UP |
| 23375375 | HAPS_RS03755 | | predicted ATPase involved in chromosome partitioning | 1.399279576 | UP |
| 7278467 | HAPS_RS02280 | degS | outer membrane-stress sensor serine endopeptidase DegS | 1.155699213 | UP |

| | | | | | |
|---|---|---|---|---|---|
| 7278591 | - | | - | -2.52616732 | DOWN |
| 7278296 | HAPS_RS03805 | HAPS_0781 | transcriptional regulator | 2.061810299 | UP |
| 7276794 | apaH | HAPS_0643 | bis(5\-nucleosyl)-tetraphosphatase (symmetrical) | 1.009395891 | UP |
| 23375555 | HAPS_RS10515 | | hypothetical protein | 1.569903236 | UP |
| 7277023 | HAPS_RS04240 | | hypothetical protein | -1.771236342 | DOWN |
| 7278844 | HAPS_RS05450 | purU | formyltetrahydrofolate deformylase | 1.029669891 | UP |
| 7278755 | HAPS_RS09275 | HAPS_1909 | prevent-host-death protein | 2.428107311 | UP |
| 25120022 | HAPS_RS11325 | | calcium-binding domain-containing protein | 1.046577024 | UP |
| 25120002 | HAPS_RS11225 | | hypothetical protein | 1.074522385 | UP |
| 7277247 | HAPS_RS11075 | HAPS_2281 | DNA adenine methylase | 1.231996529 | UP |
| 7277675 | HAPS_RS08565 | HAPS_1762 | restriction endonuclease | 1.414293677 | UP |
| 7278290 | HAPS_RS03770 | HAPS_0775 | hypothetical protein | 2.246165087 | UP |
| 23375321 | HAPS_RS01570 | | hypothetical protein | 1.932827995 | UP |
| 7277255 | HAPS_RS11115 | comE | secretin | 1.136018094 | UP |
| 23375366 | - | | - | 1.71287826 | UP |
| 23375554 | - | | - | 1.053215677 | UP |
| 7276925 | HAPS_RS01815 | | IS110 family transposase | Inf | UP |
| 23375358 | HAPS_RS02890 | | phage tail protein/putative Fels-1 prophage host specificity protein | 1.534513314 | UP |
| 7277127 | HAPS_RS05140 | wecG | lipopolysaccharide N-acetylmannosaminouronosyltransferase | 1.094134955 | UP |
| 7276710 | HAPS_RS02780 | HAPS_0561 | heme-binding protein | 1.027976779 | UP |
| 23375376 | - | | - | Inf | UP |
| 23375362 | HAPS_RS03135 | | hypothetical protein | 4.920595232 | UP |
| 7276839 | HAPS_RS03355 | HAPS_0688 | hypothetical protein | 1.247131175 | UP |
| 23375410 | HAPS_RS05645 | | hypothetical protein | 3.74499632 | UP |
| 7278514 | HAPS_RS02515 | | hypothetical protein | -1.032894321 | DOWN |

| | | | | | |
|---|---|---|---|---|---|
| 7278130 | HAPS_RS09780 | gmk | guanylate kinase | 1.142609344 | UP |
| 23375381 | HAPS_RS04020 | | hypothetical protein | 1.149545451 | UP |
| 7278726 | - | | - | 3.749896084 | UP |
| 7277008 | HAPS_RS04180 | mutM | formamidopyrimidine-DNA glycosylase | 1.063345625 | UP |
| 7277074 | HAPS_RS04485 | HAPS_0923 | hypothetical protein | 1.817782232 | UP |
| 23375562 | HAPS_RS11130 | | phosphatidylglycerophosphatase A | 2.207617426 | UP |
| 7276693 | HAPS_RS02690 | gpR | bacteriophage P2 Tail completion protein GPR | 2.018194756 | UP |
| 23375501 | HAPS_RS08995 | | LysR protein | 2.637747985 | UP |
| 25120017 | HAPS_RS11300 | | PP-loop family protein | 1.220310452 | UP |
| 7277911 | - | | - | 1.810878434 | UP |
| 23375330 | - | | - | 3.237265899 | UP |
| 7276708 | HAPS_RS02770 | HAPS_0559 | antirepressor | Inf | UP |
| 7278809 | HAPS_RS09540 | trmA | tRNA (uridine(54)-C5)-methyltransferase TrmA | 1.002651343 | UP |
| 23375368 | HAPS_RS03700 | | hypothetical protein | 3.053147122 | UP |
| 7276729 | HAPS_RS02855 | HAPS_0580 | single-stranded DNA-binding protein | 1.271964585 | UP |
| 23375470 | - | | - | 2.464577475 | UP |
| 23375353 | HAPS_RS02720 | | transposase | Inf | UP |
| 7276694 | HAPS_RS02695 | gpS | phage virion morphogenesis protein | 1.742068383 | UP |
| 7278546 | - | | - | 3.84787844 | UP |
| 23375403 | - | | - | 1.649474772 | UP |
| 7278273 | HAPS_RS03695 | HAPS_0758 | membrane protein | 1.018426078 | UP |
| 7276775 | HAPS_RS03065 | HAPS_0624 | antirepressor | 2.59781149 | UP |
| 23375498 | HAPS_RS08925 | | MULTISPECIES: hypothetical protein | Inf | UP |
| 7277217 | - | | - | 1.650060171 | UP |
| 7278715 | HAPS_RS09085 | wbpK | hypothetical protein | 1.737432435 | UP |

| | | | | | |
|---|---|---|---|---|---|
| 7278904 | - | | - | -2.248622813 | DOWN |
| 7276738 | HAPS_RS02900 | | hypothetical protein | Inf | UP |
| 7277457 | HAPS_RS01335 | | toxin | 1.693301938 | UP |
| 7278150 | HAPS_RS09870 | | transcriptional regulator | 1.024346463 | UP |
| 7278799 | HAPS_RS09490 | HAPS_1953 | metal-dependent hydrolase | 1.408945986 | UP |
| 23375294 | HAPS_RS00310 | | ABC transporter family protein | 1.39107403 | UP |
| 23375496 | - | | - | 3.195285556 | UP |
| 7277586 | HAPS_RS08120 | yadS | membrane protein | 1.084301862 | UP |
| 23375559 | - | | - | 1.389424125 | UP |
| 7278050 | HAPS_RS06110 | HAPS_1257 | permease | 1.037817714 | UP |
| 7277387 | HAPS_RS09750 | pilD | peptidase | 2.823762625 | UP |
| 7276726 | HAPS_RS02840 | HAPS_0577 | hypothetical protein | 2.049896591 | UP |
| 23375442 | HAPS_RS07240 | | hypothetical protein | 1.42549959 | UP |
| 7277782 | HAPS_RS10880 | HAPS_2239 | metallophosphoesterase | 1.090698825 | UP |
| 7277388 | HAPS_RS10150 | | IS110 family transposase | 1.699950238 | UP |
| 7277205 | - | | - | -1.508886835 | DOWN |
| 7277095 | HAPS_RS04990 | HAPS_1027 | transposase | 1.85472818 | UP |
| 7278643 | ilvH | ilvH | acetolactate synthase small subunit | 1.234375171 | UP |
| 23375295 | HAPS_RS00315 | | hypothetical protein | 1.770068195 | UP |
| 23375407 | HAPS_RS05415 | | hypothetical protein | 2.289457955 | UP |
| 7276765 | HAPS_RS03025 | HAPS_0614 | hypothetical protein | 3.920458966 | UP |
| 7276803 | HAPS_RS03190 | lysE | lysine exporter protein LysE/YggA | 1.124450228 | UP |
| 7277289 | HAPS_RS01535 | HAPS_0307 | hypothetical protein | 1.59171855 | UP |
| 7278716 | HAPS_RS09090 | HAPS_1870 | gluconate permease | 1.425682187 | UP |
| 23375352 | HAPS_RS02660 | | hypothetical protein | 4.002340766 | UP |

| | | | | | |
|---|---|---|---|---|---|
| 23375413 | - | | - | 1.450235292 | UP |
| 7277132 | HAPS_RS05165 | artQ | arginine transporter permease subunit ArtQ | 1.038011522 | UP |
| 7278711 | HAPS_RS09065 | HAPS_1865 | oxidoreductase | 1.784275982 | UP |
| 7277700 | HAPS_RS08710 | | gluconate permease | 1.305001714 | UP |
| 23375385 | - | | - | 3.723146683 | UP |
| 7276709 | HAPS_RS02775 | HAPS_0560 | transcriptional regulator | 2.315493026 | UP |
| 7277795 | HAPS_RS10945 | HAPS_2252 | phosphonate ABC transporter permease | 1.32942046 | UP |
| 23375471 | - | | - | 1.426128337 | UP |
| 7276882 | HAPS_RS00385 | djlA | molecular chaperone DjlA | 1.001613894 | UP |
| 7276784 | HAPS_RS03105 | | hypothetical protein | 2.189588955 | UP |
| 7278823 | HAPS_RS05335 | yfeC | membrane protein | 1.604403572 | UP |
| 7278590 | - | | - | -1.530245113 | DOWN |
| 7278696 | HAPS_RS09000 | | hypothetical protein | 2.423776847 | UP |
| 7277010 | HAPS_RS04190 | HAPS_0859 | transcriptional regulator | 4.770324534 | UP |
| 7278592 | - | | - | -1.574350556 | DOWN |
| 7278822 | HAPS_RS05330 | yfeB | manganese transporter | 1.138404168 | UP |
| 7276786 | - | | - | Inf | UP |
| 7276703 | HAPS_RS02745 | | antitermination protein | 1.802000026 | UP |
| 7277687 | HAPS_RS08625 | | hypothetical protein | 2.150982522 | UP |
| 7277880 | HAPS_RS04900 | | transporter | 1.28843139 | UP |
| 23375293 | HAPS_RS00020 | | hypothetical protein | 1.663932235 | UP |
| 7276978 | - | | - | 3.842696194 | UP |
| 7278472 | HAPS_RS02305 | rec2 | DNA internalization-related competence protein ComEC/Rec2 | 1.151883113 | UP |
| 7277375 | HAPS_RS09700 | trpE | anthranilate synthase component I | 1.584442939 | UP |
| 7278664 | HAPS_RS08855 | HAPS_1818 | protease TldD | 1.738417746 | UP |
