# Supplementary material for: Comparative transcriptional profiling of tildipirosin-resistant and sensitive Haemophilus parasuis
Source: Sci Rep. 2017 Aug 8;7:7517. doi: 10.1038/s41598-017-07972-5 (PMC5548900; doi:10.1038/s41598-017-07972-5)
Supplement: Supplementary file 5 [file 41598_2017_7972_MOESM5_ESM.pdf]

Comparative transcriptional profiling of tildipirosin-resistant and sensitive *Haemophilus parasuis*

Zhixin Lei[ab], Shulin Fu[c], Bing Yang[ab], Qianying Liu[ab], Saeed Ahmed[ab], Lei Xu[c], Jincheng Xiong[ab], Jiyue Cao[ab*], Yinsheng Qiu[c*]

[a] Veterinary Pharmacology Laboratory, College of Veterinary Medicine, Huazhong Agricultural University, Wuhan, 430070, PR China

[b] National Reference Laboratory of Veterinary Drug Residues and MAO Key Laboratory for Detection of Veterinary Drug Residues, Huazhong Agriculture University, Wuhan, 430070, PR China

[c] School of Animal Science and Nutritional Engineering, Wuhan Polytechnic University, Wuhan 430023, PR China

[*]***Corresponding author:***

Prof. Dr. Ji-yue Cao, Caojiyue@mail.hzau.edu.cn

Prof. Dr. Yinsheng Qiu, qiuyinsheng6405@aliyun.com

Table. 5 The upregulated and downregulated DE genes in ABC transporters pathway

gene id	gene name	String_symble	description	kegg_term	updown	FC
7277411	HAPS_RS01125	cydC	cysteine/glutathione ABC transporter ATP-binding protein/permease CydC	ABC transporters	UP	2.881020325
7277869	HAPS_RS04845	HAPS_0996	ABC transporter substrate-binding protein	ABC transporters|Quorum sensing|beta-Lactam resistance	UP	2.131603167
7278259	HAPS_RS03625	HAPS_0744	ABC transporter permease	ABC transporters	UP	2.34799752
7277871	HAPS_RS04855	oppC	peptide ABC transporter permease	ABC transporters|Quorum sensing|beta-Lactam resistance	UP	2.023107569
7278260	HAPS_RS03630	HAPS_0745	ABC transporter ATP-binding protein	ABC transporters	UP	2.292492713
23375294	HAPS_RS00310		ABC transporter family protein	ABC transporters	UP	2.622738607
23375295	HAPS_RS00315		hypothetical protein	ABC transporters	UP	3.410700785
7277132	HAPS_RS05165	artQ	arginine transporter permease subunit ArtQ	ABC transporters	UP	2.05339549
7277795	HAPS_RS10945	HAPS_2252	phosphonate ABC transporter permease	ABC transporters	UP	2.513017051
7278823	HAPS_RS05335	yfeC	membrane protein	ABC transporters	UP	3.040700183
7278822	HAPS_RS05330	yfeB	manganese transporter	ABC transporters	UP	2.201373843
7278453	metN	metN	D-methionine ABC transporter, ATP-binding protein	ABC transporters	DOWN	0.3519643
7277899	HAPS_RS07265		biotin transporter BioY	ABC transporters	DOWN	0.308033878
7278452	HAPS_RS02205	metI	methionine ABC transporter permease	ABC transporters	DOWN	0.464593137
7277005	metQ	plpA	membrane protein	ABC transporters	DOWN	0.460903204
23375476	HAPS_RS08310		hypothetical protein	ABC transporters	DOWN	0.31016091
