# Supplementary material for: Comparative transcriptional profiling of tildipirosin-resistant and sensitive Haemophilus parasuis
Source: Sci Rep. 2017 Aug 8;7:7517. doi: 10.1038/s41598-017-07972-5 (PMC5548900; doi:10.1038/s41598-017-07972-5)
Supplement: Supplementary file 6 [file 41598_2017_7972_MOESM6_ESM.pdf]

Comparative transcriptional profiling of tildipirosin-resistant and sensitive *Haemophilus parasuis*

Zhixin Lei[ab], Shulin Fu[c], Bing Yang[ab], Qianying Liu[ab], Saeed Ahmed[ab], Lei Xu[c], Jincheng Xiong[ab], Jiyue Cao[ab*], Yinsheng Qiu[c*]

[a] Veterinary Pharmacology Laboratory, College of Veterinary Medicine, Huazhong Agricultural University, Wuhan, 430070, PR China

[b] National Reference Laboratory of Veterinary Drug Residues and MAO Key Laboratory for Detection of Veterinary Drug Residues, Huazhong Agriculture University, Wuhan, 430070, PR China

[c] School of Animal Science and Nutritional Engineering, Wuhan Polytechnic University, Wuhan 430023, PR China

[*]***Corresponding author:***

Prof. Dr. Ji-yue Cao, Caojiyue@mail.hzau.edu.cn

Prof. Dr. Yinsheng Qiu, qiuyinsheng6405@aliyun.com

Table. 6 The upregulated and downregulated DE genes in Ribosome pathway.

gene id	gene name	String_saymble	description	kegg_term	log2FC	updown	FC
7278424	HAPS_RS07815	rplP	MULTISPECIES: 50S ribosomal protein L16	Ribosome	2.157934836	UP	4.462755711
7278422	HAPS_RS07805	rplV	MULTISPECIES: 50S ribosomal protein L22	Ribosome	1.893095211	UP	3.714312528
7278423	HAPS_RS07810	rpsC	30S ribosomal protein S3	Ribosome	1.927744951	UP	3.804600444
7278419	HAPS_RS07790	rplW	MULTISPECIES: 50S ribosomal protein L23	Ribosome	1.780584045	UP	3.435652317
7278418	rplD	rplD	50S ribosomal protein L4	Ribosome	1.875752748	UP	3.669930516
7278421	HAPS_RS07800	rpsS	MULTISPECIES: 30S ribosomal protein S19	Ribosome	1.776533259	UP	3.426019254
7277094	rpmE	rpmE	50S ribosomal protein L31	Ribosome	-1.04860361	DOWN	0.483435857
7278417	HAPS_RS07780	rplC	50S ribosomal protein L3	Ribosome	1.676961795	UP	3.197538643
7278420	HAPS_RS07795	rplB	50S ribosomal protein L2	Ribosome	1.726552563	UP	3.30936074
7278416	rpsJ	rpsJ	MULTISPECIES: 30S ribosomal protein S10	Ribosome	1.579751246	UP	2.989183049
7278425	HAPS_RS07820	rpmC	MULTISPECIES: 50S ribosomal protein L29	Ribosome	1.675799119	UP	3.194962767
7278166	rpmG	rpmG	MULTISPECIES: 50S ribosomal protein L33	Ribosome	-1.129883585	DOWN	0.456952596
7278426	HAPS_RS07825	rpsQ	30S ribosomal protein S17	Ribosome	1.400550991	UP	2.640023902
7278741	rpmH	rpmH	MULTISPECIES: 50S ribosomal protein L34	Ribosome	-1.078295064	DOWN	0.473588166
7278921	HAPS_RS05815	rpsU	MULTISPECIES: 30S ribosomal protein S21	Ribosome	-1.563858769	DOWN	0.338245168
