# Supplementary figures and images for: Comparative transcriptional profiling of tildipirosin-resistant and sensitive Haemophilus parasuis
Source: Sci Rep. 2017 Aug 8;7:7517. doi: 10.1038/s41598-017-07972-5 (PMC5548900; doi:10.1038/s41598-017-07972-5)

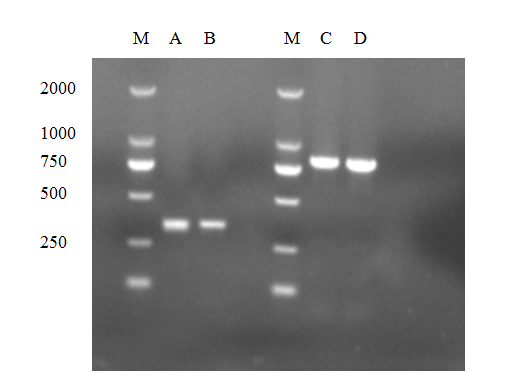

Supplement: Supplementary file 7 — Figure. S1 [file 41598_2017_7972_MOESM7_ESM.tif]

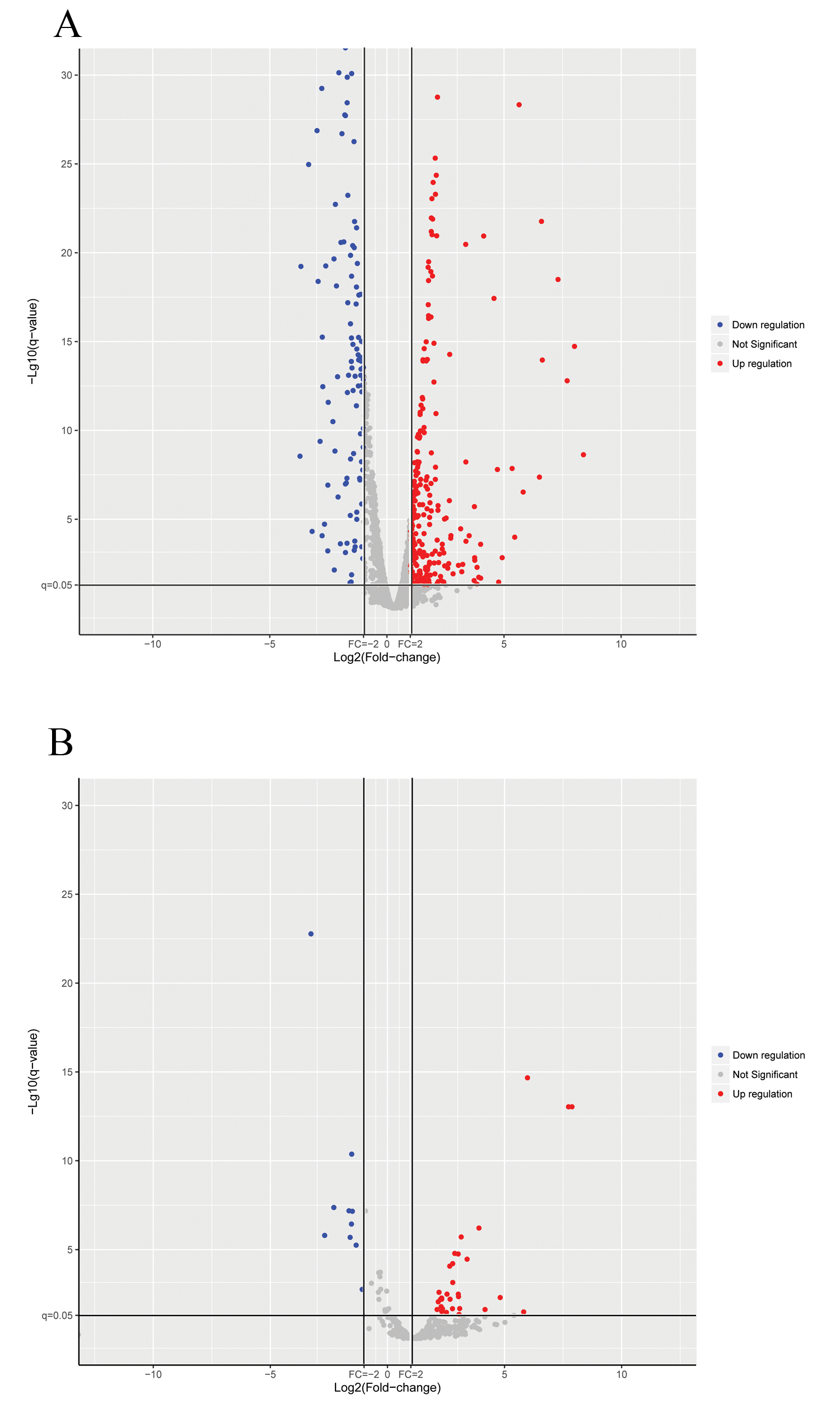

Supplement: Supplementary file 8 — Figure. S2 [file 41598_2017_7972_MOESM8_ESM.tif]
